# Hydroxyl-Directed Regio- and Diastereoselective Allylic Sulfone Reductions with [Sm(H_2_O)_*n*_]I_2_

**DOI:** 10.1021/acs.joc.3c01647

**Published:** 2023-12-13

**Authors:** Cody L. Schwans, Trevor D. Clark, Gregory W. O’Neil

**Affiliations:** Department of Chemistry, Western Washington University, Bellingham, Washington 98225, United States

## Abstract

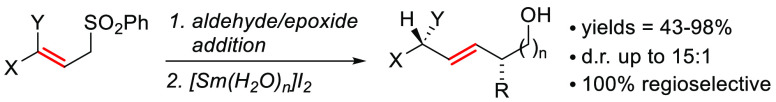

Allylic 1,2- and 1,3-hydroxy phenyl sulfones undergo regioselective and diastereoselective desulfonylation with double bond migration upon treatment with [Sm(H_2_O)_*n*_]I_2_. Selectivity in these reactions is thought to arise from the formation of a chelated organosamarium intermediate followed by intramolecular protonation by samarium-bound water, which is supported by observed diastereoselectivity and stereospecificity trends along with deuterium labeling experiments. The reaction was then featured in the synthesis of the phenolic fragment of the thailandamide natural products.

The connection between stereochemistry and function is well established. Examples range from biologically active small organic molecules^1^ to large macromolecules like proteins^2^ and polymers.^3^ Common to many of these structures are asymmetric carbon atoms, wherein carbon is substituted by four different groups. The orientation of groups attached to asymmetric carbon atoms may be directly (e.g., containing functional groups that interact directly with the target) or indirectly (e.g., confer a specific overall conformation) responsible for the observed molecular function. Methods providing predictable access to stereodefined asymmetric carbon atoms are, therefore, of great value to the synthetic chemist.

Our group has been exploring a samarium diiodide (SmI_2_) mediated elimination/isomerization process for asymmetric carbon atom synthesis.^4−6^ We have found that upon treatment with SmI_2_ and water ([Sm(H_2_O)_*n*_]I_2_),^7,8^ substrates of type **A** are transformed into alkene products **B** in high yield and in some cases high diastereoselectivity (up to 90:10; Scheme 1). The reaction is thought to proceed through a chelated organosamarium intermediate (**Sm–I**), formed upon the reduction and loss of the benzoyl ester (OBz). The existing stereocenter directs the facial selectivity of intramolecular protonation by a samarium-bound water. This model is consistent with the stereochemistry of the newly formed asymmetric carbon atom (*) for the major diastereomers obtained.^4−6^

**Scheme 1:**
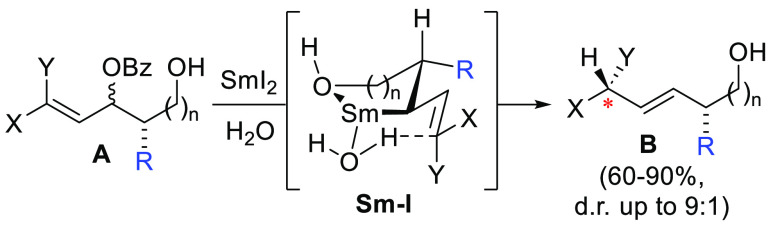
Allylic Benzoate Reductions with [Sm(H_2_O)_*n*_]I_2_ via Organosamarium Intermediate **Sm–I**

To expand the scope of the reaction, we considered other possible substrates. Phenyl sulfone (SO_2_Ph) groups are known to be reduced and eliminated by SmI_2_,^9−14^ albeit at slower rates than OBz.^15^ Replacement of the OBz in **A** with an SO_2_Ph leads to compounds of type **C** (Figure 1). We were attracted to these compounds as potential substrates for [Sm(H_2_O)_*n*_]I_2_ elimination/isomerization reactions, anticipating they could be accessed by addition of an alpha-sulfonyl anion to an aldehyde.^16^ Herein we describe results from reactions of allylic phenyl sulfonates with [Sm(H_2_O)_*n*_]I_2_. The data indicate that both the original OBz compounds and this new class of substrates react via a similar mechanism, converging to a common organosamarium intermediate and leading to high levels of predictable stereocontrol.

**Figure 1:**
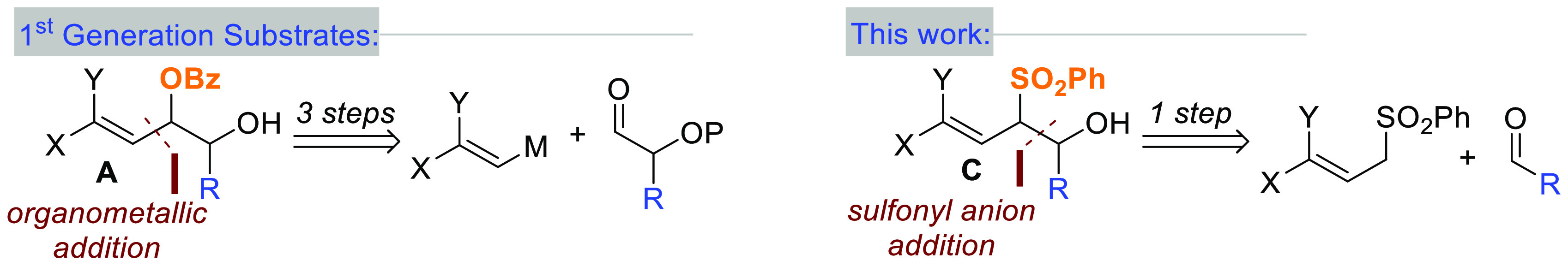
Comparison of previously investigated OBz compounds **A** and proposed phenyl sulfonyl substrates **C**.

To begin, allylic sulfone substrates **2a**–**f** were prepared by deprotonation of **1**(17) with *n*-butyllithium (BuLi), followed by aldehyde addition (Table 1). Previously, we only tested a substrate of this type containing a methyl stereodirecting group (R = CH_3_) for the OBz series, due in part to the lengthier synthetic sequence required to access those compounds.^18^ As shown in Table 1, all substrates reacted under our previously optimized conditions^19^ to afford the corresponding elimination/isomerization product. There is a clear trend between increasing the steric bulk of the stereodirecting group (R) and diastereoselectivity. With the largest stereodirecting group tested (R = C(CH_3_)_3_), **3f** was obtained as a 15.1:1 mixture of diastereomers (d.r.) based on ^1^H NMR analysis. Conversely, **2a** where R = Me gave **3a** with a much lower d.r. (6.7:1). Interestingly, **3a** was obtained from the OBz analogue in nearly identical d.r. (6.1:1) and with the same sense of diastereoselection,^5^ supporting the formation of a common intermediate from the two substrate classes. The stereochemistry of the major diastereomer of **3a** was previously determined when produced from the OBz analogue.^5^ By analogy, we assume that the stereochemistry of the major diastereomer for the products contained in Table 1 is as drawn.

**Table 1:**
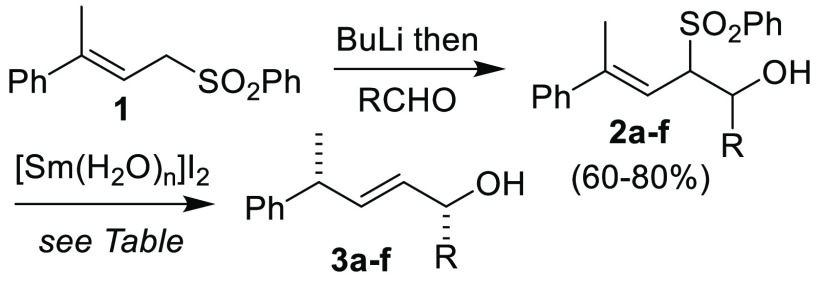
[Sm(H_2_O)_*n*_]I_2_ Reductions of **2a**–**f**^a^

compound	R	d.r.^b^	yield^c^
**3a**	CH_3_	6.7:1	43
**3b**	CH_2_CH_3_	10.5:1	59
**3c**	(CH_2_)_4_CH_3_	12.1:1	59
**3d**	CH(CH_3_)_2_	12.7:1	72
**3e**	CH_2_CH(CH_3_)_2_	13.5:1	58
**3f**	C(CH_3_)_3_	15.1:1	59

a All reactions were performed using 105 equiv of H_2_O and 7 equiv of SmI_2_ in degassed THF at rt under N_2_.

b Determined by ^1^H NMR.

c Isolated yield.

Performing the reaction of **2e** with D_2_O resulted in formation of **3e-***d*, with selective incorporation of deuterium at the newly formed asymmetric carbon atom (Scheme 2). The stereochemistry of the sulfone group proved to be unimportant, with **2e** used as a single diastereomer or mixture of diastereomers giving **3e** with the same d.r. and yield.^20^ Altogether, the results are consistent with the formation of intermediate **Sm–II**.

**Scheme 2:**
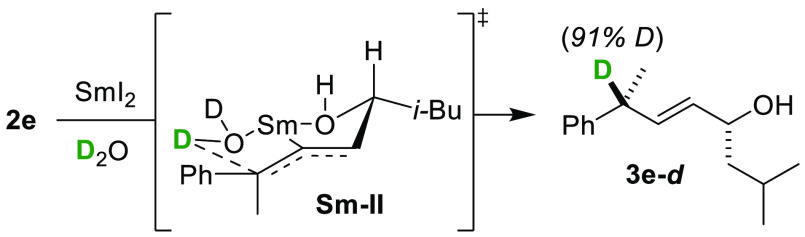
Synthesis of **3e-***d* Using [Sm(D_2_O)_*n*_]I_2_

Like for the -OBz series,^6^ stereospecificity with respect to alkene geometry was similarly substrate dependent for the sulfones. Geraniol and nerol-derived substrates (*E*-**4** and *Z*-**4** respectively) selected for opposite diastereomers of **5** upon treatment with [Sm(H_2_O)_*n*_]I_2_ (Scheme 3, eq 1) whereas mixtures of **2e** containing different *E*:*Z* ratios gave the same major diastereomer of **3e** with identical selectivity (eq 2). Previously we explained these differences by considering intermediate **Sm–III** being favored when X or Y = phenyl, which provides resonance stabilization to the Sm-bound carbanion,^6^ allowing for bond rotation and convergence of *E*/*Z*-isomers.

**Scheme 3:**
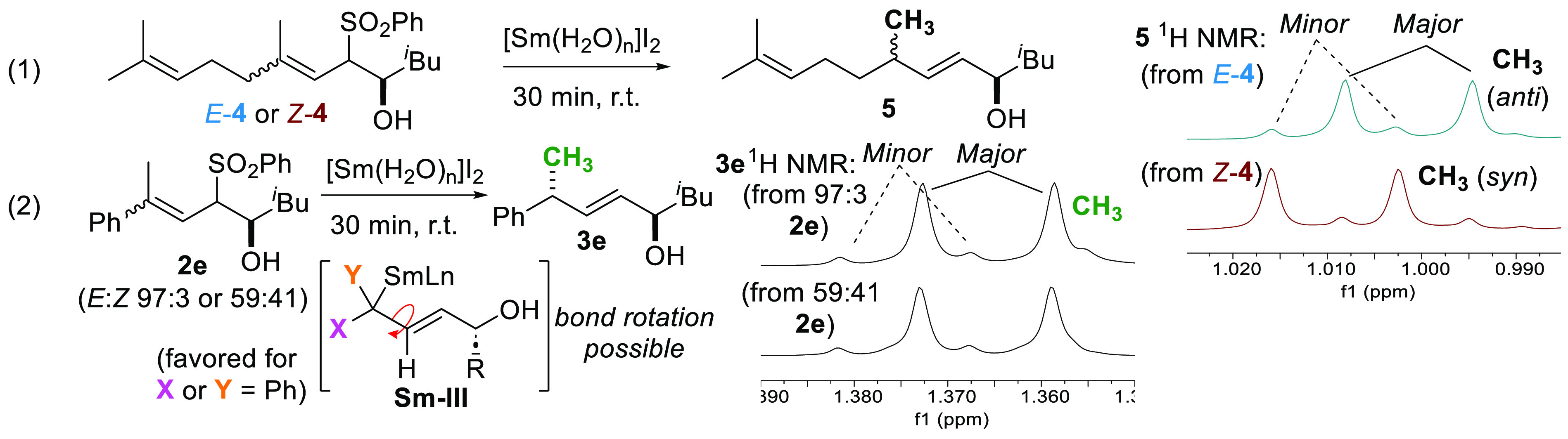
Substrate-Dependent Stereospecificity in [Sm(H_2_O)_*n*_]I_2_ Reductions of Allylic Sulfones

Other electrophiles than aldehydes will react with α-sulfonyl anions that might provide an alternative approach to preparing substrates for our sulfone-based samarium reductions.^21^ One example is epoxides, which have featured in several complex natural product syntheses.^22^ We therefore investigated the reaction of sulfone **1** with (*R*)-propylene oxide, (*R*)-styrene oxide, and triisopropylsilyl (TIPS)-protected glycidol,^23^ allowing for not only a comparison of different stereodirecting groups in subsequent [Sm(H_2_O)_*n*_]I_2_ reductions, but as an entryway to enantio- and diastereomerically enriched products (Scheme 4). Each of the resulting 1,3-hydroxy sulfones **6**–**8** reacted cleanly with [Sm(H_2_O)_*n*_]I_2_, affording the corresponding homoallylic alcohol products **9**–**11** respectively with good yield and complete regioselectivity by NMR analysis. The highest d.r. (4.4:1) was obtained from **7**, presumably due to sterics associated with the phenyl ring in the transition state. However, the nearly identical d.r. obtained from **6** and **8** (2.3:1 vs 2.5:1) could indicate an additional role for the phenyl group, as yet another example of unique reactivity of phenyl-containing compounds in reactions with SmI_2_.^24^ Diastereoselectivity within this series was overall lower than what was obtained for the aldehyde-derived substrates, consistent with previous observations that diastereoselectivity is maximized when the stereodirecting group (R) is closer to the newly formed asymmetric carbon atom. Ozonolysis of **9**–**11** produced aldehyde **12** enriched in the (*R*)-(−)-enantiomer according to polarimetry,^25^ indicating the same sense of diastereoselection for each substrate **6**–**8**.

**Scheme 4:**
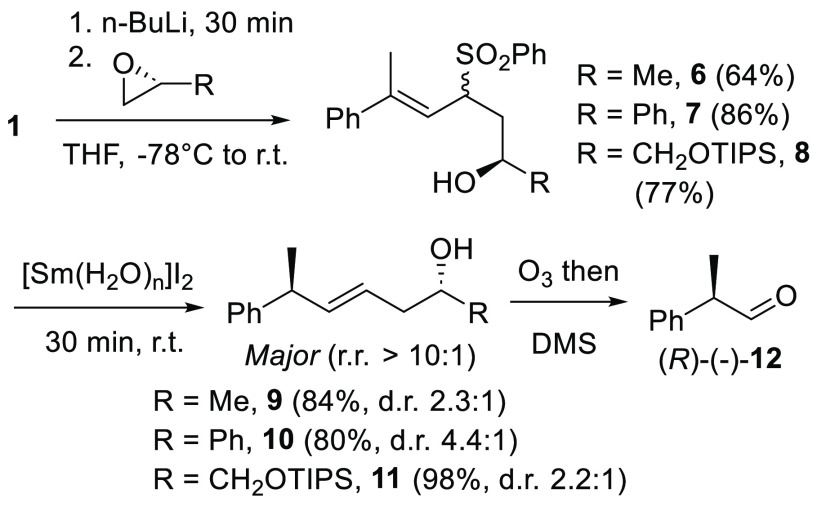
Sulfonyl Anion Epoxide Opening and Subsequent [Sm(H_2_O)_*n*_]I_2_ Reduction

Reduction of **6** proceeded with the same diastereoselectivity as its OBz analogue **13**(5) (Scheme 5), giving **9** in both cases as an approximately 70:30 mixture of diastereomers.^5^ Previously the major diastereomer produced in these reactions was identified as the 2*R*,6*S*-isomer,^5^ explained by a mechanism involving formation of intermediate **Sm–IV** where the methyl group (Me) assumes a preferred equatorial position.^26^ Formation of **9-***d* when D_2_O was used further supports this proposed mechanism.

**Scheme 5:**
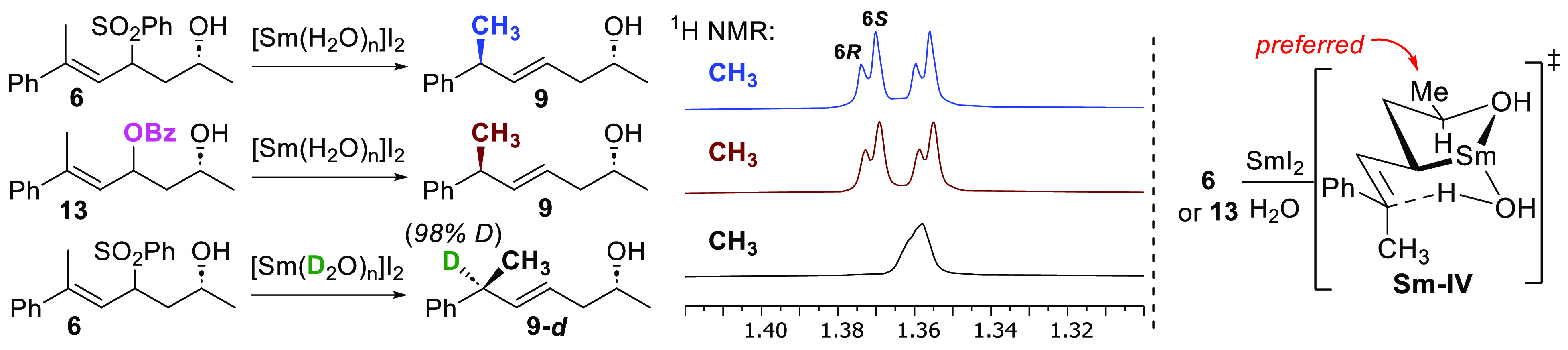
Comparison of Diastereoselectivity from the Reduction of Compounds **6** and **13**

The reduction of (2*R*)-**7** was examined using different alcohols in place of water (Table 2), since the nature of these additives can influence the mechanism^27^ and outcome of reactions with SmI_2_.^28−40^ Chiral amino diol **14**(41) along with (*R*)- and (*S*)-BINOL^42^ were also included, which have been reported for enantioselective Sm(II)-based transformations. Replacement of water in all cases caused a significant drop in conversion even with extended reaction times (the mass balance in all cases was unreacted starting material) and no practical improvement to d.r. when compared with water (2.3:1). Slightly higher d.r. values for (*R*)- versus (*S*)-BINOL (3.3:1 vs 2.3:1) and (*R*,*R*)-**14** versus (*S*,*S*)-**14** could suggest cases of matched/mismatched stereoselection. However, all reactions in Table 2 selected for the (6*S*)-isomer of **10**, indicating that none of the chiral alcohols tested were able to override the inherent stereochemical bias of the substrate.

**Table 2:**
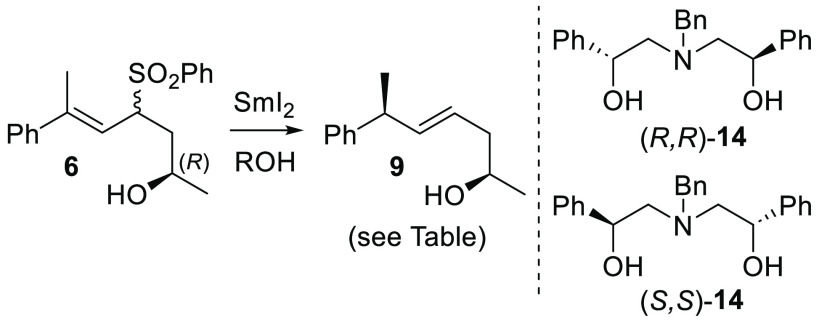
SmI_2_ Reductions of **6** with Different Proton Sources

proton source	time (h)	% conversion^d^	d.r.^d^
H_2_O^a^	0.5	100	2.3:1
MeOH^a^	1	19	1.8:1
MeOH^a^	15	30	1.8:1
*i*-PrOH^a^	15	19	1.7:1
*t*-BuOH^a^	15	17	1.9:1
(*R*,*R*)-**14**^b^	15	22	2.2:1
(*R*,*R*)-**14**, MeOH^c^	15	22	2.3:1
(*R*,*R*)-**14**, MeOH^c^	40	23	2.3:1
(*S*,*S*)-**14**, MeOH^c^	40	27	1.8:1
(*S*)-BINOL^b^	65	14	2.2:1
(*R*)-BINOL^b^	65	12	3.3:1

a Reactions were performed by adding the proton source (105 equiv) to SmI_2_ (7 equiv) followed by **7**.

b Performed by adding **14** or BINOL (7.2 equiv) to SmI_2_ (7 equiv) followed by **7**.

c Methanol (7 equiv) was added prior to **7**.

d Determined by NMR.

While a primary focus has been understanding the diastereoselectivity of this transformation, the regioselectivity and exclusive *trans*-double bond formation add to its synthetic appeal. As an example of its use in target synthesis, we set out to synthesize a fragment of the antimicrobial thailandamide natural products (Figure 2). First identified in 2008 as part of a *trans*-acyltransferase guided genome mining campaign,^43^ thailandamide A was more recently shown to represent only the second class of natural products that inhibits bacterial growth by targeting the AccA/AccD complex of the acetyl-CoA carboxylase enzyme.^44^ We hypothesized that compound **15**, a precursor to the amide-linked phenolic portion of both thailandamides A and B,^45^ could be prepared by a regioselective allylic sulfone reduction with [Sm(H_2_O)_*n*_]I_2_.

**Figure 2:**
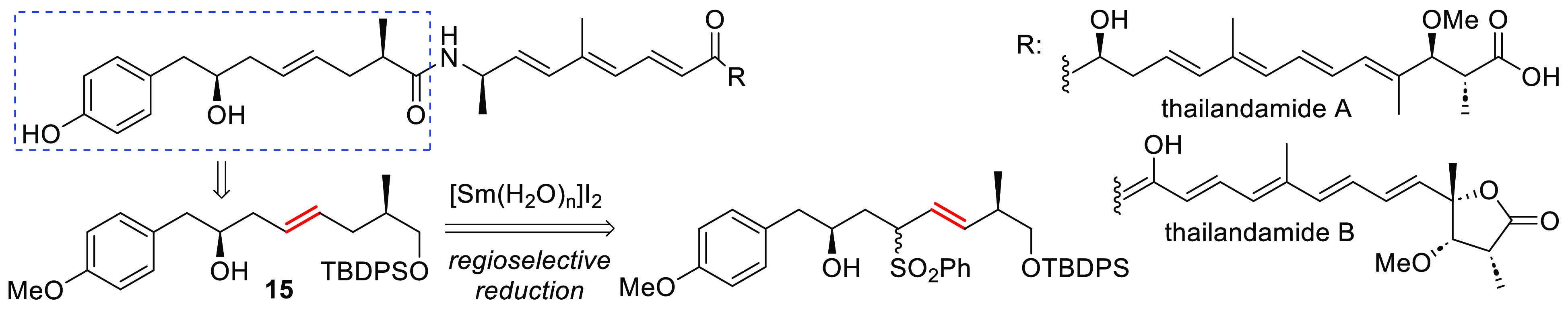
Proposed synthesis of the thailandamide phenolic fragment.

To test this, allyl sulfone **17** was first synthesized in two steps and 81% yield from the Roche ester-derived aldehyde **16**(46) by vinyl Grignard addition followed by Pd-catalyzed sulfonylation^47^ (Scheme 6). Coupling of **17** with (*R*)-configured epoxide **18** followed by [Sm(H_2_O)_*n*_]I_2_ reduction completed the synthesis of **15** in 62% yield for the two steps as a single regioisomer by ^1^H NMR analysis. We attribute the high regioselectivity in the [Sm(H_2_O)_*n*_]I_2_ reduction to the alcohol group, anchoring samarium into a preferred 5-membered chelate **Sm–V**.

**Scheme 6:**
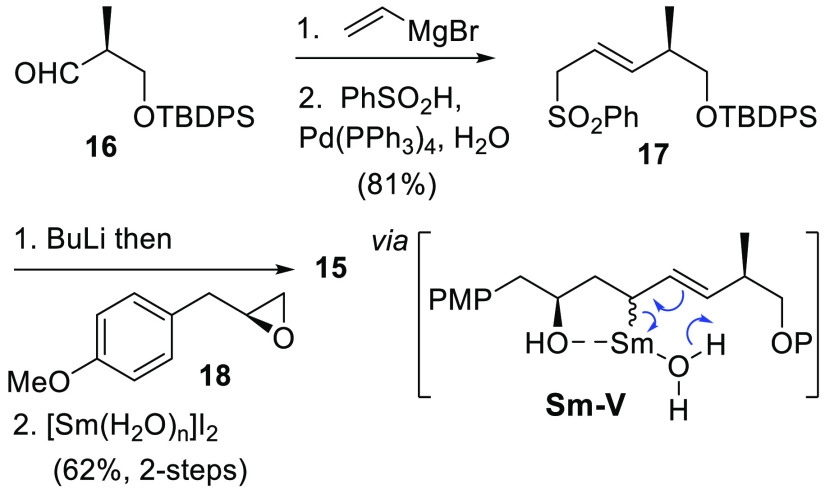
Synthesis of Fragment **15**

## Conclusion

In summary, 1,2- and 1,3-hydroxy phenyl sulfones can be reduced with [Sm(H_2_O)_*n*_]I_2_, providing desulfonylated allylic and homoallylic alcohol products in good yield, regioselectivity, and diastereoselectivity (up to 15:1). Substrates used in this study were synthesized by the addition of allyl alpha sulfonyl anions to an aldehyde or epoxide, which revealed a trend between increasing sterics and increased d.r. for the subsequent [Sm(H_2_O)_*n*_]I_2_ reductions. Similarities were observed between the phenyl sulfonyl substrates and previously investigated OBz-compounds, for example d.r. values and stereospecificity, suggesting a shared mechanism involving formation of a chelated organosamarium intermediate and intramolecular protonation by a samarium-bound water molecule, which was further supported by deuterium labeling experiments. The sequence presented has potential as a fragment coupling strategy in complex target-oriented synthesis, as evidenced by a completion of the phenolic portion of the thailandamide natural products.

## Experimental Section

### General Information

All reactions were carried out under N_2_ in flame-dried glassware. The solvents used were dried by passing the solvent through a column of activated alumina under nitrogen immediately prior to use. All reagents were purchased and used as received, unless otherwise mentioned. TLC analysis used 0.25 mm silica layer fluorescence UV_254_ plates. Flash chromatography: silica gel (230–400 mesh). NMR: Spectra were recorded on a 500 MHz spectrometer in CDCl_3_; chemical shifts (δ) are given in ppm, coupling constants (*J*) in Hz. Solvent signals were used as references (CDCl_3_ δ_c_ ≡ 77.0 ppm; residual CHCl_3_ in CDCl_3_ δ_H_ ≡ 7.26 ppm). In cases where diastereomers are produced, spectral data listed are for the mixture unless specified otherwise.

### General Experimental Procedures

#### Procedure A

Allyl phenyl sulfonyl was added to aldehydes or epoxides.

To a solution of the allylic sulfone (1.0 equiv) in THF (to make a 0.2 M solution of the sulfone) at −78 °C was added *n-*BuLi (1.2 equiv), and the resulting yellow/orange solution was stirred for 45 min. The aldehyde or epoxide (1.5 equiv) was then added and the mixture was allowed to slowly warm to room temperature over 8 h (or until completion by TLC). The reaction was quenched with aq. NH_4_Cl and extracted with methyl *tert-*butyl ether (MTBE). The organic extracts were then dried with MgSO_4_ and concentrated *in vacuo*.

#### Procedure B

SmI_2_ reduction with ROH (H_2_O, D_2_O, MeOH, *i*-PrOH, or *t*-BuOH).

To a solution of SmI_2_ in THF (0.1 M, 7.0 equiv) was added degassed ROH (105 equiv). The hydroxysulfone (1.0 equiv) was then added and the reaction mixture was stirred at room temperature for the specified time (see Tables 1 and 2). The reaction was quenched with aq. NaHCO_3_ and extracted with ethyl acetate (EtOAc). The organic extracts were washed with brine, dried with MgSO_4_, and concentrated *in vacuo*.

### Methyl 3-phenylbut-2-enoate

To a solution of LiHMDS (4.2 g, 25.0 mmol) in THF (50 mL) at 0 °C was added trimethylphosphonoacetate (6.1 g, 27.24 mmol), and the solution was allowed to warm to room temperature and stir over a 30 min period. After cooling to −78 °C, acetophenone (2.65 mL, 22.7 mmol) was added, and the mixture was allowed to slowly warm to room temperature and stirred for 15 h. The reaction was then quenched with aq. NH_4_Cl (50 mL) and extracted with MTBE (3 × 50 mL). The combined organic extracts were dried with MgSO_4_, filtered, and concentrated *in vacuo*. Purification by flash column chromatography on silica gave methyl 3-phenylbut-2-enoate (2.88 g, 72%) as an oil and a 4:1 partially separable mixture of *E*- and *Z*-stereoisomers. Spectral data matched that previously reported:^48^^1^H NMR (CDCl_3_, 500 MHz) δ = 7.50–7.45 (m, 2H), 7.40–7.35 (m, 3H), 6.14 (q, *J* = 1.4 Hz, 1H), 3.76 (s, 3H), 2.59 (d, *J* = 1.4 Hz, 3H).

### 3-Phenylbut-2-en-1-ol

To a solution of methyl 3-phenylbut-2-enoate (0.5 g, 2.84 mmol) in DCM (5.6 mL) at −78 °C was added DIBAL-H (1.0 M, 6.25 mL) and the resulting mixture was warmed to 0 °C and stirred for 1 h. The reaction was quenched with EtOAc (25 mL) and washed with aq. HCl (1.0 M, 25 mL). The aqueous phase was extracted with EtOAc (25 mL) and the organic phase was dried over MgSO_4_, filtered, and concentrated *in vacuo*. Spectral data for the crude product matched that previously reported^49^ which was used directly in the next reaction: ^1^H NMR (500 MHz, CDCl_3_) δ = 7.43–7.40 (m, 2H), 7.36–7.31 (m, 2H), 7.30–7.23 (m, 1H), 5.98 (tq, *J* = 6.7, 1.4 Hz, 1H), 4.37 (dd, *J* = 6.7, 0.9 Hz, 2H), 2.09–2.08 (m, 3H).

### ((3-Phenylbut-2-en-1-yl)sulfonyl)benzene (**1**)

To a solution of 3-phenylbut-2-en-1-ol (0.200 g, 1.35 mmol) in THF (1 mL) at 0 °C was added PBr_3_ (0.15 g, 0.54 mmol), and the mixture was stirred for 1 h. The reaction was quenched with ice-cold water (15 mL) and extracted with hexanes (3 × 15 mL). The combined organic extracts were dried with MgSO_4_ and concentrated *in vacuo*. The crude bromide was dissolved in DMF (1.5 mL), sodium benzenesulfonate (0.365 g, 2.03 mmol) was added, and the solution was heated to 100 °C for 8 h. The reaction was cooled to room temperature, diluted with EtOAc (25 mL) and washed with water (25 mL). The organic phase was dried over MgSO_4_, filtered, and concentrated *in vacuo*. Purification by flash column chromatography on silica gave **1** (0.206 g, 56%) as an oil and a partially separable mixture of *E*- and *Z*-stereoisomers. Spectral data matched that previously reported:^17^^1^H NMR (CDCl_3_, 500 MHz) δ = 7.92–7.88 (m, 2H), 7.67–7.62 (m, 1H), 7.57–7.51 (m, 2H), 7.36–7.25 (m, 5H), 5.72 (tq, *J* = 8.2, 1.4 Hz, 1H), 4.01 (d, *J* = 8.1 Hz, 2H), 1.70–1.67 (m, 3H).

### (*E*)-5-Phenyl-3-(phenylsulfonyl)hex-4-en-2-ol (**2a**)

Prepared according to the general procedure **A** using sulfone **1** (0.090 g, 0.33 mmol) and acetaldehyde (0.037 mL, 0.66 mmol). Purification by flash column chromatography on silica gave (4:1 hexanes:EtOAc) gave **2a** (0.085 g, 82%) as an oil and a 1.4:1 mixture of diastereomers. IR (ATR) 3383, 2930, 1697, 1645, 1632, 1310, 1241, 987, 972 cm^–1^. ^1^H NMR (CDCl_3_, 500 MHz) δ = 7.89–7.85 (m, 4H), 7.67–7.63 (m, 2H), 7.55–7.51 (m, 4H), 7.34–7.25 (m, 6H), 7.23–7.21 (m, 4H), 6.00 (dq, *J* = 10.9, 1.4 Hz, 1H), 5.39 (dq, *J* = 11.0, 1.4 Hz, 1H), 4.87 (qd, *J* = 6.5, 1.6 Hz, 1H), 4.58 (dq, *J* = 8.6, 6.3 Hz, 1H), 4.19 (bs, 1H), 4.01 (dd, *J* = 11.0, 8.6 Hz, 1H), 3.87 (dd, *J* = 10.9, 1.6 Hz, 1H), 3.15 (bs, 1H), 1.54 (d, *J* = 1.4 Hz, 3H), 1.53 (d, *J* = 1.5 Hz, 3H), 1.26 (d, *J* = 5.8 Hz, 3H), 1.24 (d, *J* = 5.8 Hz, 3H). *Spectral data for the major diastereomer*: ^13^C{^1^H} NMR (CDCl_3_, 126 MHz) δ = 143.8, 142.0, 137.5, 134.0, 129.07, 129.05, 128.4, 128.0, 125.7, 116.8, 71.9, 66.3, 21.0, 16.1. HRMS (ESI+) calcd *m*/*z* for C_18_H_21_O_3_S [M + H]^+^ 317.1211, found 317.1210.

### (*E*)-6-Phenyl-4-(phenylsulfonyl)hept-5-en-3-ol (**2b**)

Prepared according to the general procedure **A** using sulfone **1** (0.10 g, 0.37 mmol) and propionaldehyde (0.049 mL, 0.66 mmol). Purification by flash column chromatography on silica (4:1 hexanes:EtOAc) gave **2b** (0.078 g, 64%) as an oil and 2:1 mixture of diastereomers. IR (ATR) 3381, 2928, 2857, 1643, 1535, 1421, 1265, 1301, 1115, 998, 962, 884 cm^–1^. ^1^H NMR (CDCl_3_, 500 MHz) δ = 7.91–7.85 (m, 4H), 7.66–7.62 (m, 2H), 7.55–7.50 (m, 4H), 7.34–7.24 (m, 9H), 7.23–7.21 (m, 1H), 6.01 (dq, *J* = 10.9, 1.4 Hz, 1H), 5.40 (dq, *J* = 11.1, 1.4 Hz, 1H), 4.60 (ddd, *J* = 8.2, 5.6, 1.5 Hz, 1H), 4.40 (ddd, *J* = 8.8, 7.9, 3.1 Hz, 1H), 4.05 (dd, *J* = 11.0, 8.8 Hz, 1H), 3.93 (dd, *J* = 10.9, 1.4 Hz, 1H), 3.17 (bs, 1H), 1.52 (d, *J* = 1.5 Hz, 3H), 1.51 (d, *J* = 1.5 Hz, 3H), 1.74–1.60 (m, 2H), 1.47–1.39 (m, 2H), 0.97 (td, *J* = 7.4, 4.4 Hz, 6H). *Spectral data for the major diastereomer:*^13^C{^1^H} NMR (CDCl_3_, 126 MHz) δ = 145.1, 142.2, 137.8, 133.8, 129.0, 128.9, 128.3, 127.8, 125.8, 114.7, 70.1, 68.8, 28.0, 16.2, 10.1. HRMS (ESI+) calcd *m*/*z* for C_19_H_23_O_3_S [M + H]^+^ 331.1368, found 331.1362.

### (*E*)-2-Phenyl-4-(phenylsulfonyl)dec-2-en-5-ol (**2c**)

Prepared according to the general procedure **A** using sulfone **1** (0.10 g, 0.37 mmol) and hexanal (0.093 mL, 0.77 mmol). Purification by flash column chromatography on silica (4:1 hexanes:EtOAc) gave **2c** (0.085 g, 62%) as an oil and 2.2:1 mixture of diastereomers. IR (ATR) 3378, 2978, 2927, 1611, 1545, 1462, 1310, 1245, 1098, 972 cm^–1^. ^1^H NMR (CDCl_3_, 500 MHz) δ = 1H NMR (500 MHz, CDCl3) δ = 7.89–7.85 (m, 4H), 7.67–7.62 (m, 2H), 7.56–7.49 (m, 6H), 7.34–7.24 (m, 7H), 7.23–7.20 (m, 1H), 6.01 (dq, *J* = 11.0, 1.5 Hz, 1H), 5.40 (dq, *J* = 11.0, 1.4 Hz, 1H), 4.68 (ddd, *J* = 8.4, 5.1, 1.4 Hz, 1H), 4.45 (td, *J* = 8.2, 3.0 Hz, 1H), 4.04 (dd, *J* = 11.0, 8.7 Hz, 1H), 3.90 (dd, *J* = 10.8, 1.4 Hz, 1H), 3.09 (bs, 1H), 1.65–1.55 (m, 4H), 1.51 (d, *J* = 1.4 Hz, 3H), 1.50 (d, *J* = 1.4 Hz, 3H), 1.34–1.24 (m, 12H), 0.93–0.82 (m, 6H). *Spectral data for the major diastereomer:*^13^C{^1^H} NMR (CDCl_3_, 126 MHz) δ = 145.2, 142.2, 137.8, 133.8, 129.01, 128.96, 128.3, 127.9, 125.8, 114.8, 69.1, 68.7, 34.8, 31.5, 25.3, 22.5, 16.3, 13.9. HRMS (ESI+) calcd *m*/*z* for C_22_H_28_NaO_3_S [M + Na]^+^ 395.1657, found 395.1658.

### (*E*)-2-Methyl-6-phenyl-4-(phenylsulfonyl)hept-5-en-3-ol (**2d**)

Prepared according to the general procedure **A** using sulfone **1** (0.10 g, 0.37 mmol) and isobutyraldehyde (0.057 mL, 0.62 mmol). Purification by flash column chromatography on silica (4:1 hexanes:EtOAc) gave **2d** (0.085 g, 67%) as an oil and 3.6:1 mixture of diastereomers. *Spectral data for the major diastereomer*: IR (ATR) 3382, 2972, 2965, 1643, 1551, 1475, 1310, 1250, 1098, 972 cm^–1^. ^1^H NMR (CDCl_3_, 500 MHz) δ = 7.87–7.85 (m, 2H), 7.67–7.64 (m, 1H), 7.55–7.51 (m, 2H), 7.34–7.29 (m, 3H), 7.23–7.21 (m, 2H), 5.39 (dq, *J* = 11.0, 1.4 Hz, 1H), 4.34 (dd, *J* = 9.2, 2.2 Hz, 1H), 4.07 (bs, 1H), 4.05 (dd, *J* = 11.1, 9.2 Hz, 1H), 1.78 (pd, *J* = 6.8, 2.1 Hz, 1H), 1.49 (d, *J* = 1.4 Hz, 3H), 1.08 (d, *J* = 6.9 Hz, 3H), 0.81 (d, *J* = 6.7 Hz, 3H). ^13^C{^1^H} NMR (CDCl_3_, 126 MHz) δ = 143.1, 141.9, 137.5, 134.0, 129.3, 129.0, 128.5, 128.0, 125.7, 116.6, 72.7, 70.1, 31.1, 20.0, 15.8, 13.7. HRMS (ESI+) calcd *m*/*z* for C_20_H_25_O_3_S [M + H]^+^ 345.1524, found 345.1524.

### (*E*)-2-Methyl-7-phenyl-5-(phenylsulfonyl)oct-6-en-4-ol (**2e**)

Prepared according to general procedure **A** using sulfone **1 (**0.103 g, 0.379 mmol) and isovaleraldehyde (0.083 mL, 0.76 mmol). Purification by flash column chromatography on silica (4:1 hexanes:EtOAc) gave **2e** (0.090 g, 66%) as an oil and a 1.6:1 mixture of diastereomers. IR (ATR) 3530, 3060, 2955, 2870, 1737, 1584, 1494, 1467, 1446, 1371, 1297, 1240, 1146, 1082, 1049, 1025, 999, 881, 849, 757, 687, 630 cm^–1^. ^1^H NMR (CDCl_3_, 500 MHz) δ = 7.88–7.85 (m, 4H), 7.66–7.62 (m, 2H), 7.54–7.50 (m, 4H), 7.35–7.25 (m, 6H), 7.24–7.22 (m, 4H), 6.02 (dq, *J* = 10.9, 1.4 Hz, 1H), 5.42 (dq, *J* = 11.2, 1.5 Hz, 1H), 4.81 (ddd, *J* = 9.3, 4.5, 1.4 Hz, 1H), 4.52 (ddd, *J* = 10.8, 8.5, 2.5 Hz, 1H), 4.02 (dd, *J* = 8.5, 2.0 Hz, 1H), 3.87 (dd, *J* = 10.9, 1.4 Hz, 1H), 3.08 (bs, 1H), 1.97 (th, *J* = 10.4, 3.4 Hz, 1H), 1.83–1.73 (m, 1H), 1.59–1.54 (m, 2H), 1.42 (ddd, *J* = 14.2, 10.5, 3.7 Hz, 2H), 1.51 (d, *J* = 1.4 Hz, 3H), 1.47 (d, *J* = 1.4 Hz, 3H), 0.93 (d, *J* = 6.5 Hz, 6H), 0.91 (d, *J* = 6.7 Hz, 3H), 0.91 (d, *J* = 6.9 Hz, 3H). *Spectral data for the major diastereomer*: ^13^C{^1^H} NMR (CDCl_3_, 126 MHz) δ = 145.1, 142.2, 137.8, 133.8, 129.0, 128.9, 128.3, 127.9, 125.8, 114.8, 69.3, 66.7, 43.7, 24.3, 23.1, 21.9, 16.2. HRMS (ESI+) calcd *m*/*z* for C_21_H_27_O_3_S [M + H]^+^ 359.1681, found 359.1685.

### (*E*)-2,2-Dimethyl-6-phenyl-4-(phenylsulfonyl)hept-5-en-3-ol (**2f**)

Prepared according to general procedure **A** using sulfone **1** (0.084 g, 0.31 mmol) and pivaldehyde (0.069 mL, 0.62 mmol). Purification by flash column chromatography on silica (4:1 hexanes:EtOAc) gave **2f** (0.067 g, 60%) as an oil and a 7.2:1 mixture of diastereomers. *Spectral data for the major diastereomer:* IR (ATR) 3432, 3028, 2957, 2928, 1594, 1498, 1476, 1452, 1298, 1240, 1146, 1082, 1049, 1025, 882 cm^–1^. ^1^H NMR (CDCl_3_, 500 MHz) δ = 7.87–7.82 (m, 2H), 7.64 (ddt, *J* = 8.7, 7.1, 1.3 Hz, 1H), 7.54–7.48 (m, 2H), 7.33–7.25 (m, 3H), 7.23–7.19 (m, 2H), 6.09 (dq, *J* = 10.6, 1.4 Hz, 1H), 4.46 (s, 1H), 4.11 (dd, *J* = 10.8, 0.9 Hz, 1H), 2.96 (s, 1H), 1.45 (d, *J* = 1.5 Hz, 3H), 0.96 (s, 9H). ^13^C{^1^H} NMR (CDCl_3_, 126 MHz) δ = 143.0, 142.5, 137.2, 133.8, 129.4, 128.8, 128.3, 127.7, 125.7, 116.5, 74.6, 66.6, 35.8, 26.6, 16.3. HRMS (ESI+) calcd *m*/*z* for C_21_H_26_NaO_3_S [M + Na]^+^ 381.1500, found 381.1501.

### (*E*)-5-Phenylhex-3-en-2-ol (**3a**)

Prepared according to general procedure **B** using **2a** (0.080 g, 0.25 mmol). Purification by flash column chromatography on silica (4:1 hexanes:EtOAc) gave **3a** (0.019 g, 43%) as an oil and a 6.7:1 mixture of diastereomers. Spectral data matched that previously reported:^5^^1^H NMR (CDCl_3_, 500 MHz) δ = 7.32–7.28 (m, 2H), 7.22–7.18 (m, 3H), 5.82 (ddt, *J* = 15.4, 6.6, 1.1 Hz, 1H), 5.56 (ddt, *J* = 15.5, 6.6, 1.1 Hz, 1H), 4.30 (p, *J* = 6.4 Hz, 1H), 3.46 (p, *J* = 7.0 Hz, 1H), 1.55 (bs, 1H), 1.36 (dd, *J* = 7.0, 0.9 Hz, 3H), 1.28–1.25 (m, 3H). ^13^C{^1^H} NMR (CDCl_3_, 126 MHz) δ = 145.6, 135.4, 132.9, 128.4, 127.2, 126.2, 68.9, 41.8, 23.4, 21.2.

### (*E*)-6-Phenylhept-4-en-3-ol (**3b**)

Prepared according to general procedure **B** using **2b** (0.059 g, 0.18 mmol). Purification by flash column chromatography on silica (4:1 hexanes:EtOAc) gave **3b** (0.020 g, 59%) as an oil and a 10.5:1 mixture of diastereomers. *Spectral data for the major diastereomer*: IR (ATR) 3347, 2958, 2925, 2871, 2857, 1606, 1457, 1371, 1258, 1150, 1123, 1060, 969, 730 cm^–1^. ^1^H NMR (CDCl_3_, 500 MHz) δ = 7.32–7.28 (m, 2H), 7.23–7.18 (m, 3H), 5.84 (ddd, *J* = 15.5, 6.6, 1.1 Hz, 1H), 5.50 (ddd, *J* = 15.4, 7.0, 1.4 Hz, 1H), 4.01 (q, *J* = 6.6, 1H), 3.48 (p, *J* = 7.0 Hz, 1H), 1.65–1.46 (m, 2H), 1.37 (d, *J* = 7.0 Hz, 3H), 0.92 (t, *J* = 7.5 Hz, 3H). ^13^C{^1^H} NMR (CDCl_3_, 126 MHz) δ = 145.6, 136.6, 131.5, 128.4, 127.2, 126.2, 74.4, 42.0, 30.2, 21.2, 9.8. HRMS (ESI+) calcd *m*/*z* for C_13_H_19_O [M + H]^+^ 191.1436, found 191.1436.

### (*E*)-2-Phenyldec-3-en-5-ol (**3c**)

Prepared according to general procedure **B** using **2c** (0.058 g, 0.15 mmol). Purification by flash column chromatography on silica (4:1 hexanes:EtOAc) gave **3c** (0.021 g, 59%) as an oil and a 12.1:1 mixture of diastereomers. *Spectral data for the major diastereomer*: IR (ATR) 3360, 3083, 3061, 3025, 2961, 2925, 2871, 1950, 1876, 1803, 1716, 1601, 1492, 1415, 1373, 1272, 1029, 971, 760, 698 cm^–1^. ^1^H NMR (CDCl_3_, 500 MHz) δ = 7.32–7.29 (m, 2H), 7.23–7.18 (m, 3H), 5.82 (ddd, *J* = 15.5, 6.6, 1.0 Hz, 1H), 5.51 (ddd, *J* = 15.4, 7.1, 1.4 Hz, 1H), 4.08 (q, *J* = 6.7 Hz, 1H), 3.47 (p, *J* = 7.0 Hz, 1H), 1.63–1.44 (m, 4H), 1.36 (d, *J* = 7.0 Hz, 3H), 1.33–1.27 (m, 4H), 0.92–0.86 (m, 3H). ^13^C{^1^H} NMR (CDCl_3_, 126 MHz) δ = 145.6, 136.4, 131.8, 128.4, 127.2, 126.2, 73.1, 41.9, 37.3, 31.7, 25.2, 22.6, 21.2, 14.0. HRMS (ESI+) calcd *m*/*z* for C_16_H_24_NaO [M + Na]^+^ 255.1725, found 255.1719.

### (*E*)-2-Methyl-6-phenylhept-4-en-3-ol (**3d**)

Prepared according to the general procedure **B** using **2d** (0.034, 0.098 mmol). Purification by flash column chromatography on silica (4:1 hexanes:EtOAc) gave **3d** (0.014 g, 72%) as an oil and a 12.7:1 mixture of diastereomers. *Spectral data for the major diastereomer*: IR (ATR) 3462, 3062, 2956, 2929, 2871, 1714, 1600, 1578, 1450, 1315, 1266, 1111, 1068, 962, 709 cm^–1^. ^1^H NMR (CDCl_3_, 500 MHz) δ = 7.32–7.28 (m, 2H), 7.23–7.18 (m, 3H), 5.83 (ddd, *J* = 15.5, 6.6, 1.0 Hz, 1H), 5.51 (ddd, *J* = 15.5, 7.3, 1.4 Hz, 1H), 3.82 (t, *J* = 6.7, 1H), 3.53–3.45 (m, 1H), 1.73 (dq, *J* = 13.4, 6.7 Hz, 1H), 1.37 (d, *J* = 7.0 Hz, 3H), 0.94 (d, *J* = 6.8 Hz, 3H), 0.90 (d, *J* = 6.8 Hz, 3H). ^13^C{^1^H} NMR (CDCl_3_, 126 MHz) δ = 145.6, 137.4, 130.0, 128.4, 127.2, 126.1, 78.2, 42.1, 33.9, 21.3, 18.3, 18.2. HRMS (ESI+) calcd *m*/*z* for C_14_H_20_NaO [M + Na]^+^ 227.1412, found 227.1409.

### (*E*)-2-Methyl-7-phenyloct-5-en-4-ol (**3e**)

Prepared according to general procedure **B** using **2e** (0.078 g, 0.22 mmol). Purification by flash column chromatography on silica (4:1 hexanes:EtOAc) gave **3e** (0.028 g, 58%) as an oil and a 13.5:1 mixture of diastereomers. *Spectral data for major diastereomer*: IR (ATR) 3347, 2958, 2925, 2871, 2857, 1606, 1457, 1371, 1258, 1150, 1123, 1060, 969, 730 cm^–1^. ^1^H NMR (CDCl_3_, 500 MHz) δ = 7.33–7.28 (m, 2H), 7.22–7.19 (m, 3H), 5.84 (ddd, *J* = 15.4, 6.6, 1.0 Hz, 1H), 5.50 (ddd, *J* = 15.4, 7.1, 1.4 Hz, 1H), 4.16 (tdd, *J* = 6.9, 5.8, 1.0 Hz, 1H), 3.47 (p, *J* = 7.0 Hz, 1H), 1.73 (dtd, *J* = 13.1, 6.6, 1.1 Hz, 1H), 1.49 (ddd, *J* = 13.6, 7.9, 6.5 Hz, 1H), 1.37 (d, *J* = 7.1 Hz, 3H), 1.35–1.29 (m, 1H), 0.93 (dd, *J* = 7.5, 6.6 Hz, 6H). ^13^C{^1^H} NMR (CDCl_3_, 126 MHz) δ = 145.6, 136.1, 132.1, 128.4, 127.2, 126.2, 71.2, 46.4, 41.9, 24.6, 22.9, 22.4, 21.2. HRMS (ESI+) calcd *m*/*z* for C_15_H_22_NaO [M + Na]^+^ 241.1568, found 241.1563.

### (*E*)-2-Methyl-7-phenyloct-5-en-7-d-4-ol (**3**e-*d*)

Prepared according to general procedure **B** using **2e** (0.067 g, 0.167 mmol) and D_2_O (0.39 mL). Purification by flash column chromatography on silica (4:1 hexanes:EtOAc) gave **3e-***d* (0.021 g, 57%) as an oil. ^1^H NMR (500 MHz, CDCl3) δ 7.33–7.28 (m, 2H), 7.22–7.18 (m, 3H), 5.83 (dd, *J* = 15.5, 1.0 Hz, 1H), 5.50 (dd, *J* = 15.5, 7.1 Hz, 1H), 4.16 (dddd, *J* = 8.0, 7.0, 5.9, 1.0 Hz, 1H), 1.73 (dh, *J* = 7.6, 6.6 Hz, 1H), 1.49 (ddd, *J* = 13.6, 7.9, 6.5 Hz, 1H), 1.35 (s, 3H), 1.37–1.29 (m, 1H), 0.93 (d, *J* = 6.6 Hz, 3H), 0.92 (d, *J* = 6.7 Hz, 3H). ^13^C{^1^H} NMR (126 MHz, CDCl_3_) δ 145.6, 136.1, 132.2, 128.5, 128.5, 127.2, 126.2, 71.3, 46.5, 41.7, 41.6, 41.4, 24.6, 23.0, 22.5, 21.2.

### (*E*)-2,2-Dimethyl-6-phenylhept-4-en-3-ol (**3f**)

Prepared according to general procedure **B** using **2f** (0.033 g, 0.092 mmol). Purification by flash column chromatography on silica (4:1 hexanes:EtOAc) gave **3f** (0.012 g, 59%) as an oil and a 15.1:1 mixture of diastereomers. *Spectral data for the major diastereomer*: IR (ATR) 3436, 3055, 2963, 2873, 1599, 1597, 1512, 1441, 1365, 1255, 1108, 1032, 909, 732 cm^–1^. ^1^H NMR (CDCl_3_, 500 MHz) δ = 7.33–7.28 (m, 2H), 7.23–7.18 (m, 3H), 5.83 (ddd, *J* = 15.4, 6.6, 1.0 Hz, 1H), 5.57 (ddd, *J* = 15.5, 7.6, 1.4 Hz, 1H), 3.73 (d, *J* = 7.6, 1H), 3.49 (p, *J* = 7.0 Hz, 1H), 1.37 (d, *J* = 7.1 Hz, 3H), 0.91 (s, 9H). ^13^C{^1^H} NMR (CDCl_3_, 126 MHz) δ = 145.7, 138.0, 128.6, 128.4, 127.2, 126.1, 81.0, 42.2, 34.9, 25.8, 21.3. HRMS (ESI+) calcd *m*/*z* for C_15_H_22_NaO [M + Na]^+^ 241.1568, found 241.1562.

### (*E*)-2,7,11-Trimethyl-5-(phenylsulfonyl)dodeca-6,10-dien-4-ol (*E*-**4**)

Prepared according to general procedure **A** using (*E*)-((3,7-dimethylocta-2,6-dien-1-yl)sulfonyl)benzene^50^ (0.094 g, 0.34 mmol) and isovaleraldehyde (0.074 mL, 0.68 mmol). Purification by flash column chromatography on silica (4:1 hexanes:EtOAc) gave *E***-4** (0.118 g, 95%) as an oil and a 1.9:1 mixture of diastereomers. IR (ATR) 3528, 3080, 2955, 2898, 1582, 1494, 1466, 1447, 1370, 1298, 1240, 1145, 1082, 998, 962, 757 cm^–1^. ^1^H NMR (CDCl_3_, 500 MHz) δ = 7.86–7.82 (m, 4H), 7.66–7.62 (m, 2H), 7.55–7.51 (m, 4H), 5.45 (dt, *J* = 10.7, 1.4 Hz, 1H), 5.02–4.98 (m, 2H), 4.86 (dq, *J* = 11.0, 1.4 Hz, 1H), 4.66 (ddd, *J* = 8.9, 4.7, 1.4 Hz, 1H), 4.35 (ddd, *J* = 10.7, 8.5, 2.6 Hz, 1H), 4.00 (bs, 1H), 3.84 (dd, *J* = 11.0, 8.6 Hz, 1H), 3.68 (dd, *J* = 10.7, 1.4 Hz, 1H), 2.97 (bs, 1H), 2.00–1.90 (m, 8H), 1.68 (s, 6H), 1.58 (s, 6H), 1.51–1.46 (m, 1H), 1.32 (ddd, *J* = 14.0, 10.5, 3.7 Hz, 2H), 1.19 (ddd, *J* = 14.0, 10.2, 2.6 Hz, 2H), 1.08–1.03 (m, 1H), 1.15 (d, *J* = 1.4 Hz, 3H), 1.11 (d, *J* = 1.4 Hz, 3H), 0.91 (d, *J* = 6.6 Hz, 6H), 0.90 (d, *J* = 6.7 Hz, 6H). *Spectral data for major diastereomer*: ^13^C{^1^H} NMR (CDCl_3_, 126 MHz) δ = 145.5, 137.7, 133.8, 132.2, 129.1, 128.8, 123.4, 114.2, 70.9, 67.7, 43.4, 39.6, 25.9, 25.7, 23.93, 23.88, 21.1, 17.7, 16.2. HRMS (ESI+) calcd *m*/*z* for C_21_H_32_NaO_3_S [M + Na]^+^ 387.1970, found 387.1970.

### (*Z*)-2,7,11-Trimethyl-5-(phenylsulfonyl)dodeca-6,10-dien-4-ol (*Z*-**4**)

Prepared according to general procedure **A** using (*Z*)-((3,7-dimethylocta-2,6-dien-1-yl)sulfonyl)benzene^51^ (0.100 g, 0.359 mmol) and isovaleraldehyde (0.079 mL, 0.72 mmol). Purification by flash column chromatography on silica (4:1 hexanes:ethyl acetate) gave *Z*-**4** (0.115 g, 88%) as an oil and a 1.2:1 mixture of diastereomers. IR (ATR) 3323, 2972, 2954, 1643, 1505, 1467, 1448, 1330, 1301, 1260, 1115, 1078, 981, 954, 802, 754 cm^–1^. ^1^H NMR (500 MHz, CDCl_3_) δ = 7.85–7.81 (m, 4H), 7.65–7.61 (m, 2H), 7.55–7.50 (m, 4H), 5.46 (d, *J* = 10.9 Hz, 1H), 4.90–4.81 (m, 3H), 4.62 (ddd, *J* = 9.5, 4.2, 1.3 Hz, 1H), 4.33 (ddd, *J* = 10.7, 8.4, 2.4 Hz, 1H), 3.86 (dd, *J* = 11.1, 8.4 Hz, 1H), 3.70 (dd, *J* = 10.8, 1.4 Hz, 1H), 2.26–2.17 (m, 1H), 2.20–1.88 (m, 2H), 1.86–1.73 (m, 4H), 1.70 (d, *J* = 1.4 Hz, 3H), 1.67–1.60 (m, 1H), 1.66 (d, *J* = 1.3 Hz, 3H), 1.64 (s, 3H), 1.63 (s, 3H), 1.52 (s, 3H), 1.51 (s, 3H), 1.40–1.24 (m, 2H), 1.22–1.14 (m, 1H), 1.05–0.92 (3H), 0.91 (d, *J* = 7.0 Hz, 6H), 0.90 (d, *J* = 6.5 Hz, 3H), 0.88 (d, *J* = 6.8 Hz, 3H). ^13^C{^1^H} NMR (126 MHz, CDCl_3_) δ 147.2, 145.6, 137.8, 137.7, 133.8, 133.7, 132.3, 132.2, 129.1, 129.0, 128.8, 123.2, 123.1, 114.7, 112.0, 70.6, 68.4, 67.7, 66.6, 43.60, 43.58, 32.1, 31.7, 25.8, 25.7, 25.64, 25.62, 24.3, 24.0, 23.9, 23.4, 23.3, 23.2, 21.8, 21.1, 17.7. HRMS (ESI+) calcd *m*/*z* for C_21_H_32_NaO_3_S [M + Na]^+^ 387.1970, found 387.1965.

### (*E*)-2,7,11-Trimethyldodeca-5,10-dien-4-ol (**5**-*anti*)

Prepared according to the general procedure **B** using *E*-**4** (0.129 g, 0.354 mmol). Purification by flash column chromatography on silica gave **5-***anti* (0.038 g, 48%) as an oil and a 6.6:1 mixture of diastereomers. IR (ATR) 3084, 3056, 2928, 1600, 1583, 1494, 1450, 1381, 1068, 1025, 907, 757, 696 cm^–1^. ^1^H NMR (CDCl_3_, 500 MHz) δ = 5.53 (ddd, *J* = 15.4, 7.4, 0.9 Hz, 1H), 5.41 (ddd, *J* = 15.4, 7.0, 1.0 Hz, 1H), 5.09 (ddq, *J* = 8.6, 5.7, 1.5 Hz, 1H), 4.12 (q, *J* = 7.2 Hz, 1H), 2.12 (p, *J* = 6.9 Hz, 1H), 1.95 (q, *J* = 7.7 Hz, 2H), 1.68 (d, *J* = 1.4 Hz, 3H), 1.59 (s, 3H), 1.46 (ddd, *J* = 13.3, 7.7, 6.6 Hz, 1H), 1.34–1.29 (m, 4H), 0.98 (d, *J* = 6.8 Hz, 3H),0.92 (t, *J* = 6.8 Hz, 6H). ^13^C{^1^H} NMR (CDCl_3_, 126 MHz) δ = 137.5, 131.7, 131.3, 124.6, 71.4, 46.5, 36.9, 35.8, 25.74, 25.70, 24.6, 22.9, 22.5, 20.4, 17.7. HRMS (ESI+) calcd *m*/*z* for C_15_H_28_NaO [M + Na]^+^ 247.2038, found 247.2032.

### (*E*)-2,7,11-Trimethyldodeca-5,10-dien-4-ol (**5**-*syn*)

Prepared according to the general procedure **B** using *Z*-**4** (0.115 g, 0.315 mmol). Purification by flash column chromatography on silica gave **5-***syn* (0.030 g, 42%) as an oil and a 5.7:1 mixture of diastereomers. IR (ATR) 3083, 2978, 2927, 1610, 1580, 1495, 1445, 1381, 1068, 1025, 907, 757, 696 cm^–1^. ^1^H NMR (CDCl_3_, 500 MHz) δ = 5.50 (ddd, *J* = 15.4, 7.7, 0.8 Hz, 1H), 5.40 (ddd, *J* = 15.4, 7.2, 0.9 Hz, 1H), 5.09 (ddq, *J* = 8.5, 5.7, 1.4 Hz, 1H), 4.12 (q, *J* = 7.0 Hz, 1H), 2.11 (p, *J* = 6.9 Hz, 1H), 1.98–1.89 (m, 2H), 1.68 (d, *J* = 1.4 Hz, 3H), 1.59 (s, 3H), 1.46 (ddd, *J* = 13.6, 7.5, 6.9 Hz, 1H), 1.35 (bs, 1H), 1.34–1.27 (m, 4H), 0.98 (d, *J* = 6.7 Hz, 3H), 0.91 (dd, *J* = 7.9, 6.6 Hz, 6H). ^13^C{^1^H} NMR (CDCl_3_, 126 MHz) δ = 137.8, 131.8, 131.3, 124.6, 71.6, 46.5, 37.0, 36.1, 25.8, 25.7, 24.6, 22.8, 22.6, 20.5, 17.6. HRMS (ESI+) calcd *m*/*z* for C_15_H_28_NaO [M + Na]^+^ 247.2038, found 247.2038.

### (2*R*,*E*)-6-Phenyl-4-(phenylsulfonyl)hept-5-en-2-ol (**6**)

Prepared according to the general procedure **A** using **1** (0.1 g, 0.37 mmol) and (*R*)-propylene oxide (32 mg, 0.56 mmol). Purification by flash column chromatography on silica (4:1 hexanes:EtOAc) gave **6** (64%) as an oil and a partially separable 1:1 mixture of diastereomers. IR (ATR) 3380, 2972, 2928, 1723, 1645, 1632, 1510, 1298, 1245, 987, 972 cm^–1^. ^1^H NMR (500 MHz, CDCl_3_) δ = 7.88–7.83 (m, 4H), 7.62–7.58 (m, 2H), 7.51–7.46 (m, 4H), 7.32–7.23 (m, 6H), 7.22–7.19 (m, 4H), 5.54 (dq, *J* = 10.8, 1.5 Hz, 1H), 5.49 (dq, *J* = 10.5, 1.4 Hz, 1H), 4.35 (td, *J* = 11.0, 2.9 Hz, 1H), 4.19 (ddd, *J* = 10.6, 8.2, 5.4 Hz, 1H), 4.15–4.10 (m, 1H), 3.75 (dqd, *J* = 10.5, 6.2, 2.5 Hz, 1H), 2.44 (dt, *J* = 14.0, 5.4 Hz, 1H), 2.34 (ddd, *J* = 13.6, 10.6, 2.8 Hz, 1H), 2.25 (bs, 1H), 1.96 (ddd, *J* = 14.0, 8.2, 7.2 Hz, 1H), 1.89 (ddd, *J* = 13.7, 11.2, 2.5 Hz, 1H), 1.57 (d, *J* = 1.4 Hz, 3H), 1.53 (d, *J* = 1.4 Hz, 3H), 1.26 (d, *J* = 6.2 Hz, 3H), 1.21 (d, *J* = 6.2 Hz, 3H). *Spectral data for one diastereomer*: ^13^C{^1^H} NMR (CDCl_3_, 126 MHz) δ = 142.9, 142.0, 137.5, 133.6, 129.1, 128.8, 128.3, 127.8, 125.7, 120.4, 65.5, 62.5, 37.3, 23.0, 16.0. HRMS (ESI+) calcd *m*/*z* for C_19_H_23_O_3_S [M + H]^+^ 331.1368, found 331.1367.

### (1*S*,*E*)-1,5-Diphenyl-3-(phenylsulfonyl)hex-4-en-1-ol (**7**)

Prepared according to the general procedure **A** using **1** (0.200 g, 0.734 mmol) and (*R*)-styrene oxide (0.101 mL, 0.881 mmol). Purification by flash column chromatography on silica (4:1 hexanes:EtOAc) gave **7** (0.247 g, 86%) as an oil and a partially separable 1:1 mixture of diastereomers. IR (ATR) 3353, 3057, 3025, 2957, 2926, 1611, 1511, 1452, 1300, 1245, 1087, 1035, 819, 757, 698 cm^–1^. ^1^H NMR (CDCl_3_, 500 MHz) δ = 1H NMR (500 MHz, CDCl3) δ = 7.89–7.82 (m, 2H), 7.81–7.79 (m, 2H), 7.63–7.57 (m, 2H), 7.53–7.45 (m, 4H), 7.38–7.27 (m, 16H), 7.24–7.19 (m, 4H), 5.58 (dq, *J* = 10.5, 1.4 Hz, 1H), 5.49 (dq, *J* = 10.5, 1.4 Hz, 1H), 4.92 (t, *J* = 7.0 Hz, 1H), 4.67 (dd, *J* = 10.7, 2.9 Hz, 1H), 4.43 (td, *J* = 10.8, 3.0 Hz, 1H), 3.87 (ddd, *J* = 10.4, 9.0, 4.7 Hz, 1H), 2.74 (ddd, *J* = 13.7, 7.5, 4.8 Hz, 2H), 2.33 (ddd, *J* = 13.8, 9.0, 6.5 Hz, 2H), 1.60 (d, *J* = 1.4 Hz, 3H), 1.35 (d, *J* = 1.4 Hz, 3H). *Spectral data for one diastereomer*: ^13^C{^1^H} NMR (CDCl_3_, 126 MHz) δ = 143.7, 142.5, 142.1, 137.6, 133.6, 129.2, 128.9, 128.8, 128.4, 128.2, 127.9, 126.1, 125.8, 119.9, 72.0, 62.3, 37.1, 16.2. HRMS (ESI+) calcd *m*/*z* for C_24_H_24_NaO_3_S [M + Na]^+^ 415.1344, found 415.1345.

### (*E*)-6-Phenyl-4-(phenylsulfonyl)-1-((triisopropylsilyl)oxy)hept-5-en-2-ol (**8**)

Prepared according to general procedure **A** using **1** (0.222 g, 0.815 mmol) and TIPS-protected glycidol^23^ (0.225 g, 0.977 mmol). Purification by flash column chromatography on silica (4:1 hexanes:EtOAc) gave **8** (0.315 g, 77%) as an oil and a partially separable 1:1 mixture of diastereomers. IR (ATR) 3320, 2998, 2945, 1610, 1511, 1436, 11420, 1337, 1299, 1248, 1120, 1105, 1088, 972, 964, 812, 754 cm^–1^. ^1^H NMR (CDCl_3_, 500 MHz) δ = 7.91–7.85 (m, 4H), 7.65–7.58 (m, 2H), 7.53–7.48 (m, 4H), 7.34–7.21 (m, 10H), 5.54 (dq, *J* = 10.5, 1.4 Hz, 1H), 5.51 (dq, *J* = 10.7, 1.4 Hz, 1H), 4.36 (ddd, *J* = 11.6, 10.6, 2.8 Hz, 1H), 4.21 (ddd, *J* = 10.5, 8.0, 5.3 Hz, 1H), 3.99 (tdd, *J* = 7.2, 5.2, 3.6 Hz, 1H), 3.76–3.71 (m, 2H), 3.63 (ddd, *J* = 11.7, 5.9, 2.7 Hz, 1H), 3.58–3.53 (ddd, *J* = 9.8, 8.1, 6.7 Hz, 2H), 2.60 (bs, 1H), 2.50 (dt, *J* = 14.2, 5.3 Hz, 1H), 2.39 (ddd, *J* = 13.6, 10.8, 2.8 Hz, 1H), 1.93 (dt, *J* = 14.3, 7.8 Hz, 1H), 1.82 (ddd, *J* = 13.7, 11.6, 2.3 Hz, 1H), 1.62 (d, *J* = 1.4 Hz, 3H), 1.56 (d, *J* = 1.4 Hz, 3H), 1.14–1.07 (m, 6H), 1.06–1.02 (m, *J* = 6.9, 4.3, 2.2 Hz, 36H). *Spectral data for one diastereomer*: ^13^C{^1^H} NMR (CDCl_3_, 126 MHz) δ = 142.7, 142.2, 137.8, 133.6, 129.3, 128.9, 128.3, 127.8, 125.8, 120.7, 69.9, 66.8, 62.2, 31.7, 17.9, 16.1, 11.8. HRMS (ESI+) calcd *m*/*z* for C_28_H_42_NaO_4_SSi [M + Na]^+^ 525.2471, found 525.2476.

### (2*R*,6*S*,*E*)-6-Phenylhept-4-en-2-ol (**9**)

Prepared according to the general procedure **B** using **6** (0.10 g, 0.30 mmol). Purification by flash column chromatography on silica (4:1 hexanes:EtOAc) gave **9** (0.048 g, 84%) as an oil and a 2.3:1 mixture of diastereomers. IR (ATR) 3347, 2958, 2925, 2871, 2857, 1606, 1457, 1371, 1258, 1150, 1123, 1060, 969, 730 cm^–1^. *Spectral data for the major diastereomer*: ^1^H NMR (CDCl_3_, 500 MHz) δ = 7.31 (m, *J* = 8.0, 6.8, 1.3 Hz, 2H), 7.24–7.17 (m, 3H), 5.75 (dddt, *J* = 15.3, 6.8, 2.5, 1.3 Hz, 1H), 5.48 (dddd, *J* = 15.7, 8.0, 6.8, 1.3 Hz, 1H), 3.81 (dtdd, *J* = 8.8, 7.4, 5.5, 4.3 Hz, 1H), 3.48 (p, *J* = 6.9 Hz, 1H), 2.26–2.19 (m, 1H), 2.18–2.10 (m, 1H), 1.65 (s, 1H), 1.37 (dd, *J* = 7.0, 1.9 Hz, 3H), 1.19 (dd, *J* = 6.2, 1.3 Hz, 3H). ^13^C{^1^H} NMR (CDCl_3_, 126 MHz) δ = 146.0, 139.0, 128.4, 127.1, 126.1, 124.7, 67.3, 42.5, 42.3, 22.7, 21.4. HRMS (ESI+) calcd *m*/*z* for C_13_H_18_NaO [M + Na]^+^ 213.1255, found 213.1249.

### (2*R*,6*S*,*E*)-6-Phenylhept-4-en-6-d-2-ol (**9**-*d*)

Prepared according to the general procedure **B** using **6** (0.050 g, 0.15 mmol) and D_2_O (0.32 mL, 16 mmol). Purification by flash column chromatography on silica (4:1 hexanes:EtOAc) gave **9-***d* (0.024 g, 84%) as an oil. *Spectral data for the major diastereomer*: ^1^H NMR (CDCl_3_, 500 MHz) δ = 7.32–7.28 (m, 2H), 7.22–7.18 (m, 3H), 5.74 (ddt, *J* = 15.3, 2.4, 1.3 Hz, 1H), 5.47 (dddd, *J* = 15.3, 7.5, 6.6, 0.8 Hz, 1H), 3.81–3.78 (m, 1H), 2.23 (dddd, *J* = 12.9, 6.6, 4.9, 1.4 Hz, 1H), 2.14 (dtt, *J* = 13.8, 7.6, 1.3 Hz, 1H), 1.71 (bs, 1H), 1.36 (bs, 3H), 1.19 (d, *J* = 6.2 Hz, 3H). ^13^C{^1^H} NMR (CDCl_3_, 126 MHz) δ = 145.9, 139.0, 128.4, 127.0, 126.1, 124.7, 67.3, 42.5, 42.4, 22.7, 21.3. HRMS (ESI+) calcd *m*/*z* for C13H17DNaO [M + Na]^+^ 214.1318, found 214.1313.

### (1*S*,5S,*E*)-1,5-Diphenylhex-3-en-1-ol (**10**)

Prepared according to general procedure **B** using **7** (0.10 g, 0.254 mmol). Purification by flash column chromatography on silica (4:1 hexanes:EtOAc) gave **10** (0.051 g, 80%) as an oil and a 4.4:1 mixture of diastereomers. IR (ATR) 3328, 3058, 3016, 2928, 1268, 1110, 932, 711 cm^–1^. *Spectral data for the major diastereomer:*^1^H NMR (CDCl_3_, 500 MHz) δ = 7.36–7.33 (m, 4H), 7.31–7.27 (m, 3H), 7.21–7.14 (m, 3H), 5.73 (ddt, *J* = 15.4, 6.7, 1.3 Hz, 1H), 5.51–5.39 (m, 1H), 4.73–4.70 (m, 1H), 3.45 (p, *J* = 7.0 Hz, 1H), 2.01 (bs, 1H), 2.54–2.43 (m, 2H), 1.34 (t, *J* = 6.7 Hz, 3H). ^13^C{^1^H} NMR (CDCl_3_, 126 MHz) δ = 145.9, 143.9, 139.4, 128.39, 128.35, 127.4, 127.1, 126.1, 125.9, 124.3, 73.7, 42.7, 42.3, 21.3. HRMS (ESI+) Calculated for C_18_H_20_NaO [M + Na]^+^ 275.1412, found 275.1414.

### (2*R*,6*R*,*E*)-6-Phenyl-1-((triisopropylsilyl)oxy)hept-4-en-2-ol (**11**)

Prepared according to general procedure **B** using **8** (0.120 g, 0.240 mmol). Purification by flash column chromatography on silica (4:1 hexanes:EtOAc) gave **11** (0.085 g, 98%) as an oil and a 2.2:1 mixture of diastereomers. IR (ATR) 3420, 2980, 2928, 1614, 1570, 1265, 1110, 932, 886, 711 cm^–1^. *Spectral data for the major diastereomer:*^1^H NMR (CDCl_3_, 500 MHz) δ = 7.31–7.27 (m, 2H), 7.21–7.17 (m, 3H), 5.73–5.67 (m, 1H), 5.53–5.46 (m, 1H), 3.73–3.67 (m, 2H), 3.55–3.50 (m, 1H), 3.46 (p, *J* = 7.0 Hz, 1H), 2.49 (bs, 1H), 2.27–2.18 (m, 2H), 1.35 (d, *J* = 7.0 Hz, 3H), 1.15–1.08 (m, 3H), 1.07–1.04 (m, 18H). ^13^C{^1^H} NMR (CDCl_3_, 126 MHz) δ = 146.0, 138.1, 128.4, 127.1, 126.0, 124.4, 71.6, 66.8, 42.3, 36.3, 21.3, 17.9, 11.9. HRMS (ESI+) calcd *m*/*z* for C_22_H_38_NaO_2_Si [M + Na]^+^ 385.2539, found 385.2534.

### (*R*)-2-Phenylpropanal (**12**)

To a solution of **9**, **10**, or **11** (0.04 g) in DCM (5 mL) at −78 °C, we bubbled O_3_ into the reaction mixture until the solution turned blue in color. The reaction was left to sit for 5 min and then nitrogen was bubbled though the reaction until the solution became colorless. The reaction was quenched with dimethyl sulfide (0.25 mL), warmed to room temperature, and stirred for 1 h. The reaction was diluted with brine (15 mL) and extracted with DCM (2 × 15 mL). The combined organic extracts were dried over MgSO_4_ and concentrated *in vacuo*. Purification by flash column chromatography on silica (10:1 to 4:1 hexanes:EtOAc) gave **12** as an oil and enriched in the (−)-enantiomer according to polarimetry ([α]_D_ (*c* 0.5, CHCl_3_) = −14.6° (from **9**), −23.4° (from **10**), and −12.2° (from **11**). NMR data for **12** matched that previously reported.^25^

### (*R*,*E*)-*tert*-Butyl((2-methyl-5-(phenylsulfonyl)pent-3-en-1-yl)oxy)diphenylsilane (**17**)

To a solution of aldehyde **16** (0.2 g, 0.61 mmol) in THF (3.2 mL) at 0 °C was added vinyl magnesium bromide (0.1 M, 0.72 mL) and the resulting mixture was stirred for 1 h. The reaction was quenched with aq. NH_4_Cl (15 mL) and extracted with MTBE (2 × 15 mL). The combined organic extracts were dried over MgSO_4_, filtered, and concentrated *in vacuo*. The resulting allylic alcohol^52^ was redissolved in THF (1 mL) and water (3 mL), and the solution was degassed by freezing in liquid nitrogen, placing the flask under vacuum, and then thawing under a nitrogen atmosphere. Phenyl sulfonic acid (100 mg, 0.7 mmol) was then added followed by Pd(PPh_3_)_4_ (35 mg, 0.03 mmol), and the mixture was stirred for 24 h. The reaction was diluted with EtOAc (15 mL) and washed with aq. NaHCO_3_ (15 mL). The organic phase was dried over MgSO_4_, filtered, and concentrated *in vacuo*. Purification by chromatography on silica (10:1 to 4:1 hexanes:EtOAc) gave **17** (0.237 g, 81%) as an oil. IR (ATR) 3054, 2998, 2872, 1637, 1584, 1494, 1467, 1446, 1371, 1297, 1240, 1146, 1082, 1049, 1025, 1001, 881, 849 cm^–1^. ^1^H NMR (500 MHz, CDCl_3_) δ 7.80 (dd, *J* = 8.1, 1.5 Hz, 2H), 7.68–7.60 (m, 4H), 7.54 (t, *J* = 7.5 Hz, 1H), 7.48–7.42 (m, 4H), 7.39 (dd, *J* = 7.8, 6.5 Hz, 4H), 5.43 (dd, *J* = 15.5, 8.2 Hz, 1H), 5.37 (dd, *J* = 15.4, 6.5 Hz, 1H), 3.73 (dd, *J* = 6.9, 2.7 Hz, 2H), 3.40 (dd, *J* = 9.9, 6.1 Hz, 1H), 3.34 (dd, *J* = 9.9, 6.9 Hz, 1H), 2.35 (hept, *J* = 6.6 Hz, 1H), 1.04 (s, 9H), 0.94 (d, *J* = 6.7 Hz, 3H). ^13^C{^1^H} NMR (126 MHz, CDCl_3_) δ 143.7, 138.0, 135.5, 133.6, 133.5, 129.61, 129.59, 128.8, 128.5, 127.6, 116.0, 67.8, 60.1, 39.3, 26.8, 19.2, 16.1. HRMS (ESI+) calcd *m*/*z* for C_28_H_34_NaO_3_SSi [M + Na]^+^ 501.1896, found 501.1893.

### (2*R*,7*R*,*E*)-8-((*tert*-Butyldiphenylsilyl)oxy)-1-(4-methoxyphenyl)-7-methyloct-4-en-2-ol (**15**)

To a solution of sulfone **17** (0.073 g, 0.15 mmol) in THF (0.76 mL) at −78 °C was added BuLi (2.5 M, 0.073 mL), and the resulting yellow solution was stirred for 10 min before warming to −20 °C for 20 min. After cooling the solution to −78 °C, epoxide **18** (0.037 g, 0.23 mmol) was added, and the mixture was slowly warmed to room temperature over 3 h followed by stirring at this temperature for 1 h. The reaction was quenched with aq. NH_4_Cl (15 mL) and extracted with EtOAc (2 × 15 mL). The combined organic extracts were dried over MgSO_4_, filtered, and concentrated *in vacuo*. The crude product was reduced directly with [Sm(H_2_O)_*n*_]I_2_ according to the general procedure **B**. Purification by column chromatography on silica (4:1 hexanes EtOAc) gave **15** (47 mg, 62%) as an oil. IR (ATR) 3062, 2978, 2856, 1642, 1637, 1515, 1484, 1467, 1446, 1371, 1297, 1240, 1146, 1082, 1049, 1025, 1001, 881, 849 cm^–1^. ^1^H NMR (500 MHz, CDCl_3_) δ 7.65 (dq, *J* = 7.5, 1.7 Hz, 4H), 7.47–7.32 (m, 6H), 7.17–7.08 (m, 2H), 6.89–6.82 (m, 2H), 5.54–5.46 (m, 1H), 5.43 (ddd, *J* = 15.3, 7.2, 6.0 Hz, 1H), 3.80 (s, 3H), 3.79–3.73 (m, 1H), 2.73 (dd, *J* = 13.6, 5.0 Hz, 1H), 2.63 (dd, *J* = 13.7, 7.8 Hz, 1H), 2.30–2.19 (m, 3H), 2.12 (dt, *J* = 13.4, 7.4 Hz, 1H), 1.98–1.86 (m, 1H), 1.79–1.67 (m, 1H), 1.62 (s, 1H), 1.05 (s, 9H), 0.90 (d, *J* = 6.7 Hz, 3H). ^13^C{^1^H} NMR (126 MHz, CDCl_3_) δ 158.2, 135.6, 134.01, 133.98, 132.6, 130.5, 130.4, 130.3, 129.5, 127.6, 127.3, 113.9, 72.1, 68.3, 55.3, 42.3, 40.0, 36.4, 36.0, 26.9, 19.3, 16.5, −0.0. HRMS (ESI+) calcd *m*/*z* for C_32_H_42_NaO_3_Si [M + Na]^+^ 525.2801, found 525.2801.

## Data Availability

The data underlying this study are available in the published article and its Supporting Information.

## Supporting Information Available

Copies of NMR spectra, results from changes to water equivalents for [Sm(H_2_O)_*n*_]I_2_ reductions of **2d** and **2e**, and evaluation of sulfone group stereochemistry in [Sm(H_2_O)_*n*_]I_2_ reductions of **2e** (PDF)
