# Supplementary material for: Hydroxyl-Directed Regio- and Diastereoselective Allylic Sulfone Reductions with [Sm(H2O)n]I2
Source: J Org Chem. 2023 Dec 13;89(1):692–700. doi: 10.1021/acs.joc.3c01647 (PMC10777405; doi:10.1021/acs.joc.3c01647)
Supplement: Supplementary file 1 — jo3c01647_si_001.pdf [file jo3c01647_si_001.pdf]

Supporting Information for

Hydroxyl-Directed Regio- and Diastereoselective Allylic Sulfone Reductions with $[Sm(H_2O)_n]I_2$

Cody L. Schwans, Trevor D. Clark, and Gregory W. O'Neil*
Department of Chemistry, Western Washington University, Bellingham, WA, 98225
*Email: oneilg@wwu.edu

Contents

Compound	^{1}H NMR spectrum	^{13}C NMR spectrum
**methyl 3-phenylbut-2-enoate**	S2	S2
**1**	S2	S2
**2a**	S3	S3
**2b**	S4	S4
**2c**	S5	S5
**2d**	S6	S6
**2e**	S7	S7
**2f**	S8	S8
**3a**	S9	S9
**3b**	S10	S10
**3c**	S11	S11
**3d**	S12	S12
**3e**	S13	S13
**3e-*d***	S14	S14
**3f**	S15	S15
***E*-4**	S16	S16
***Z*-4**	S17	S17
**5-*anti***	S18	S18
**5-*syn***	S19	S19
**6**	S20	S20
**7**	S21	S21
**8**	S22	S22
**9**	S23	S23
**9-*d***	S24	S24
**10**	S25	S25
**11**	S26	S26
**17**	S27	S27
**15**	S28	S28
Table S1 – Water equiv. optimization results for $[Sm(H_2O)_n]I_2$ reduction of **2c** and **2d**.		S29
Figure S1 – Evaluation of phenyl sulfone stereochemistry in $[Sm(H_2O)_n]I_2$ reduction of **2e**		S29

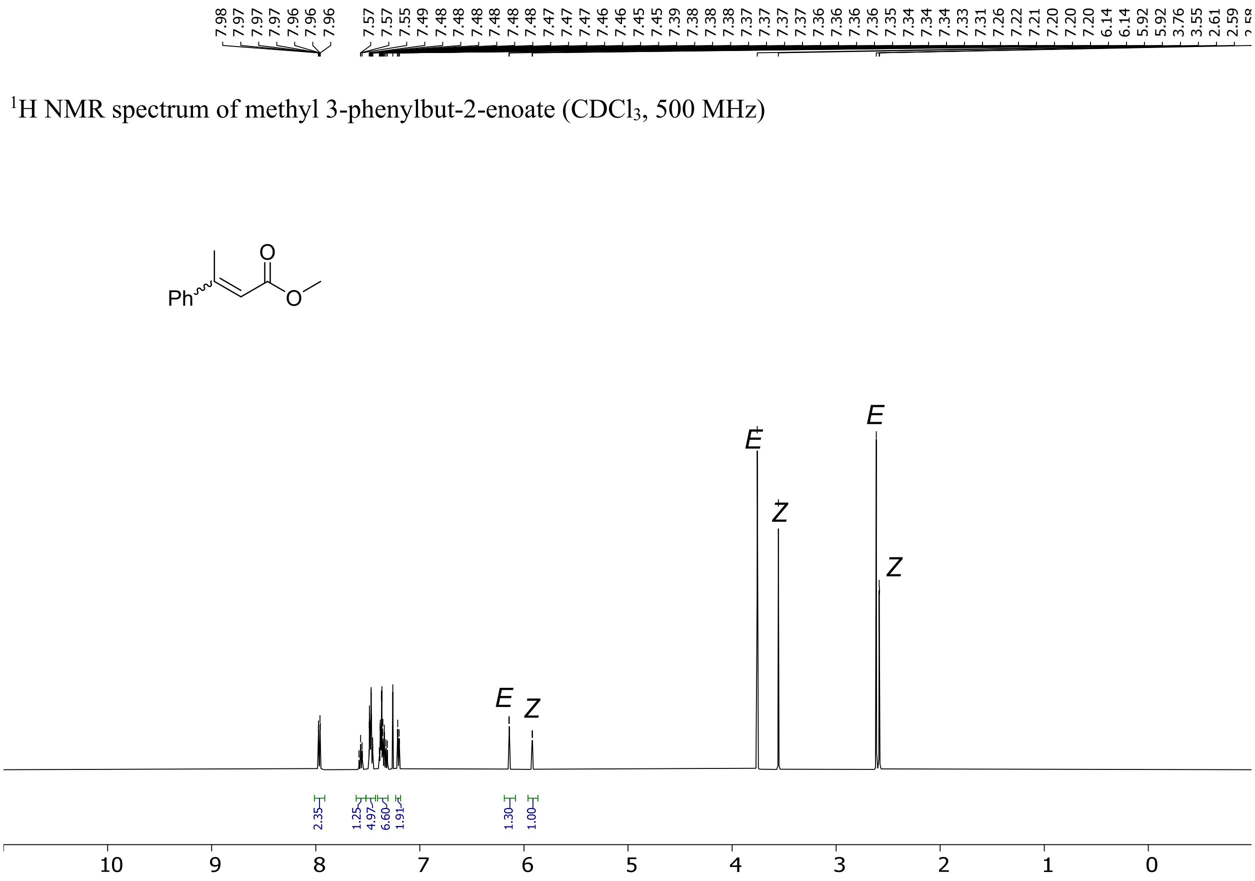

^{1}H NMR spectrum of methyl 3-phenylbut-2-enoate ($CDCl_3$, 500 MHz)

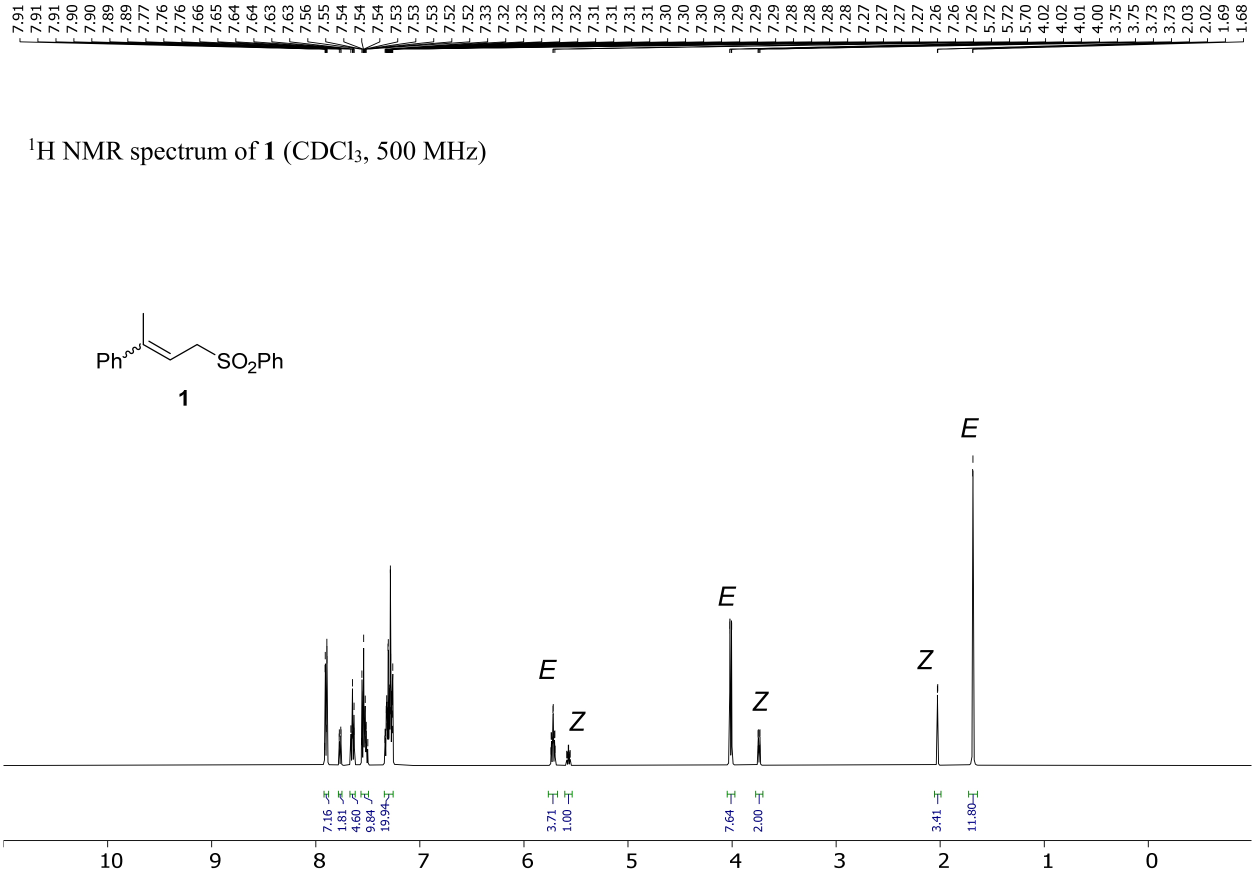

^{1}H NMR spectrum of **1** ($CDCl_3$, 500 MHz)

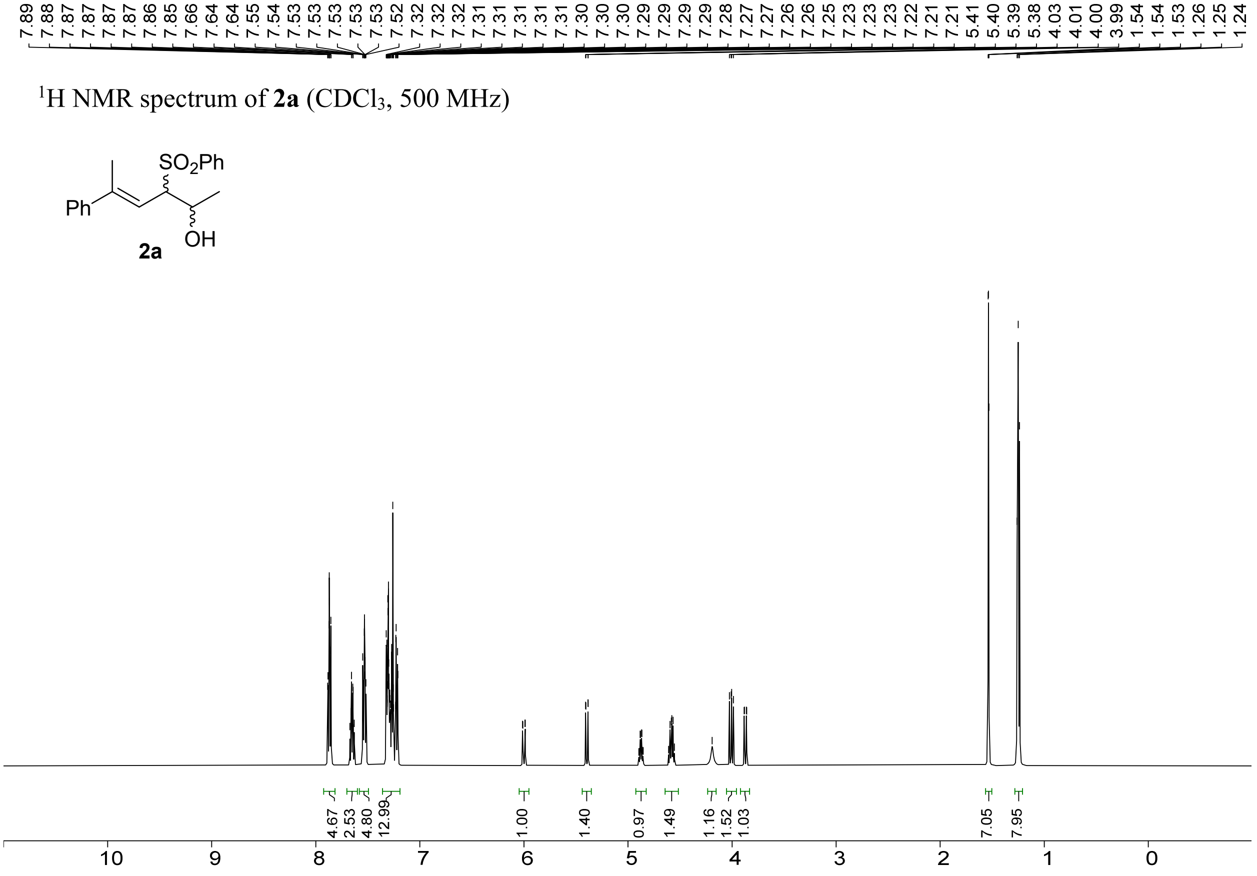

^{1}H NMR spectrum of **2a** ($CDCl_3$, 500 MHz)

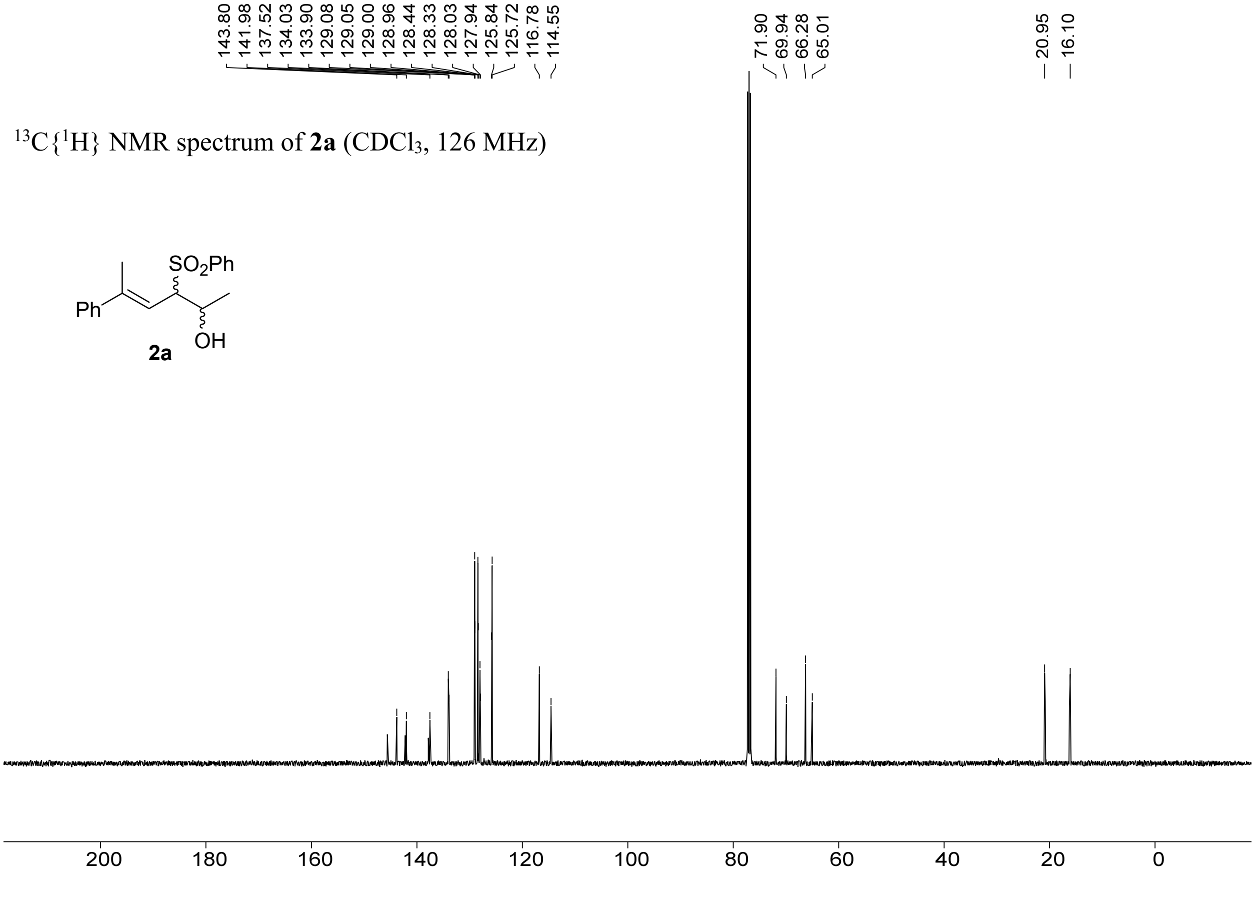

^{13}C{^{1}H} NMR spectrum of **2a** ($CDCl_3$, 126 MHz)

^{1}H NMR spectrum of **2b** ($CDCl_3$, 500 MHz)

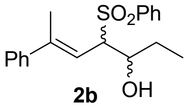

2b

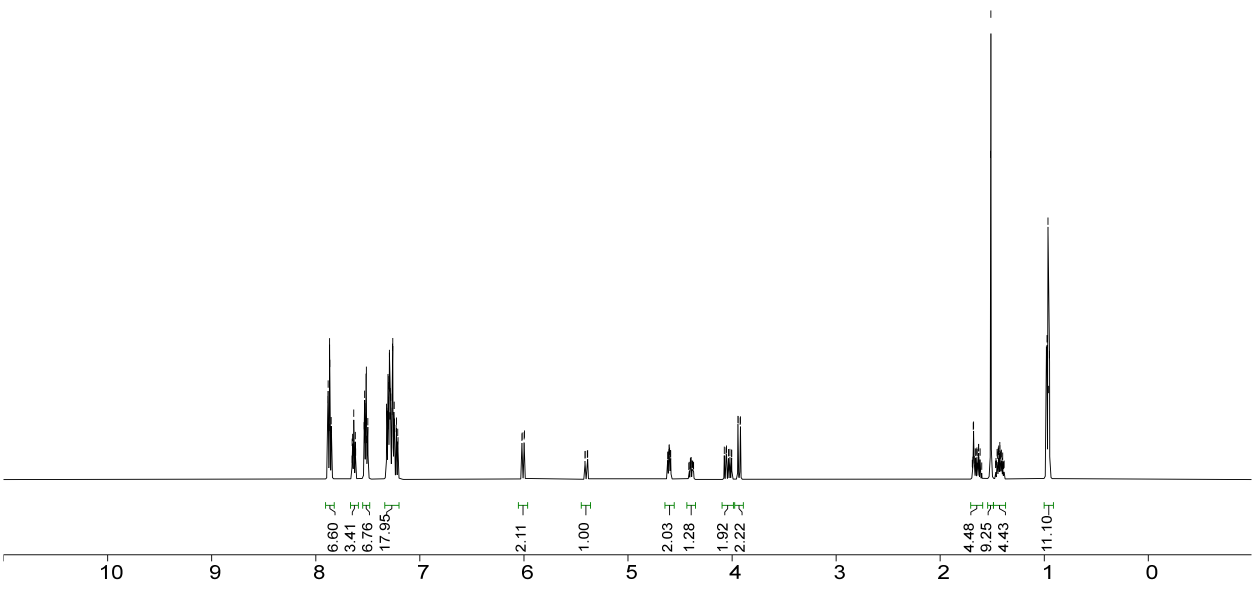

^{13}C{^{1}H} NMR spectrum of **2b** ($CDCl_3$, 126 MHz)

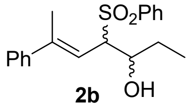

2b

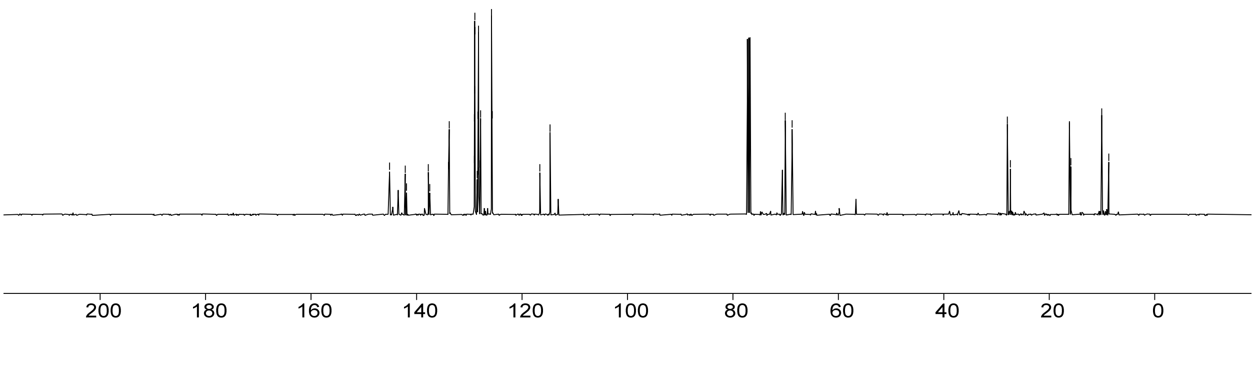

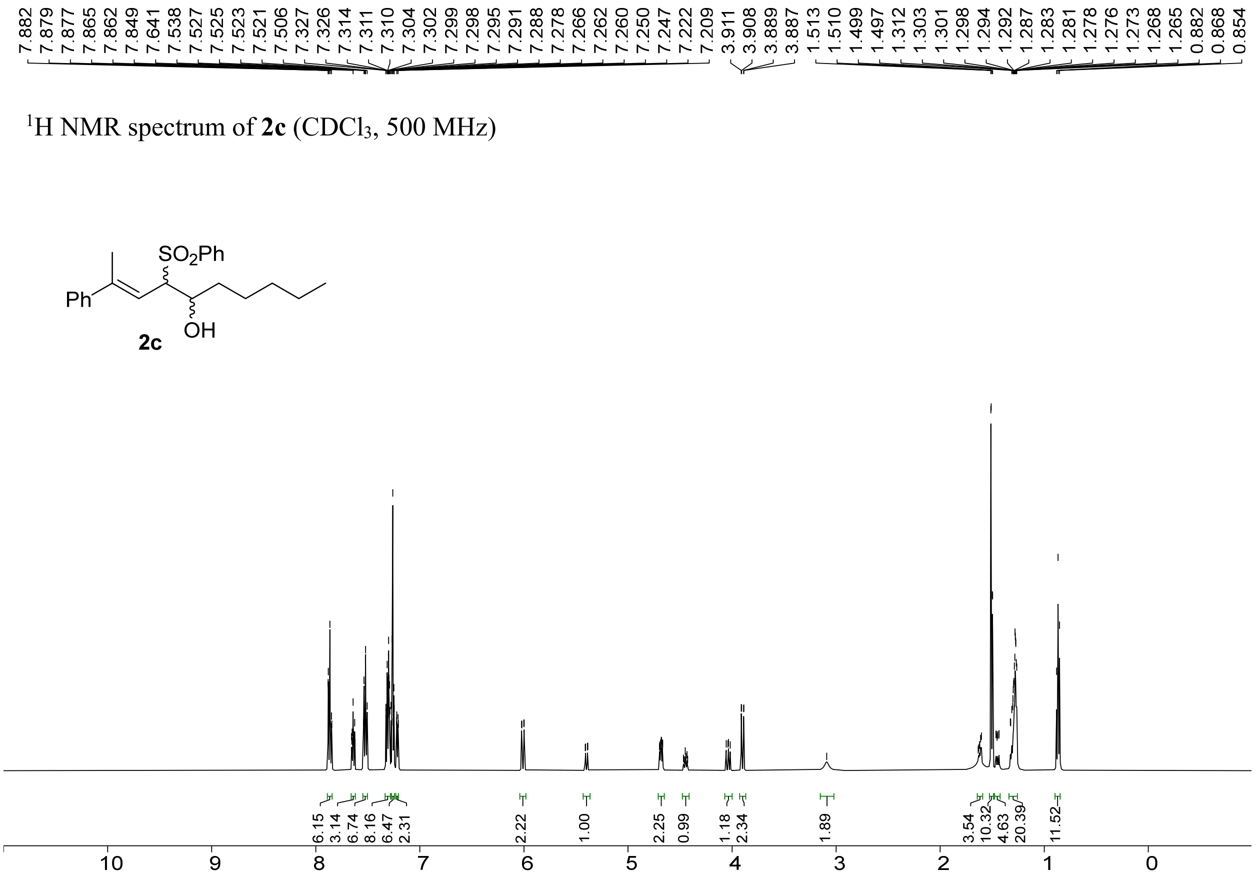

^{1}H NMR spectrum of **2c** ($CDCl_3$, 500 MHz)

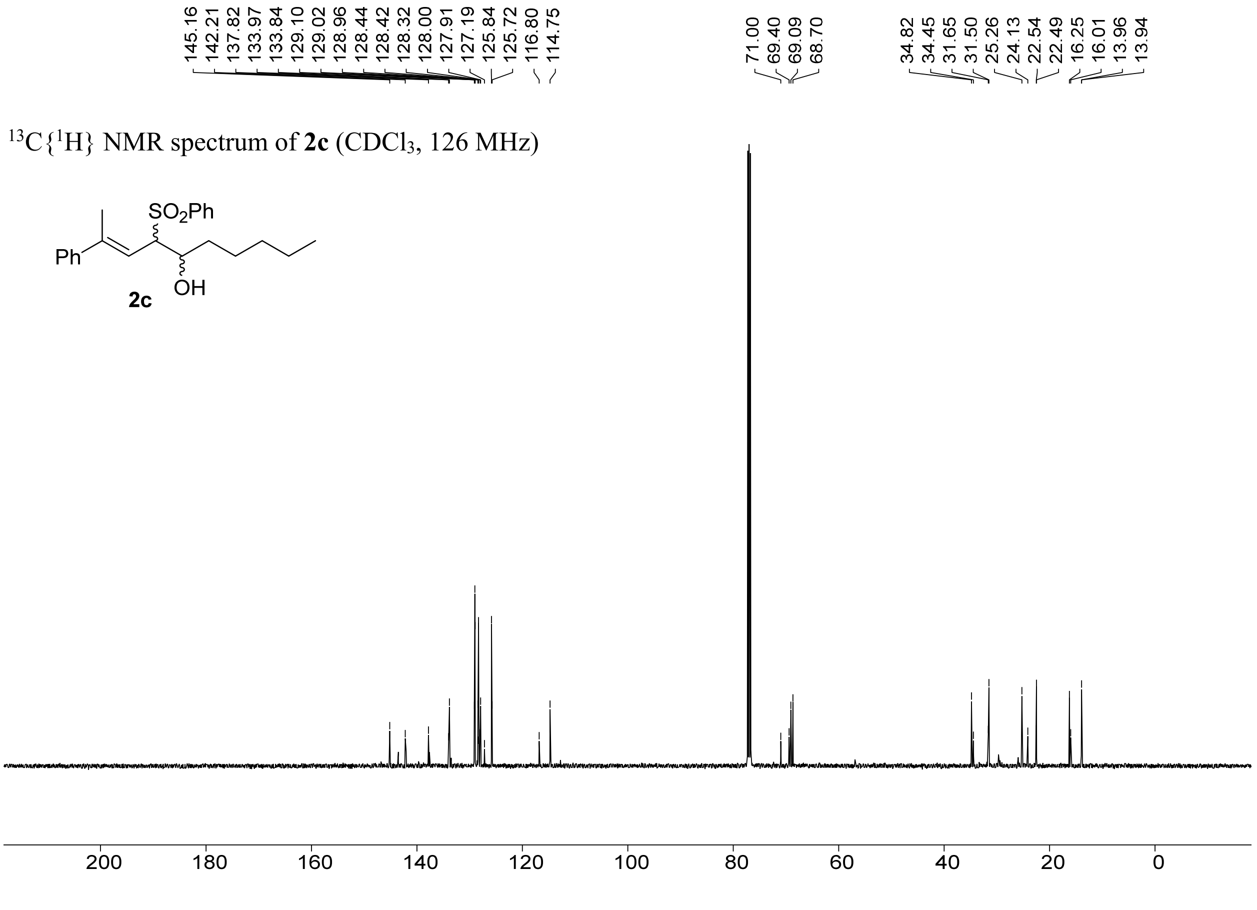

^{13}C{^{1}H} NMR spectrum of **2c** ($CDCl_3$, 126 MHz)

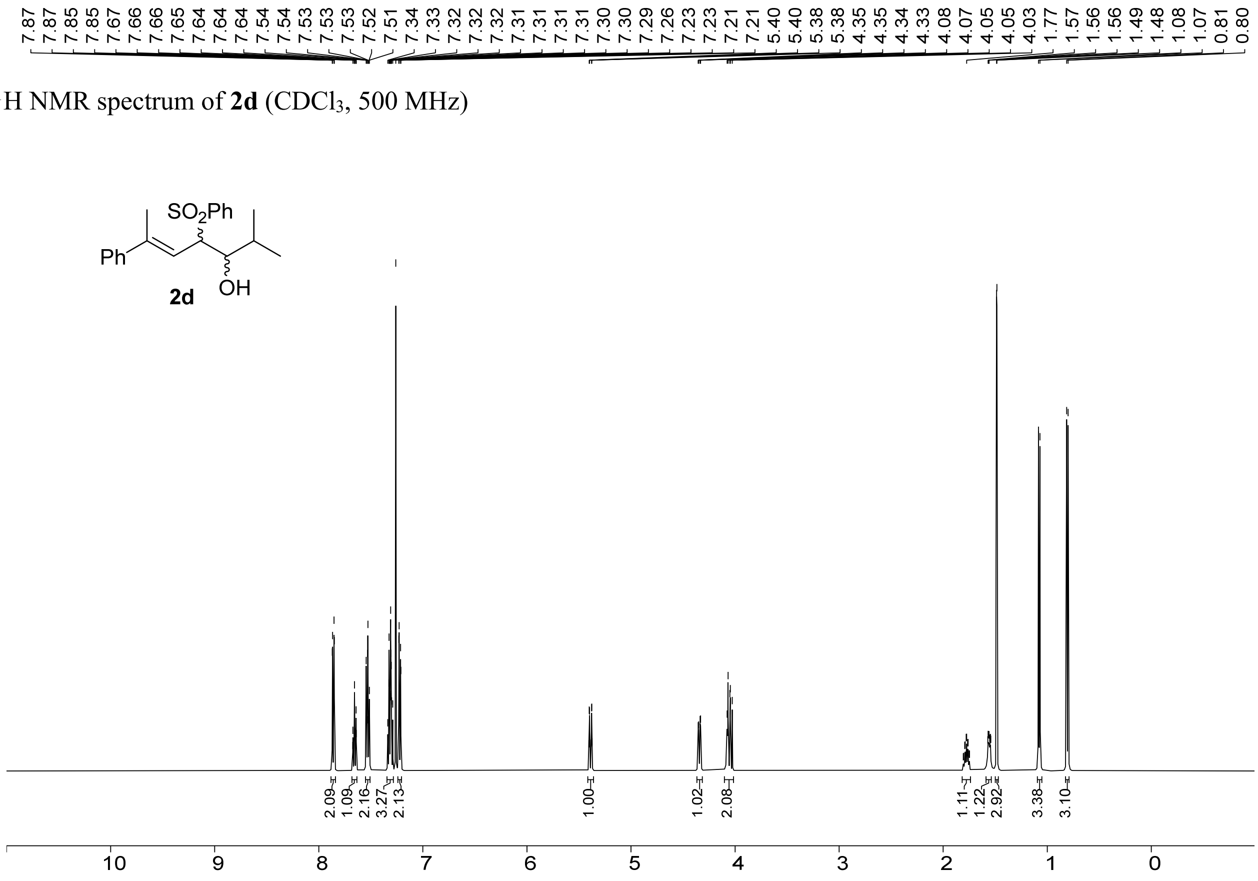

^{1}H NMR spectrum of **2d** ($CDCl_3$, 500 MHz)

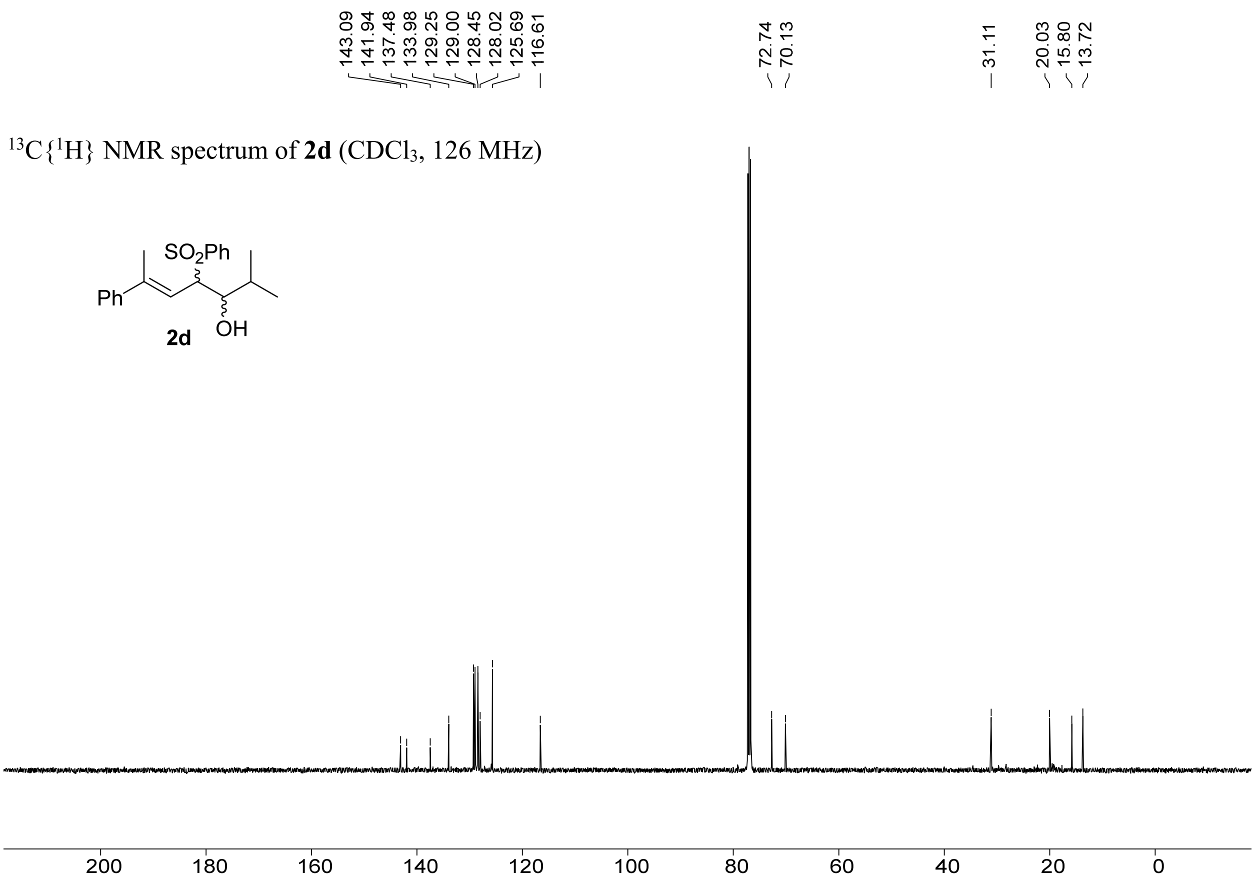

^{13}C{^{1}H} NMR spectrum of **2d** ($CDCl_3$, 126 MHz)

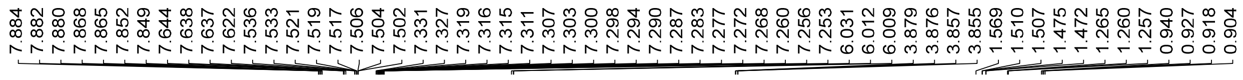

^{1}H NMR spectrum of **2e** ($CDCl_3$, 500 MHz)

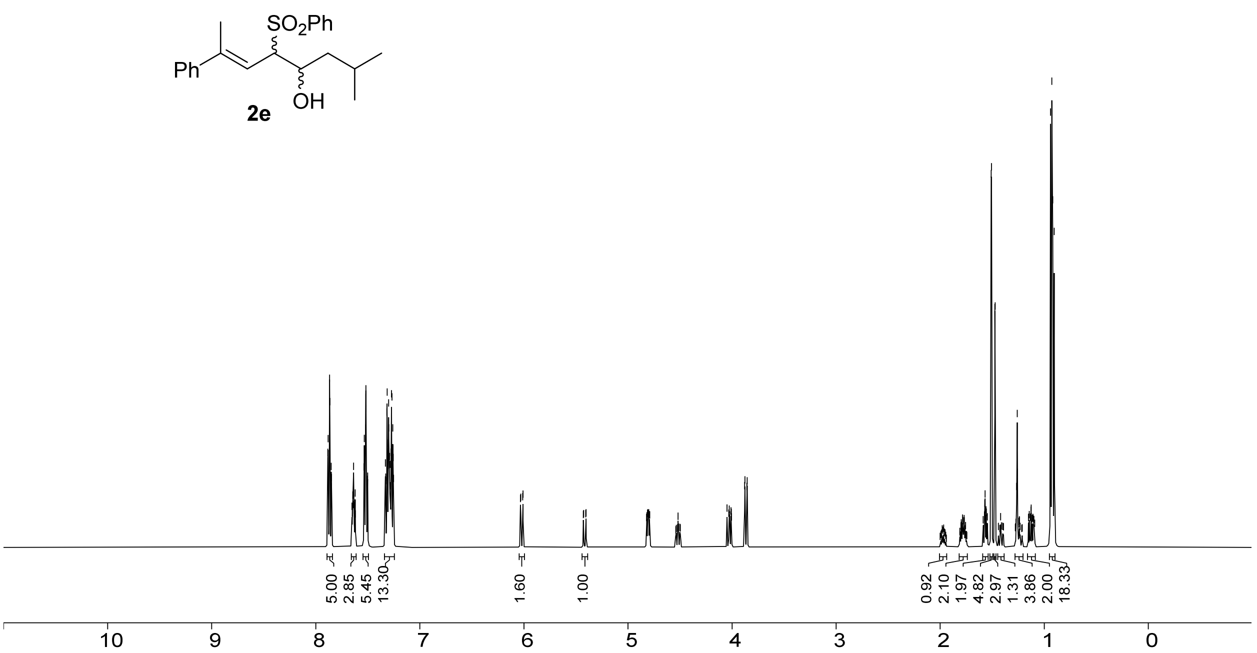

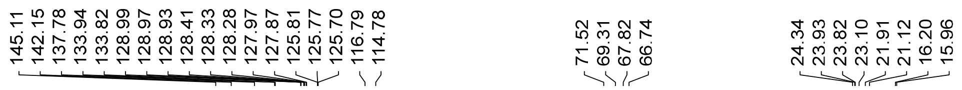

^{13}C{^{1}H} NMR spectrum of **2e** ($CDCl_3$, 126 MHz)

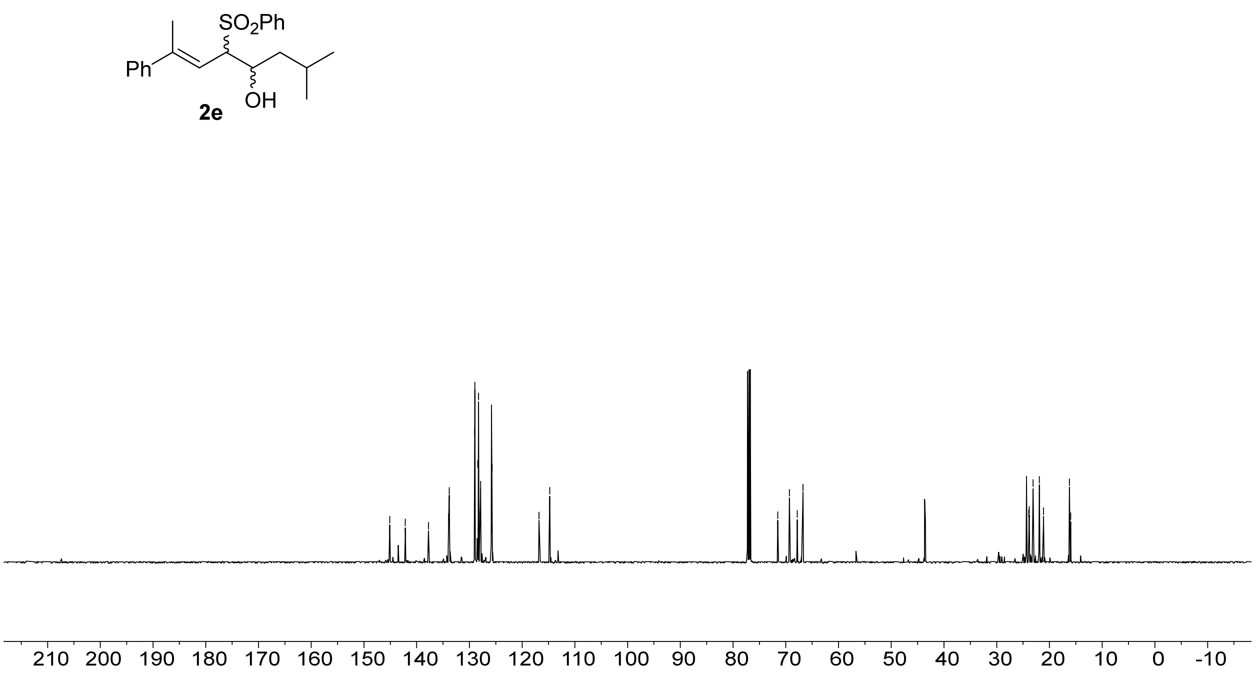

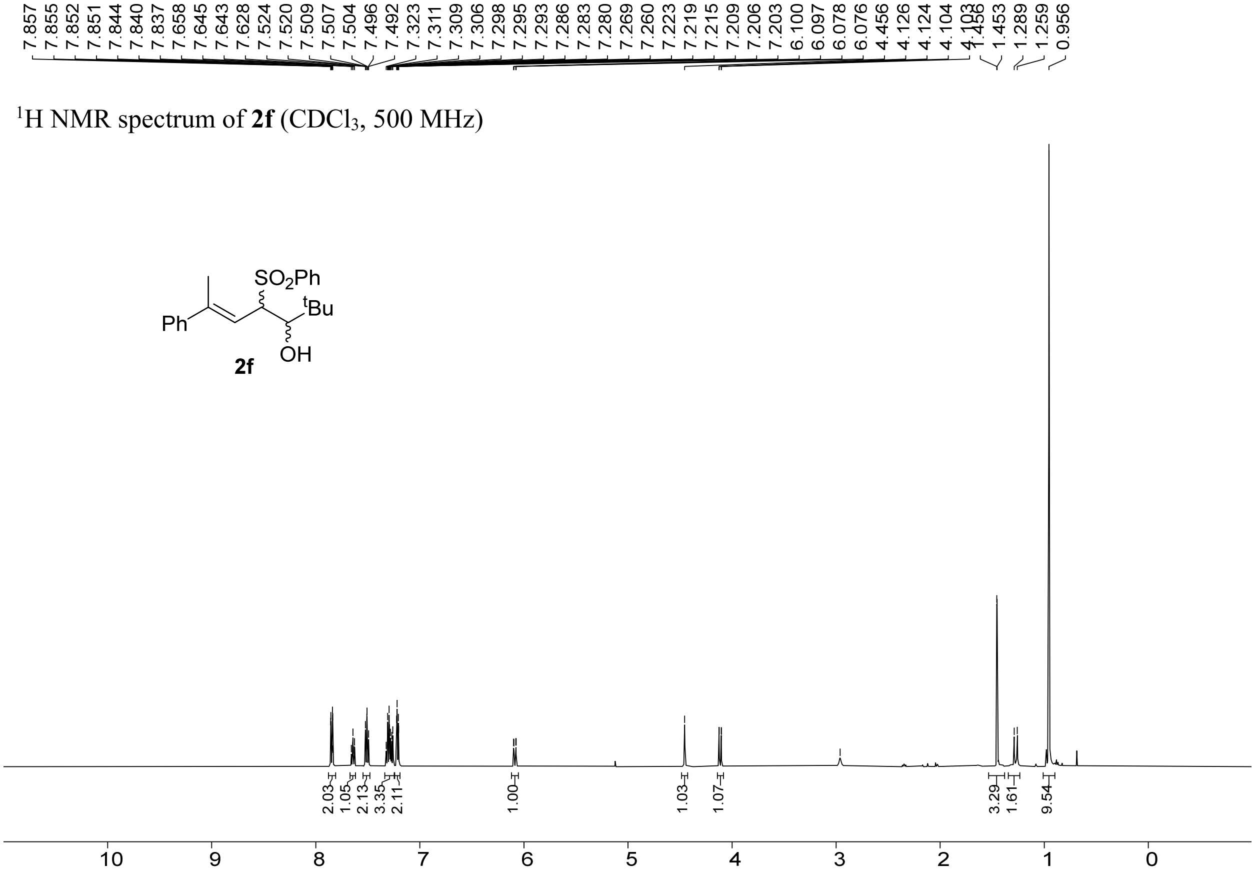

^{1}H NMR spectrum of **2f** ($CDCl_3$, 500 MHz)

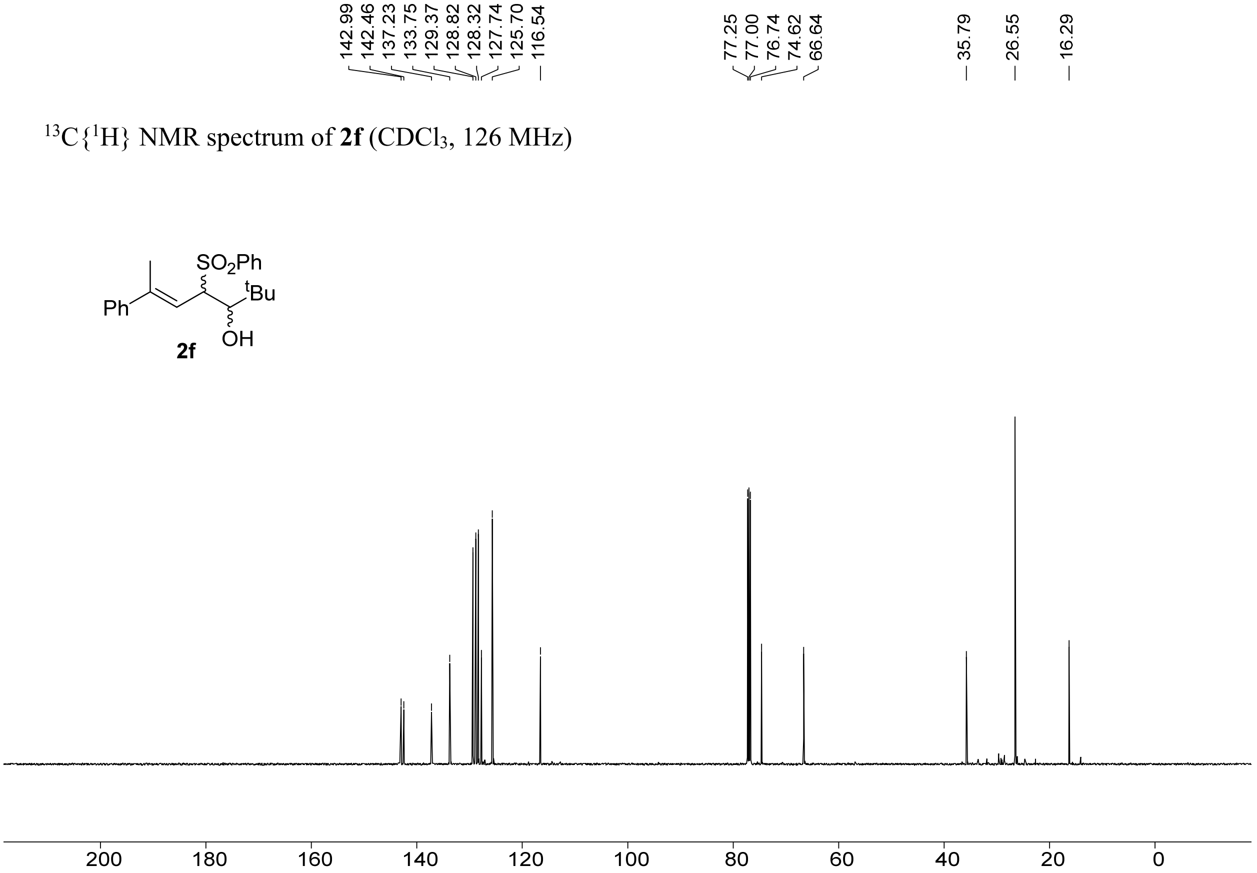

^{13}C{^{1}H} NMR spectrum of **2f** ($CDCl_3$, 126 MHz)

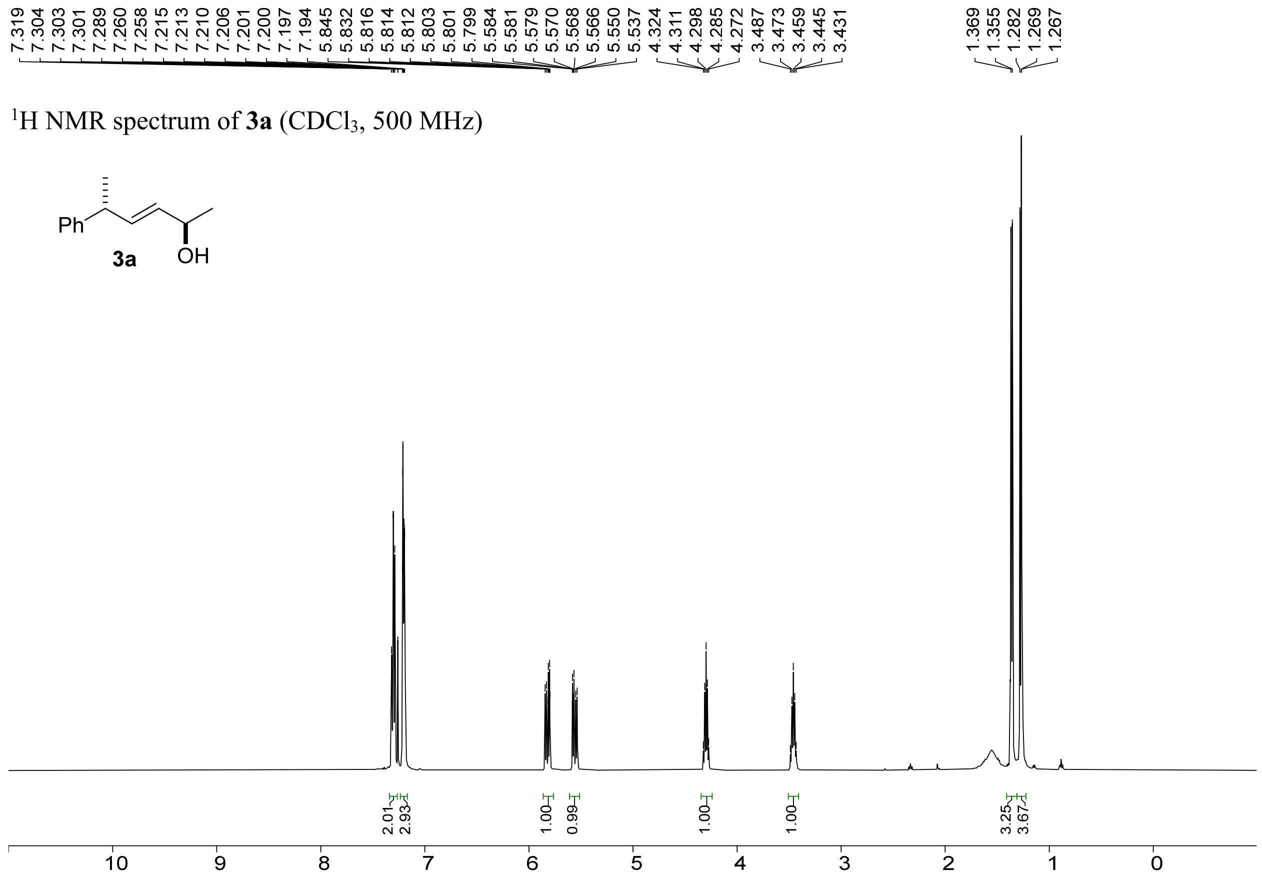

^{1}H NMR spectrum of **3a** ($CDCl_3$, 500 MHz)

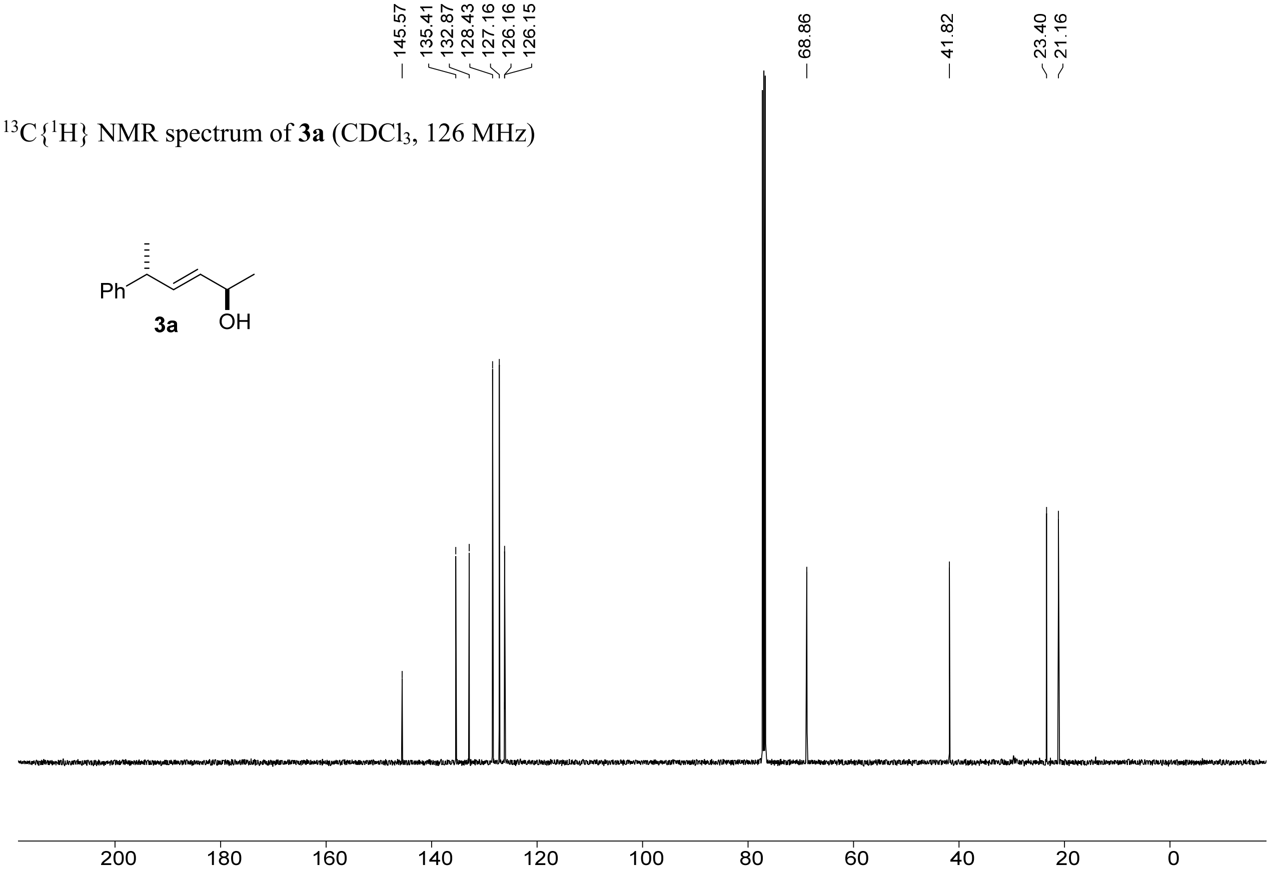

^{13}C{^{1}H} NMR spectrum of **3a** ($CDCl_3$, 126 MHz)

^{1}H NMR spectrum of **3b** ($CDCl_3$, 500 MHz)

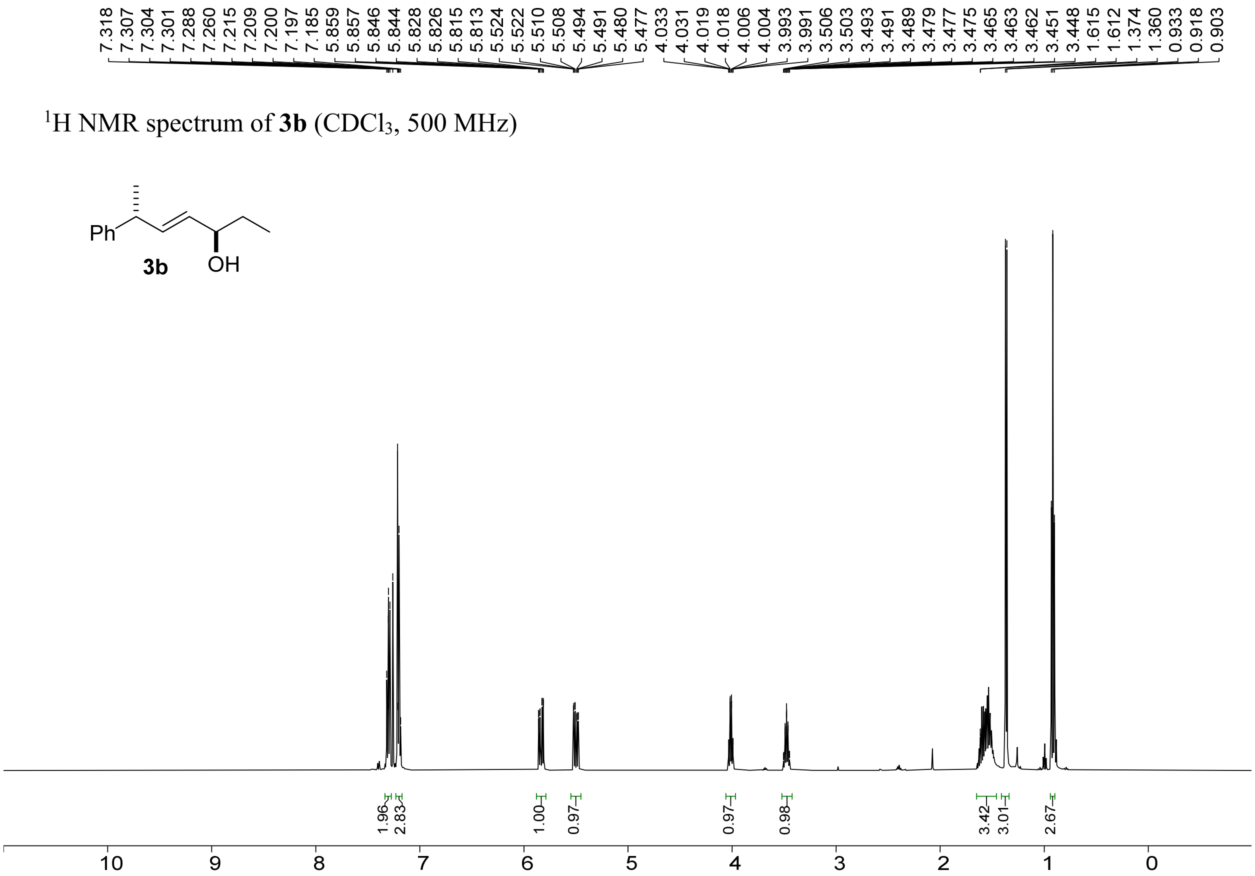

$^{13}C\{^{1}H\}$ NMR spectrum of **3b** ($CDCl_3$, 126 MHz)

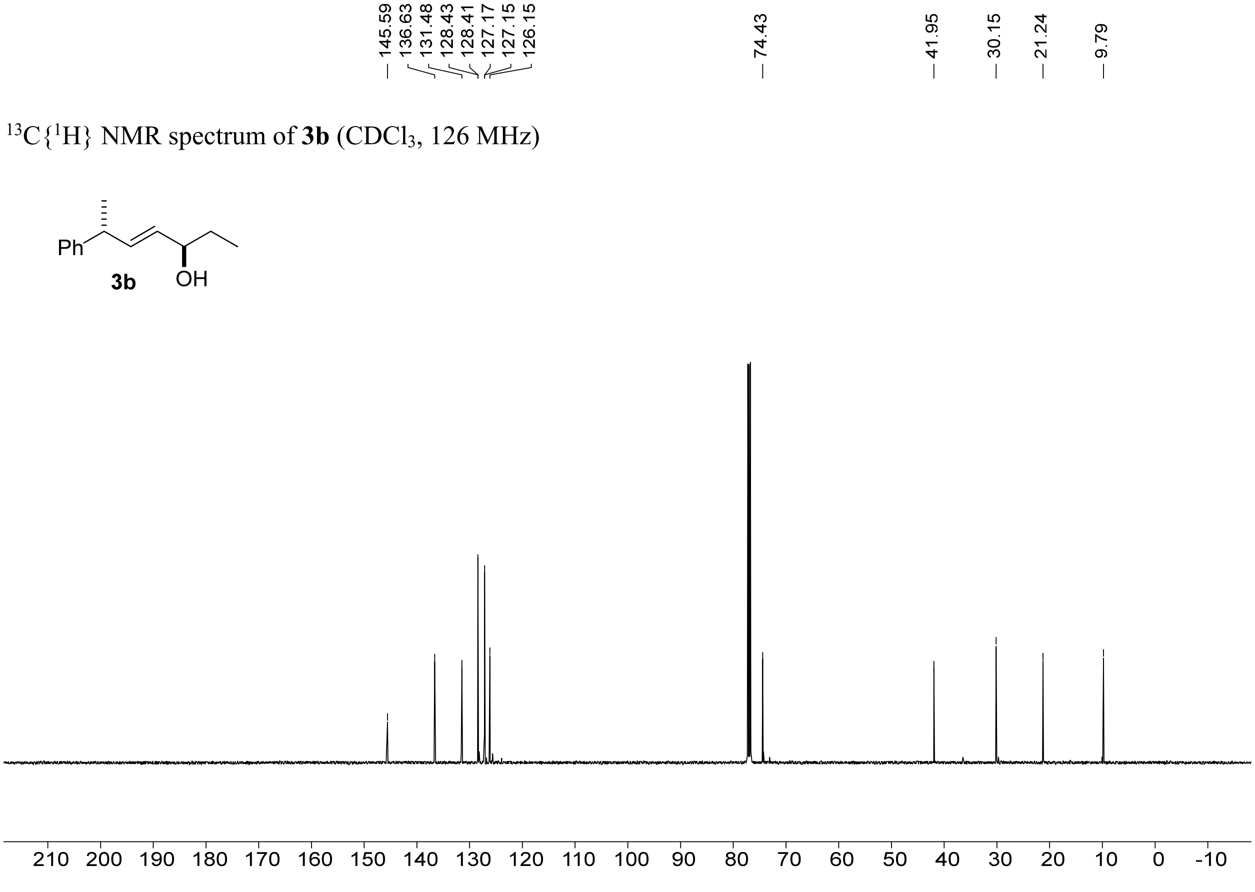

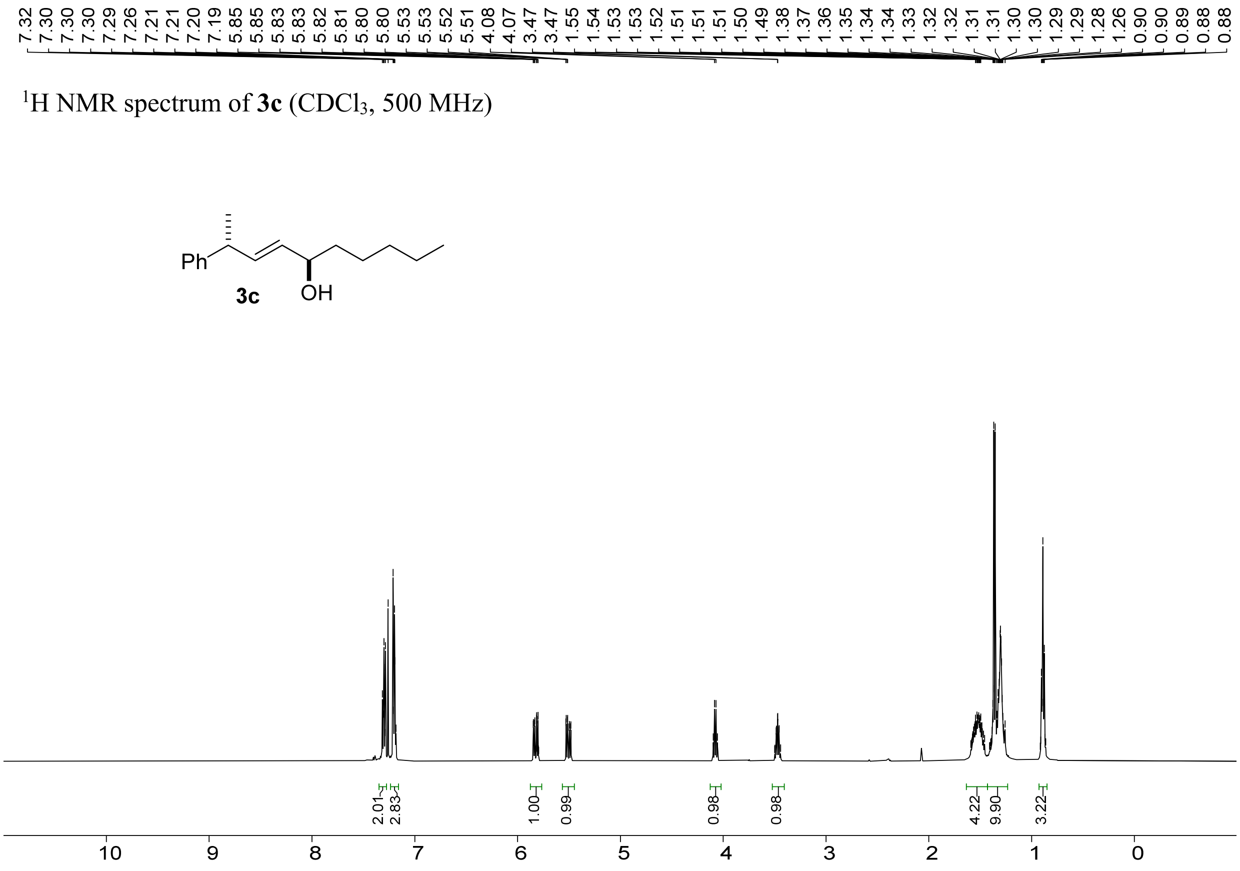

^{1}H NMR spectrum of **3c** ($CDCl_3$, 500 MHz)

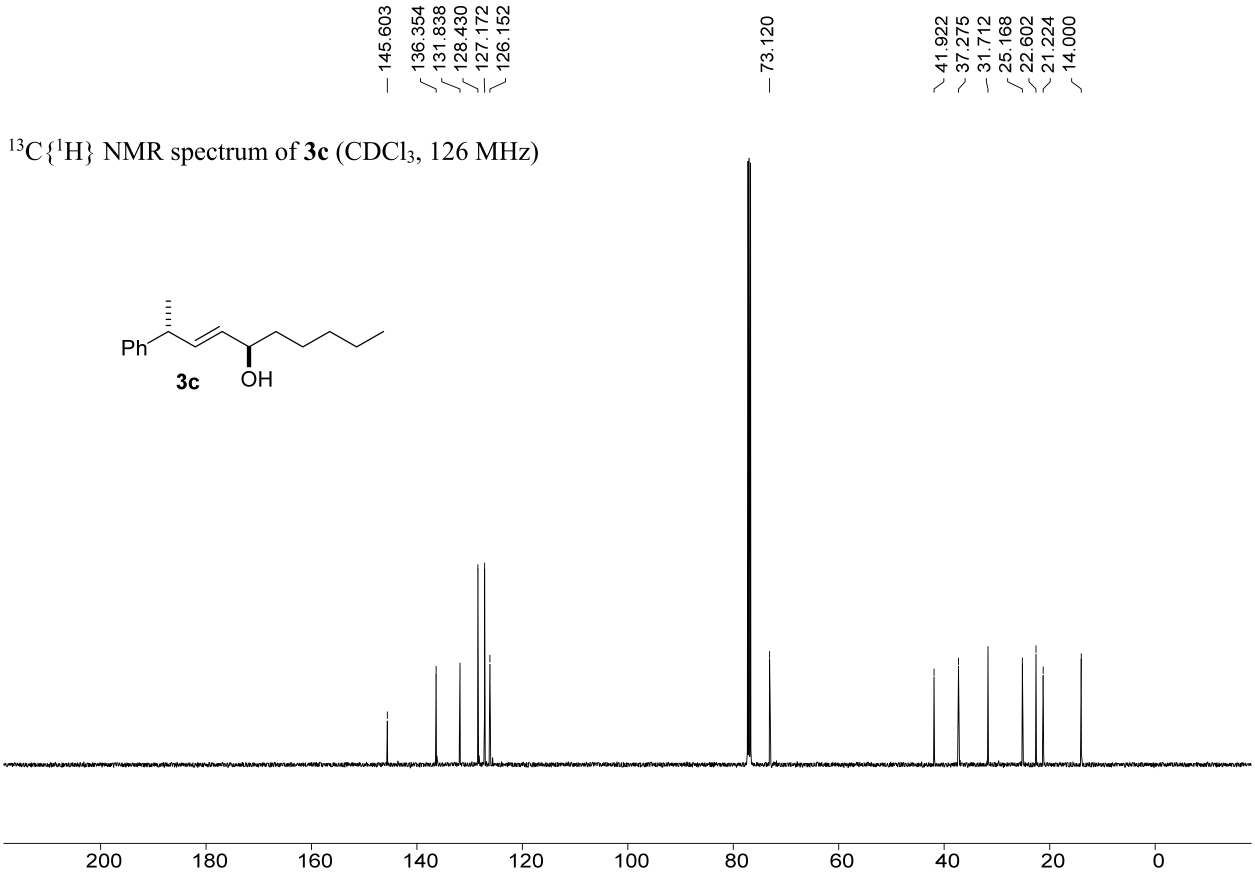

^{13}C{^{1}H} NMR spectrum of **3c** ($CDCl_3$, 126 MHz)

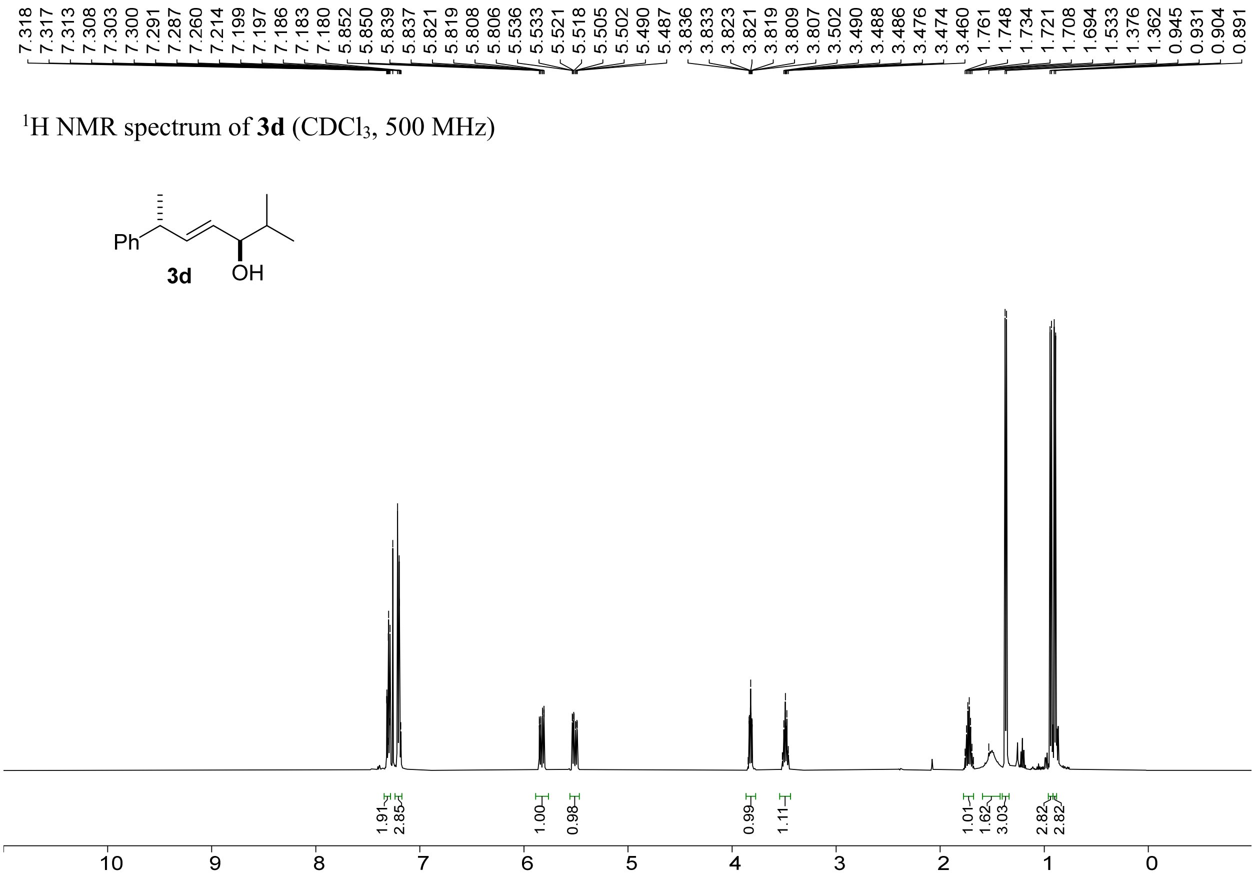

^{1}H NMR spectrum of **3d** ($CDCl_3$, 500 MHz)

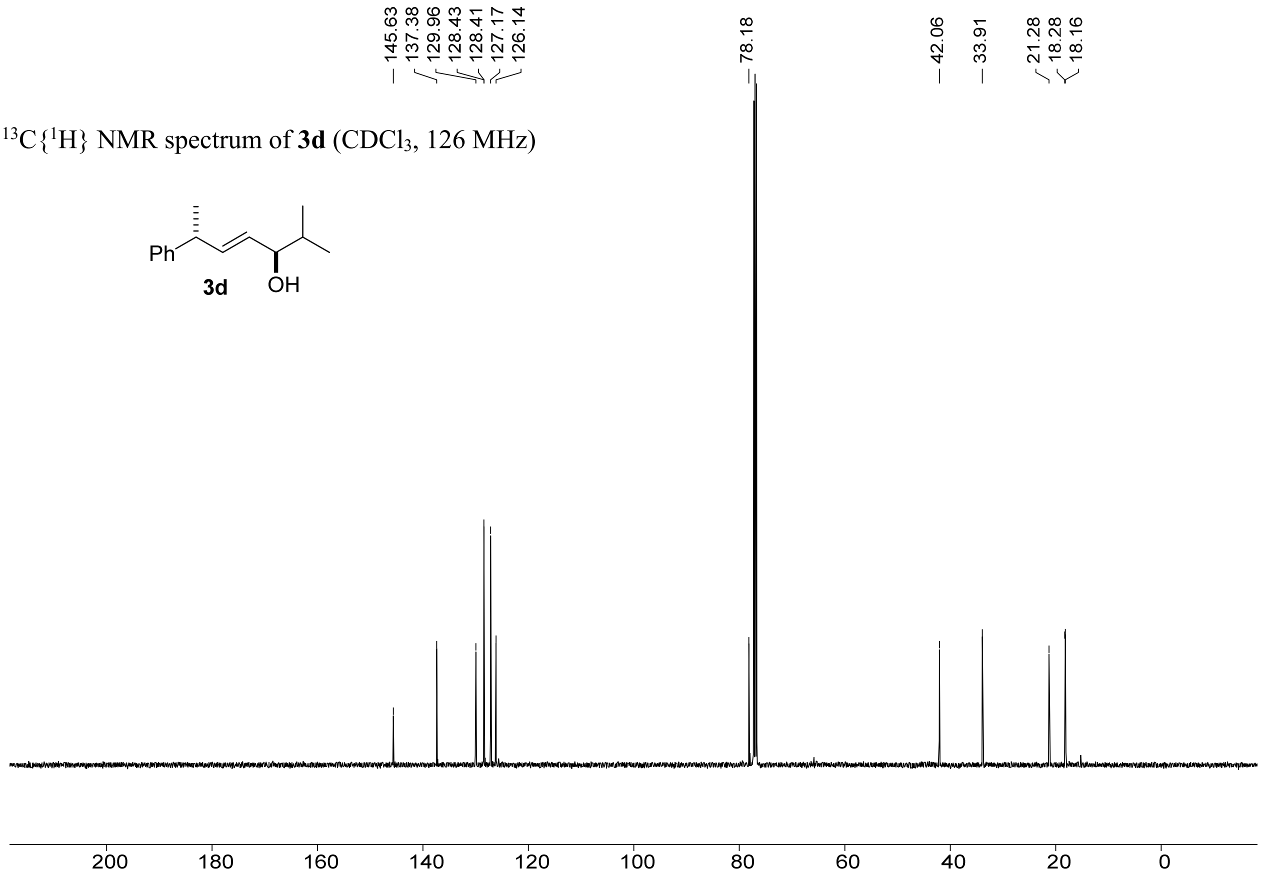

^{13}C{^{1}H} NMR spectrum of **3d** ($CDCl_3$, 126 MHz)

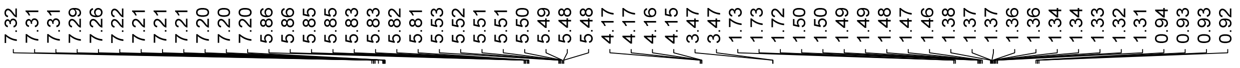

^{1}H NMR spectrum of **3e** ($CDCl_3$, 500 MHz)

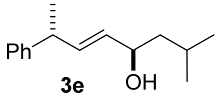

3e

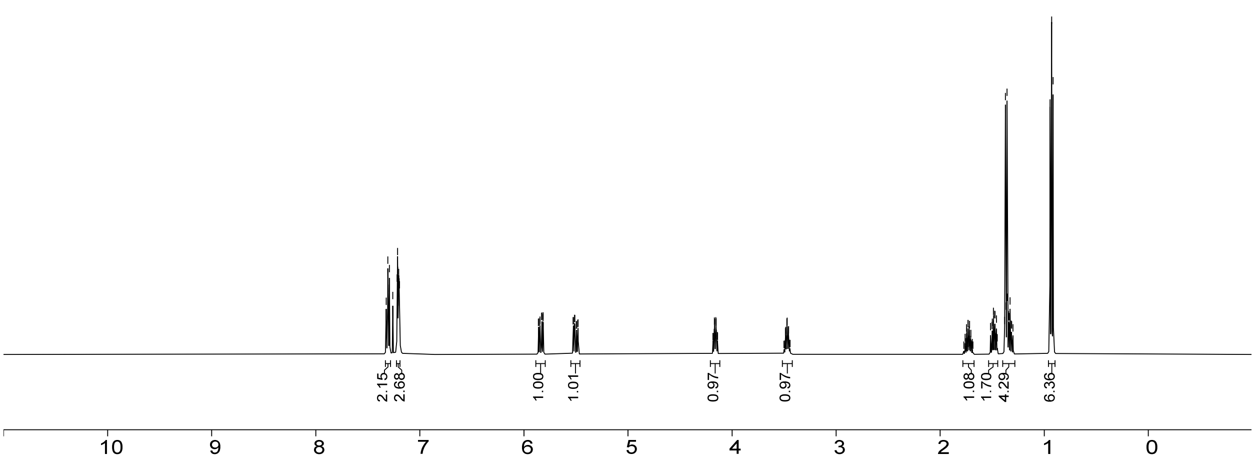

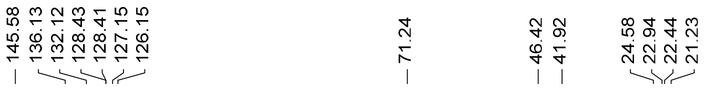

^{13}C{^{1}H} NMR spectrum of **3e** ($CDCl_3$, 126 MHz)

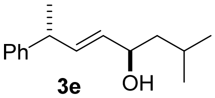

3e

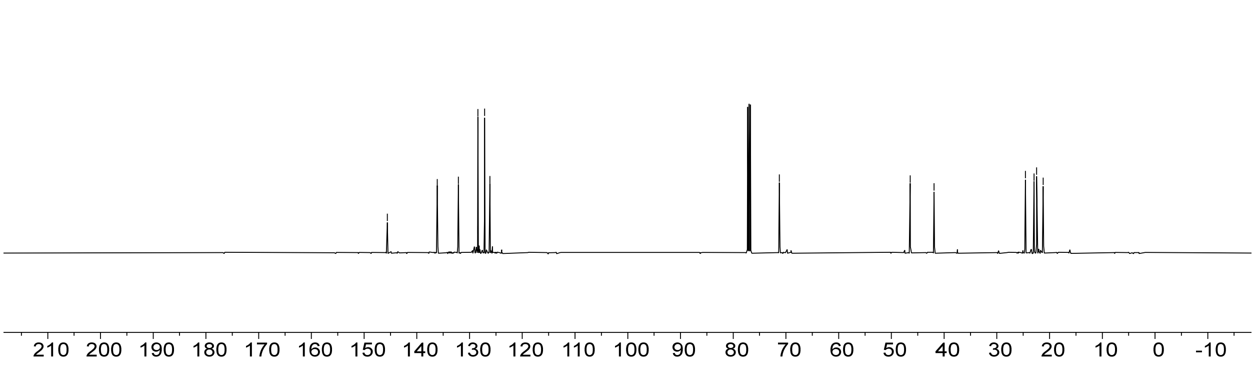

^{1}H NMR spectrum of **3e-*d*** ($CDCl_3$, 500 MHz)

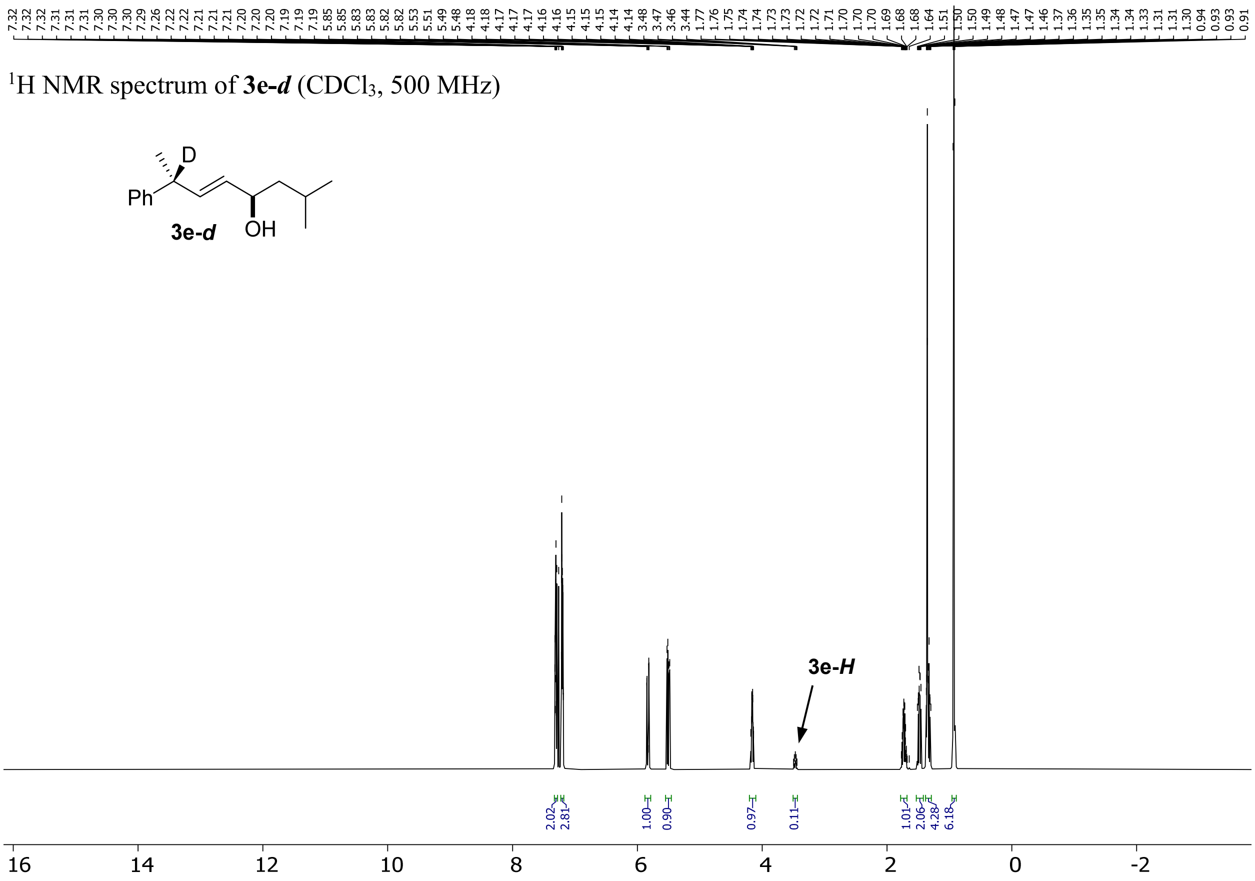

^{13}C{^{1}H} NMR spectrum of **3e-*d*** ($CDCl_3$, 126 MHz)

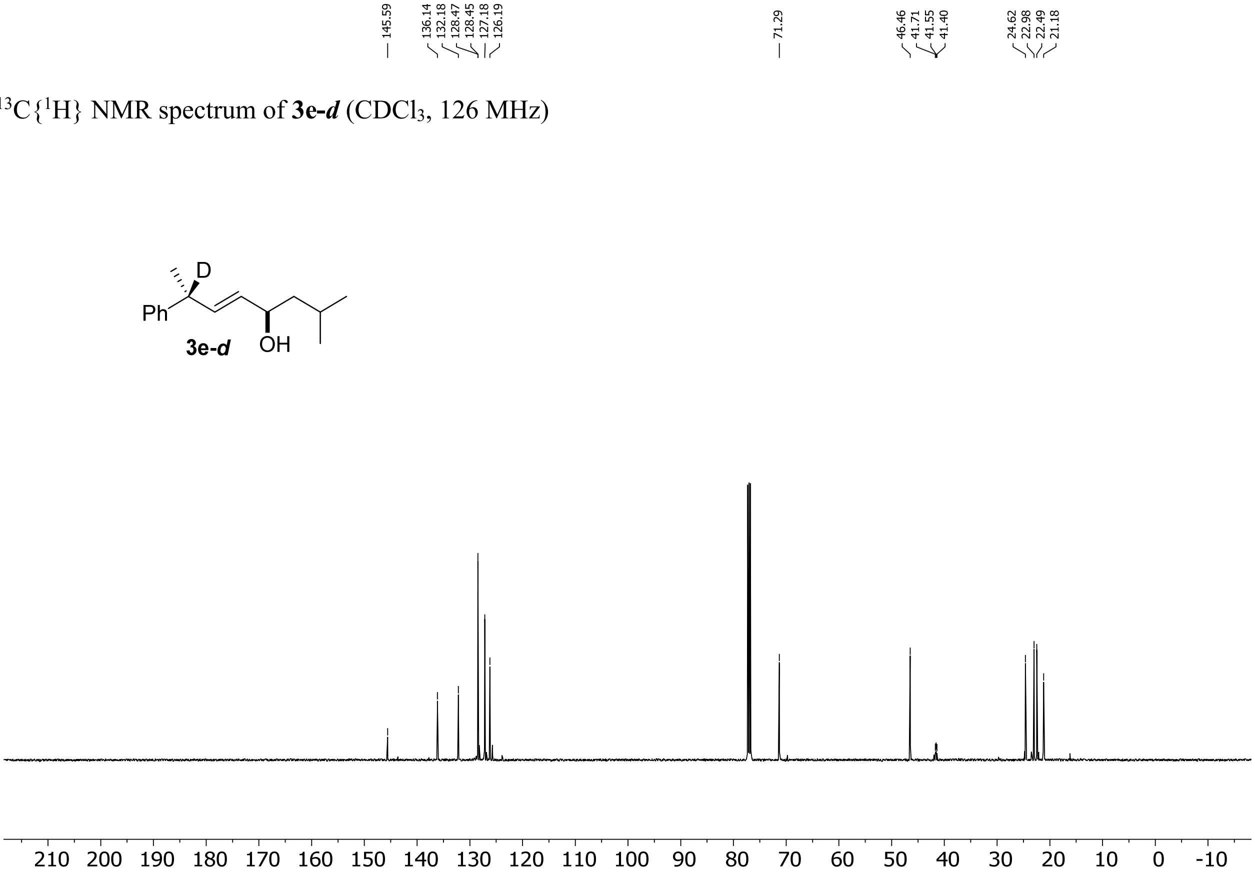

^{1}H NMR spectrum of **3f** ($CDCl_3$, 500 MHz)

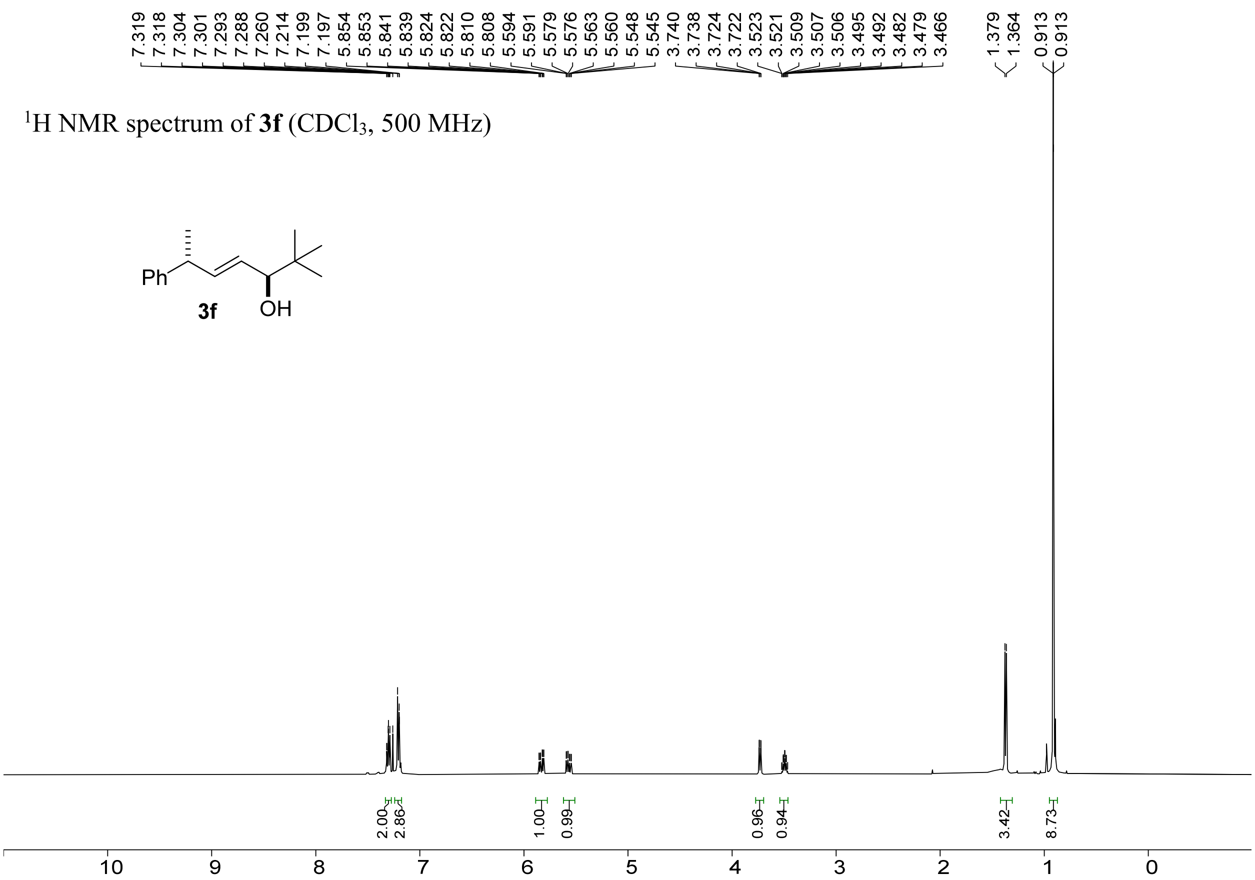

^{13}C{^{1}H} NMR spectrum of **3f** ($CDCl_3$, 126 MHz)

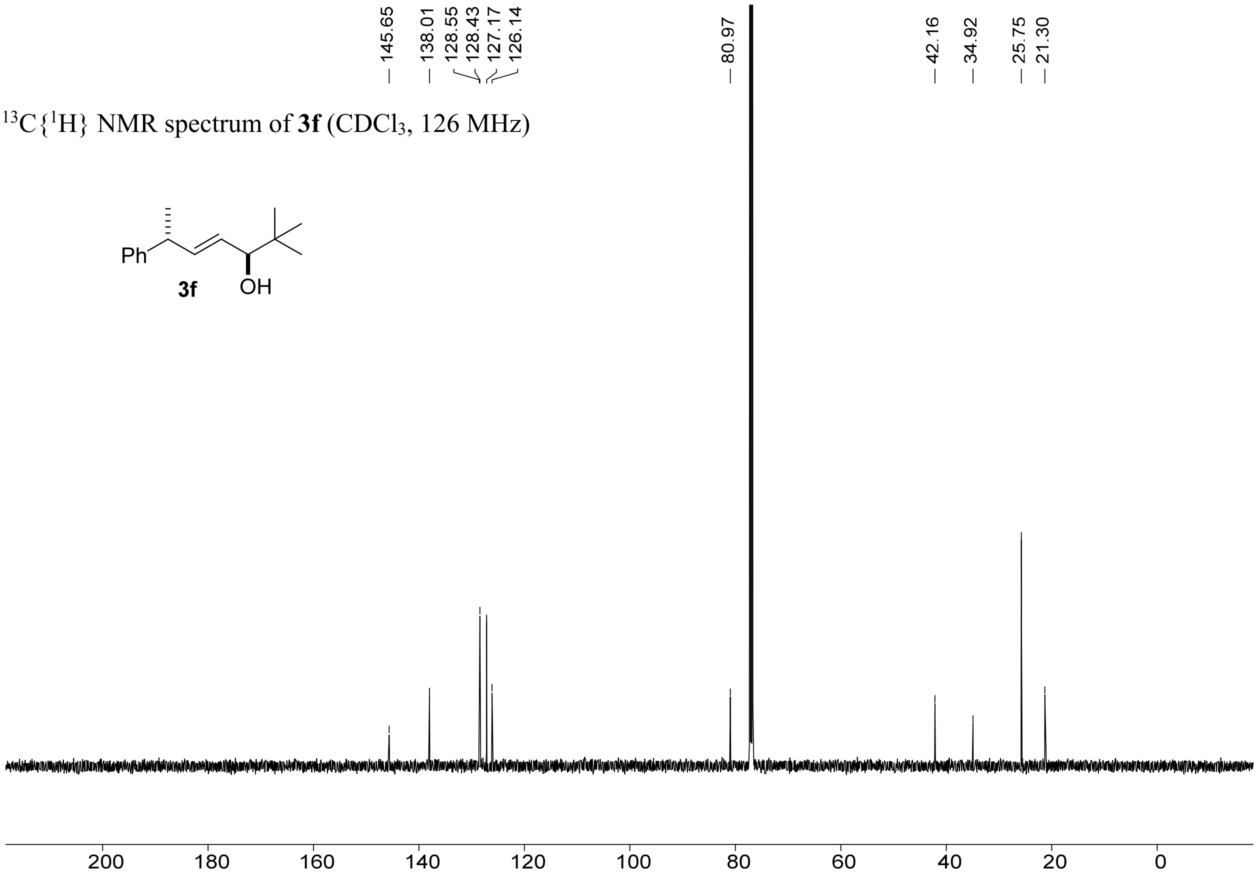

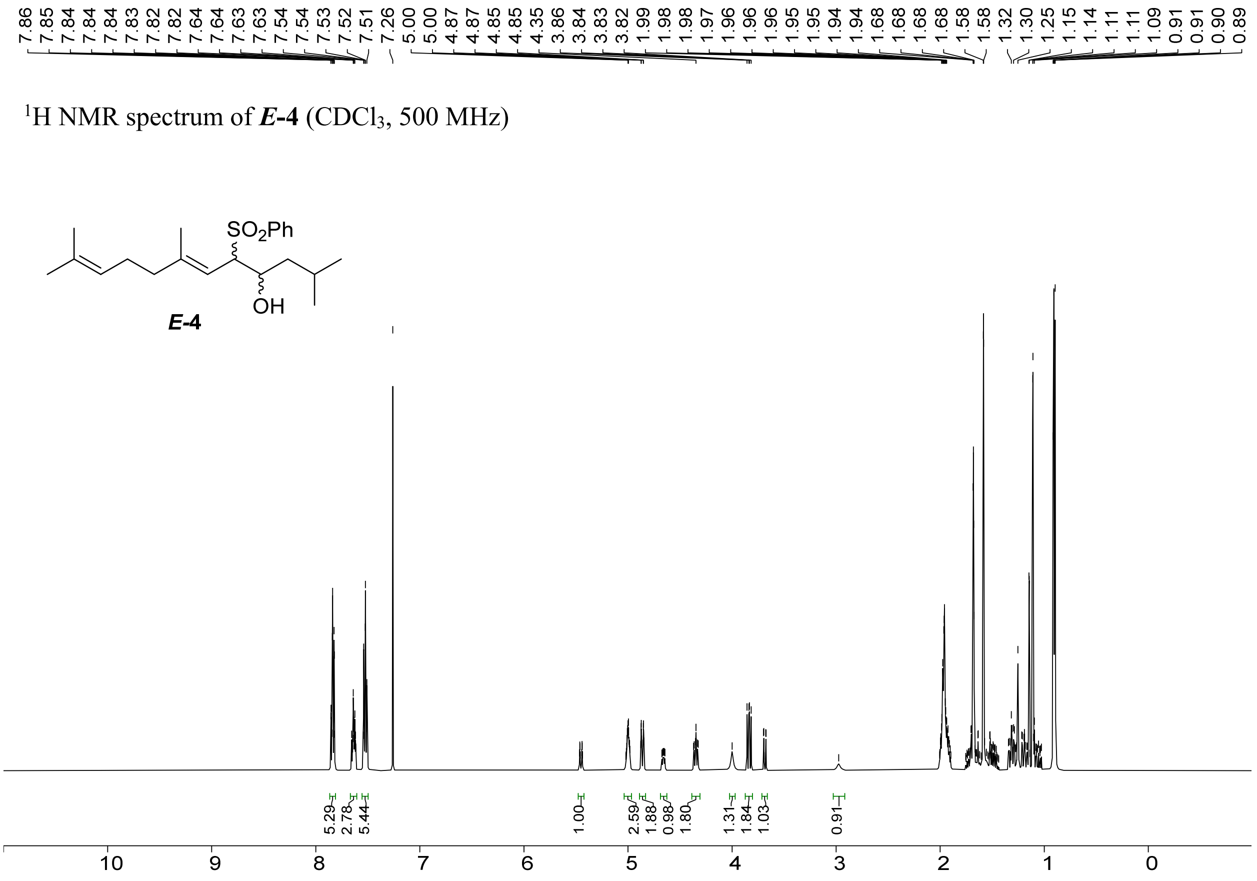

^{1}H NMR spectrum of ***E*-4** ($CDCl_3$, 500 MHz)

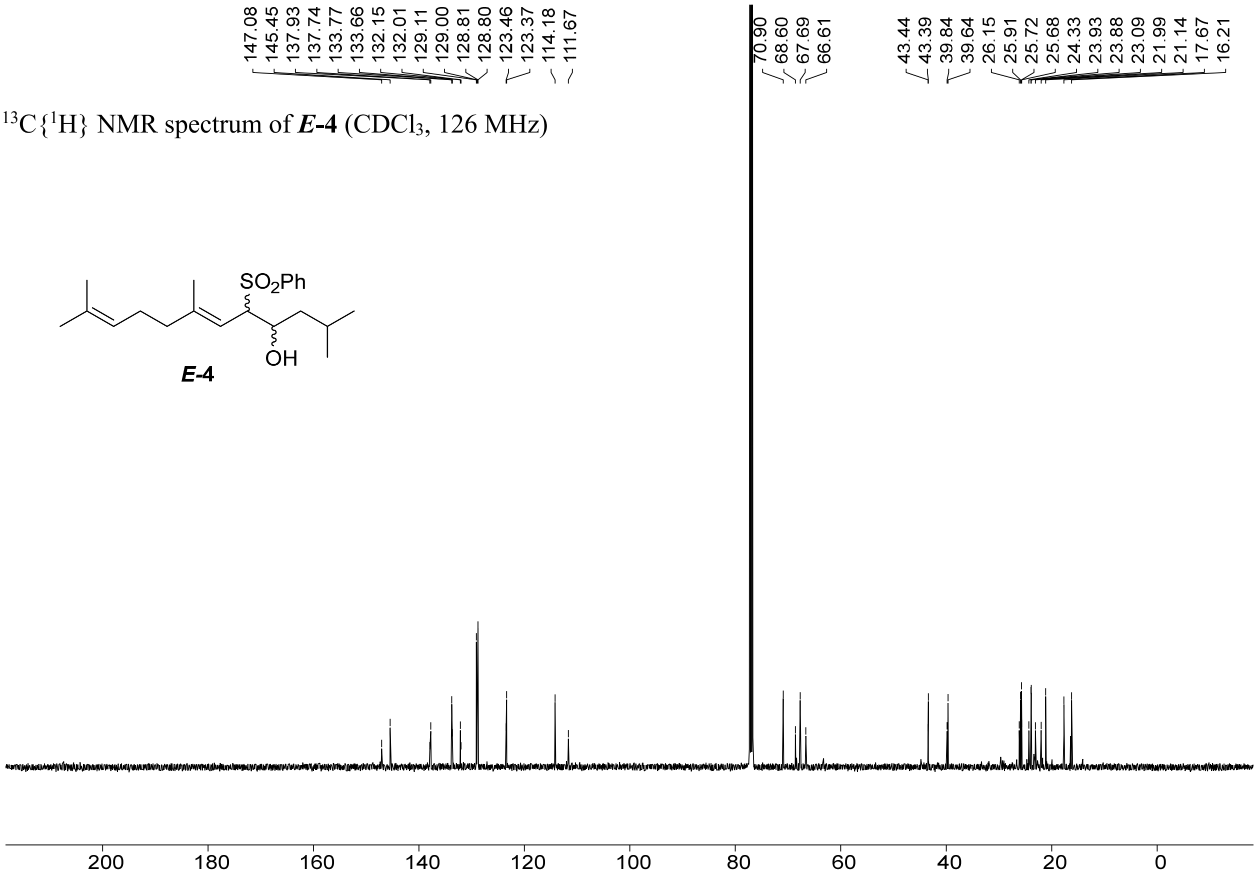

^{13}C{^{1}H} NMR spectrum of ***E*-4** ($CDCl_3$, 126 MHz)

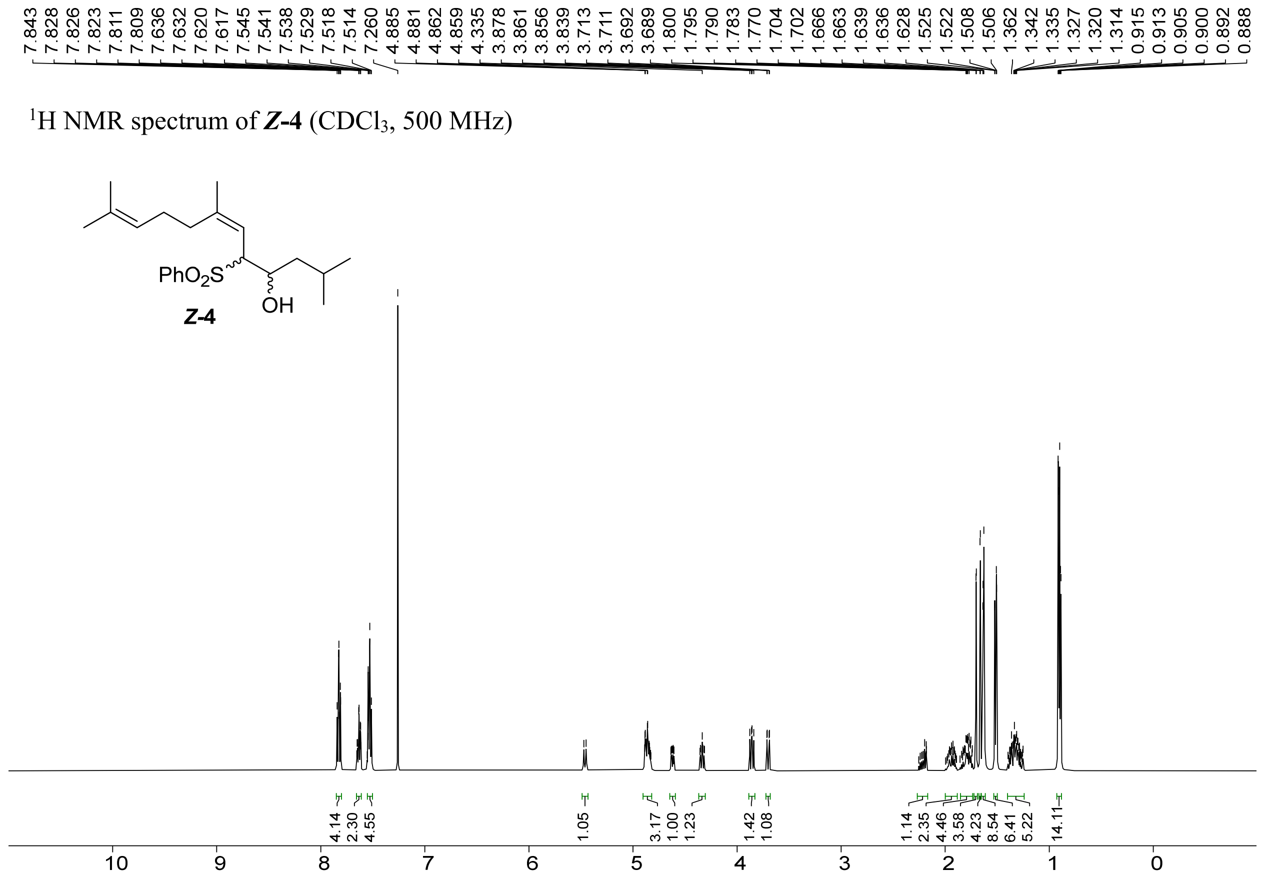

^{1}H NMR spectrum of ***Z*-4** ($CDCl_3$, 500 MHz)

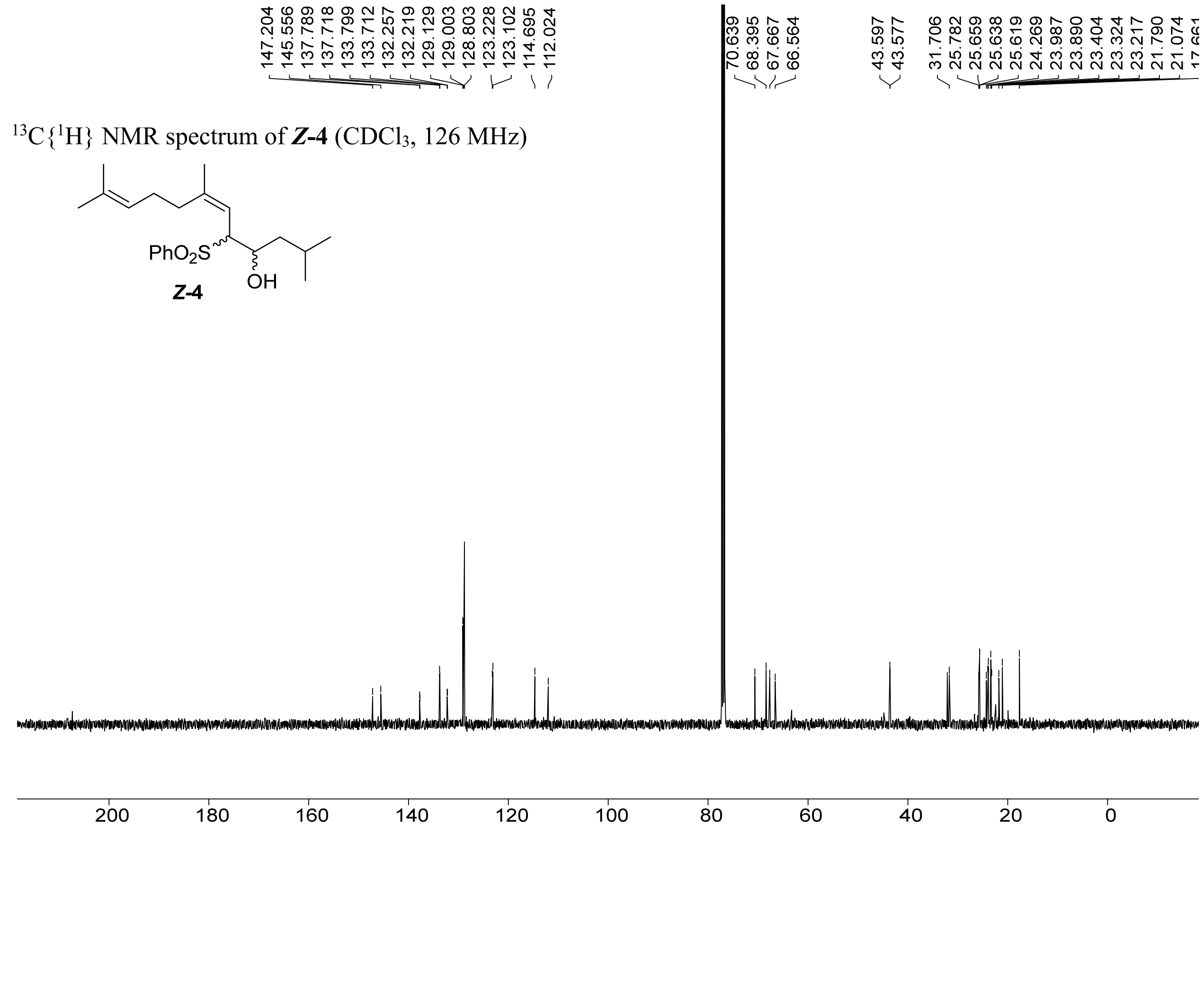

^{13}C{^{1}H} NMR spectrum of ***Z*-4** ($CDCl_3$, 126 MHz)

^{1}H NMR spectrum of **5-*anti*** ($CDCl_3$, 500 MHz)

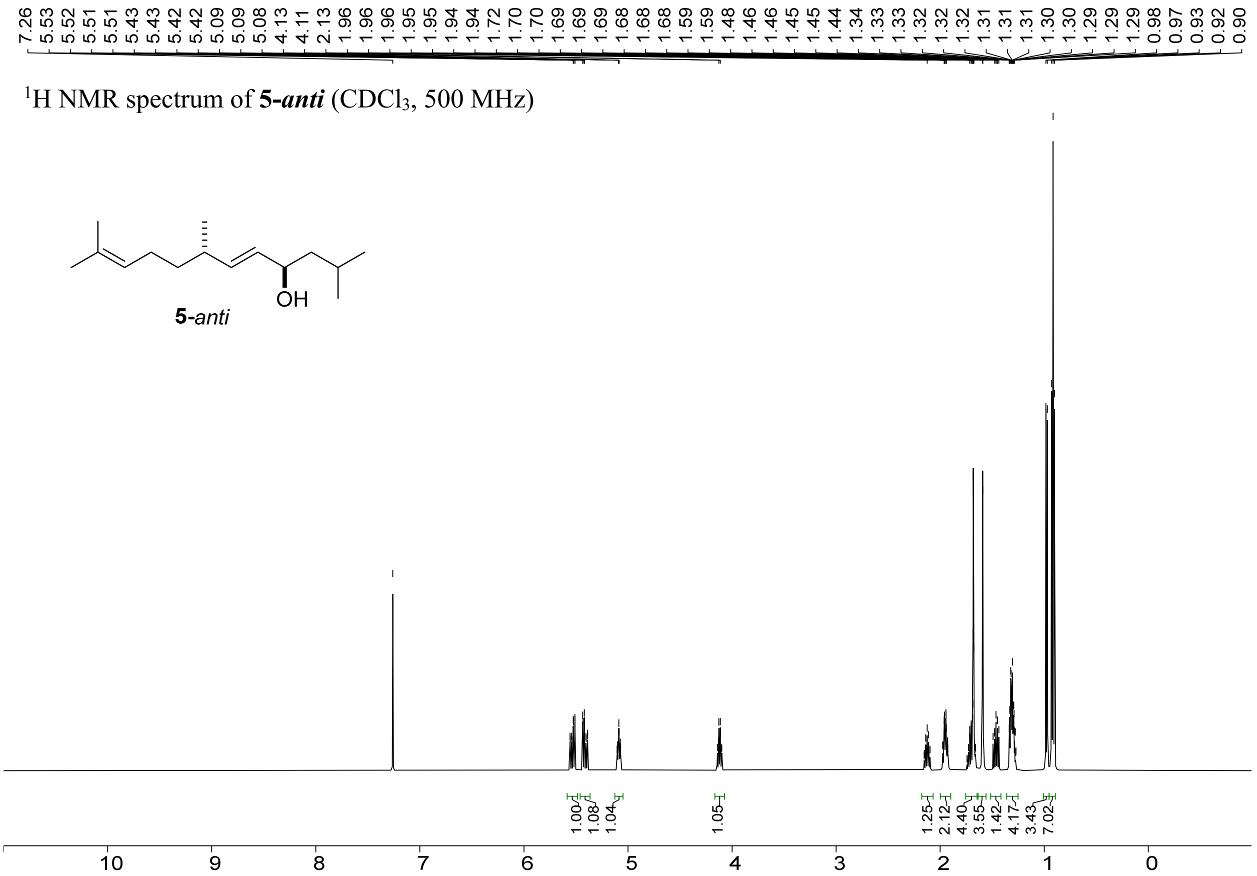

^{13}C{^{1}H} NMR spectrum of **5-*anti*** ($CDCl_3$, 126 MHz)

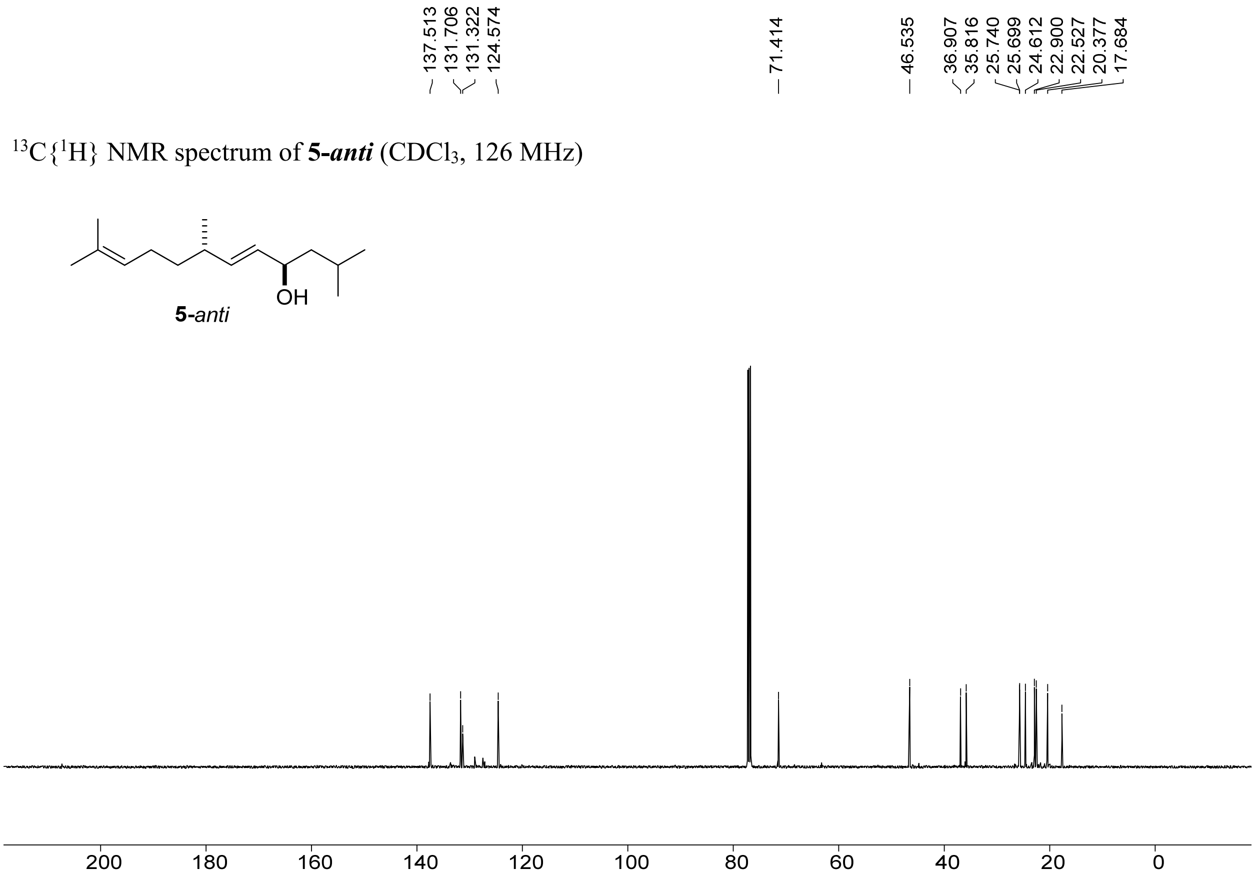

^{1}H NMR spectrum of **5-*syn*** ($CDCl_3$, 500 MHz)

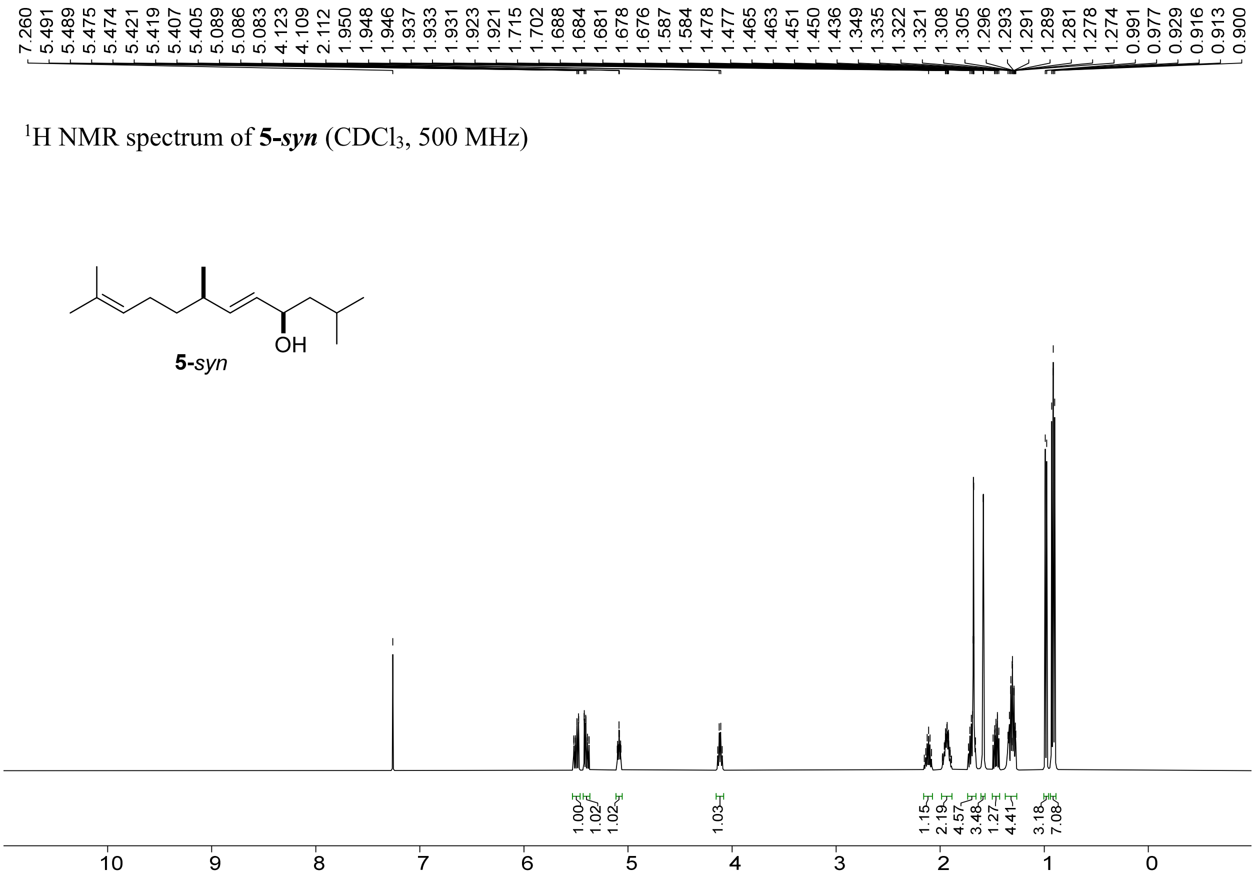

^{13}C{^{1}H} NMR spectrum of **5-*syn*** ($CDCl_3$, 126 MHz)

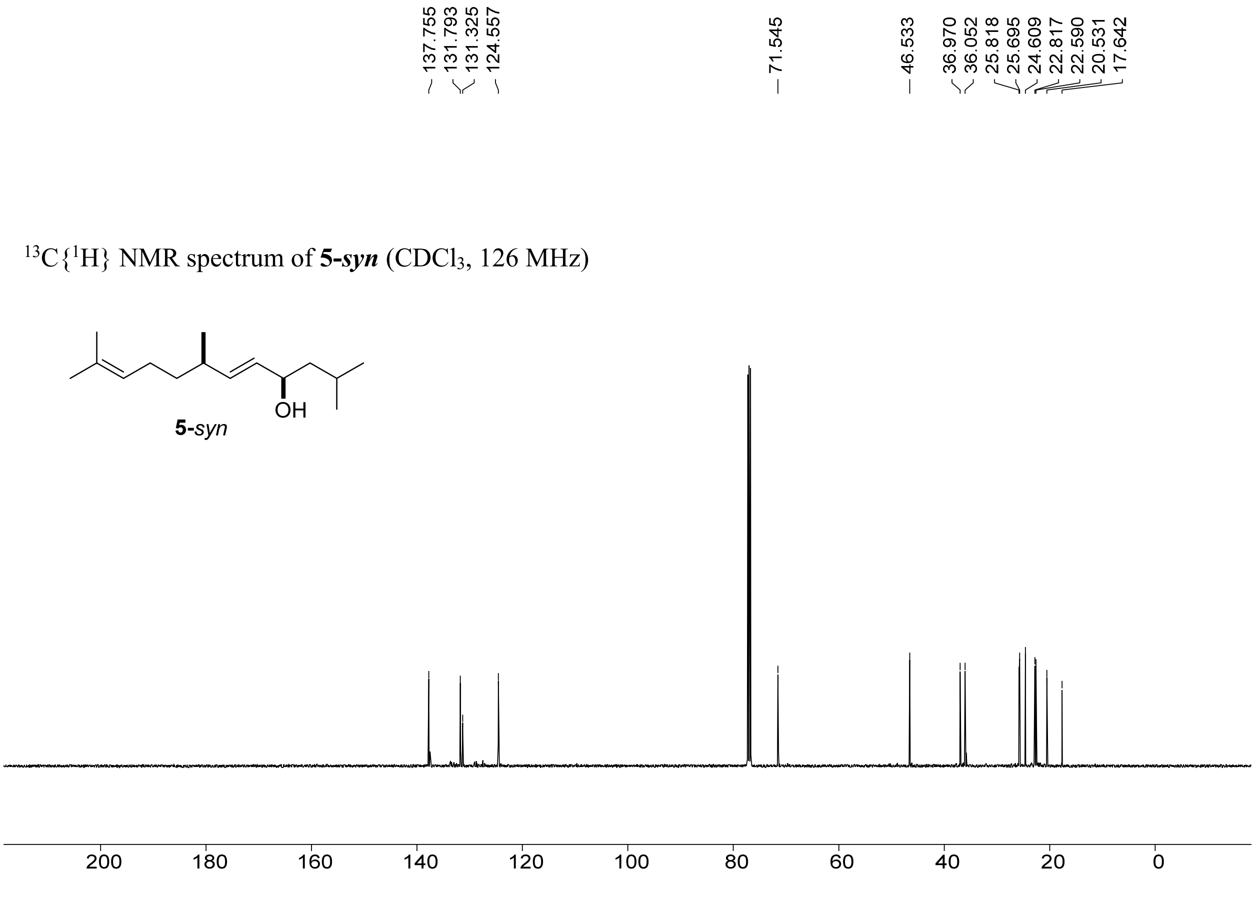

^{1}H NMR spectrum of **6** ($CDCl_3$, 500 MHz)

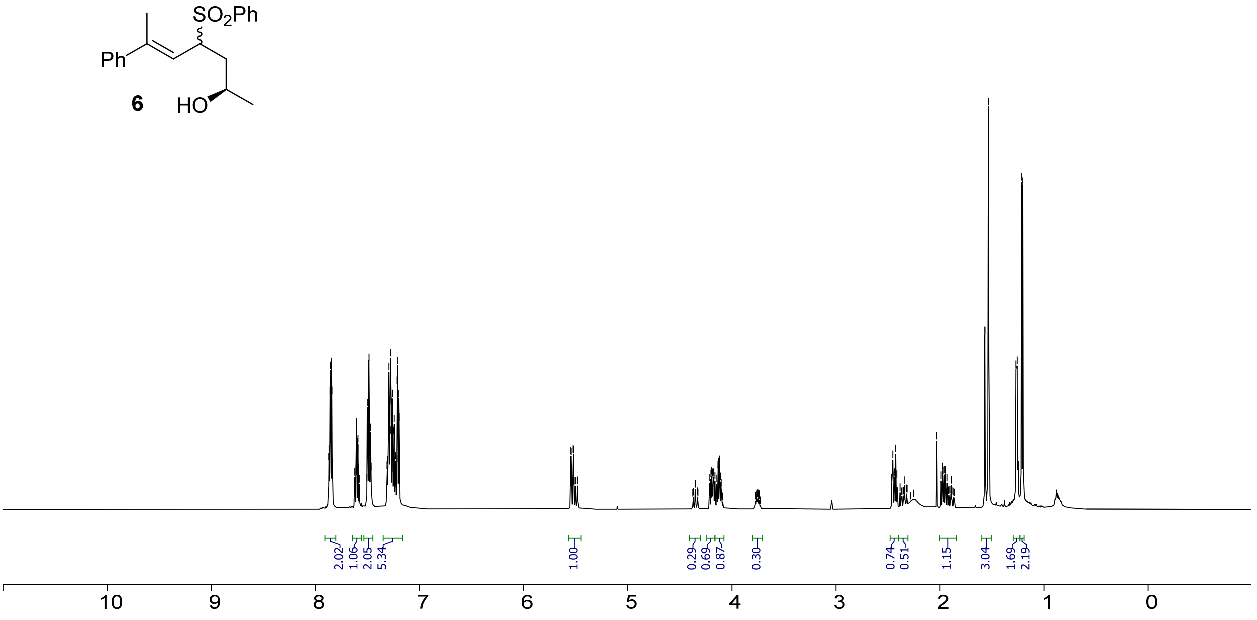

^{13}C{^{1}H} NMR spectrum of **6** ($CDCl_3$, 126 MHz)

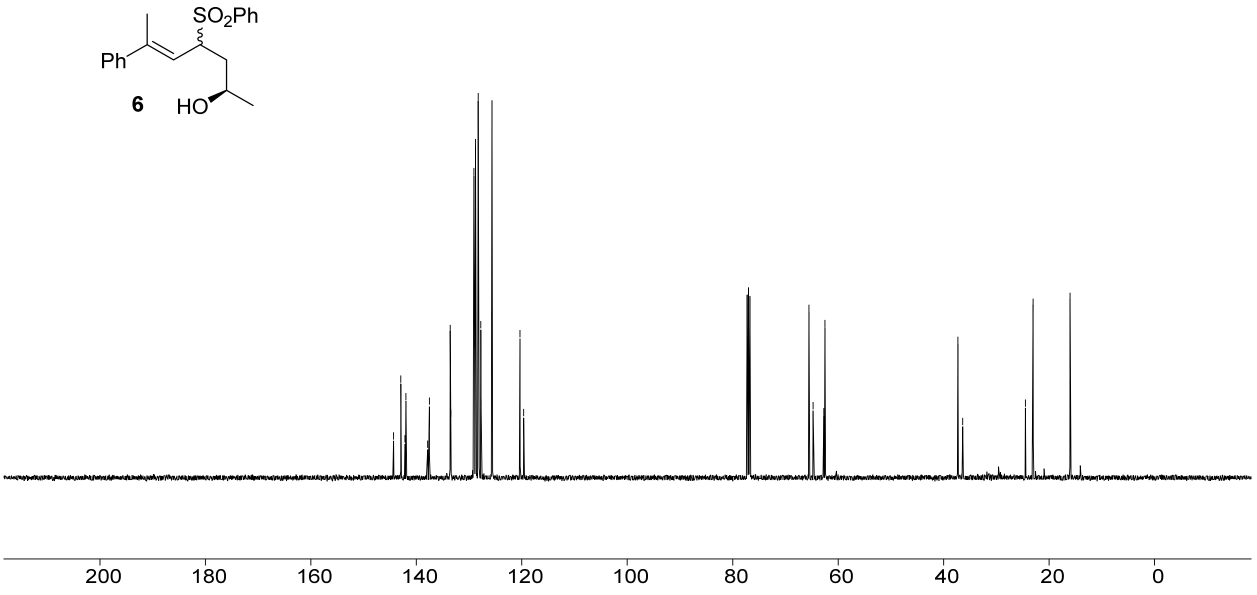

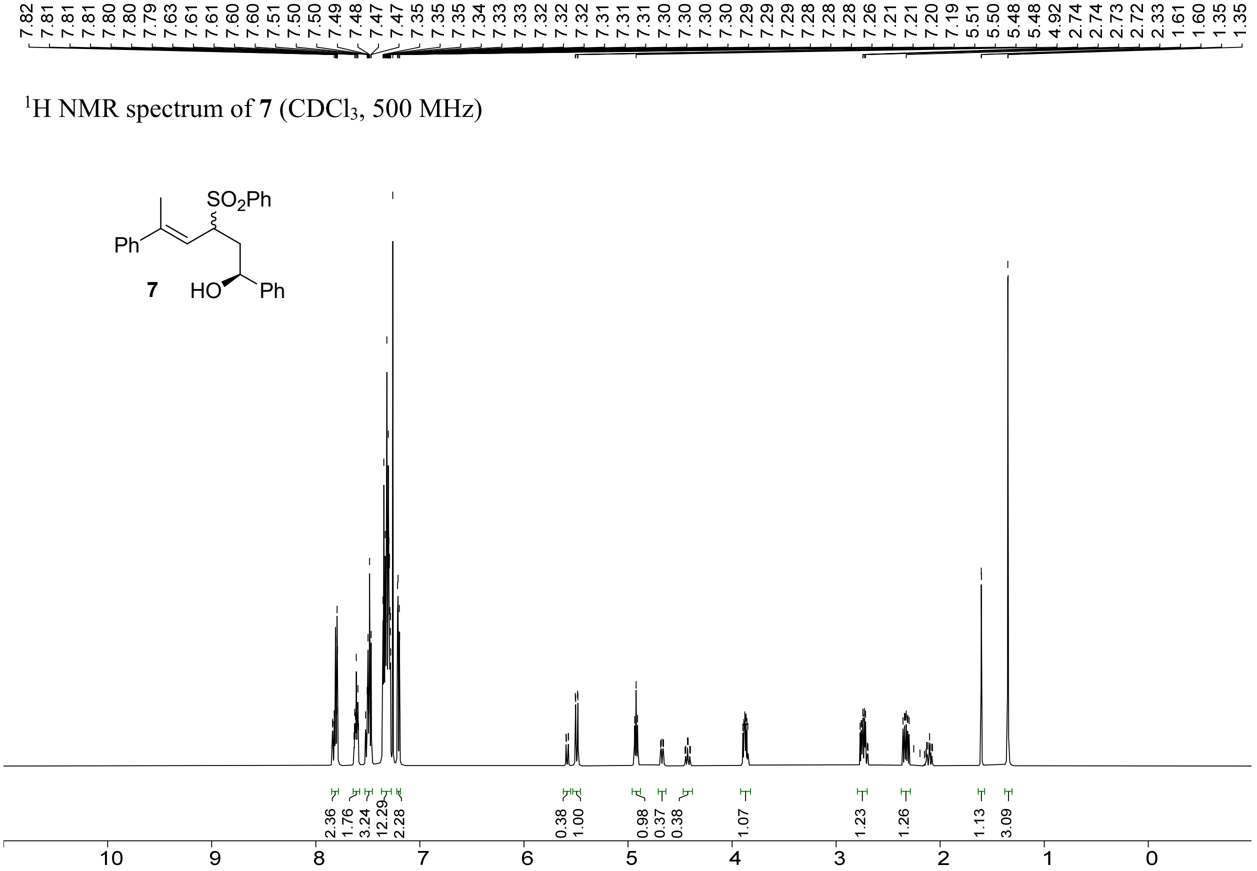

^{1}H NMR spectrum of **7** ($CDCl_3$, 500 MHz)

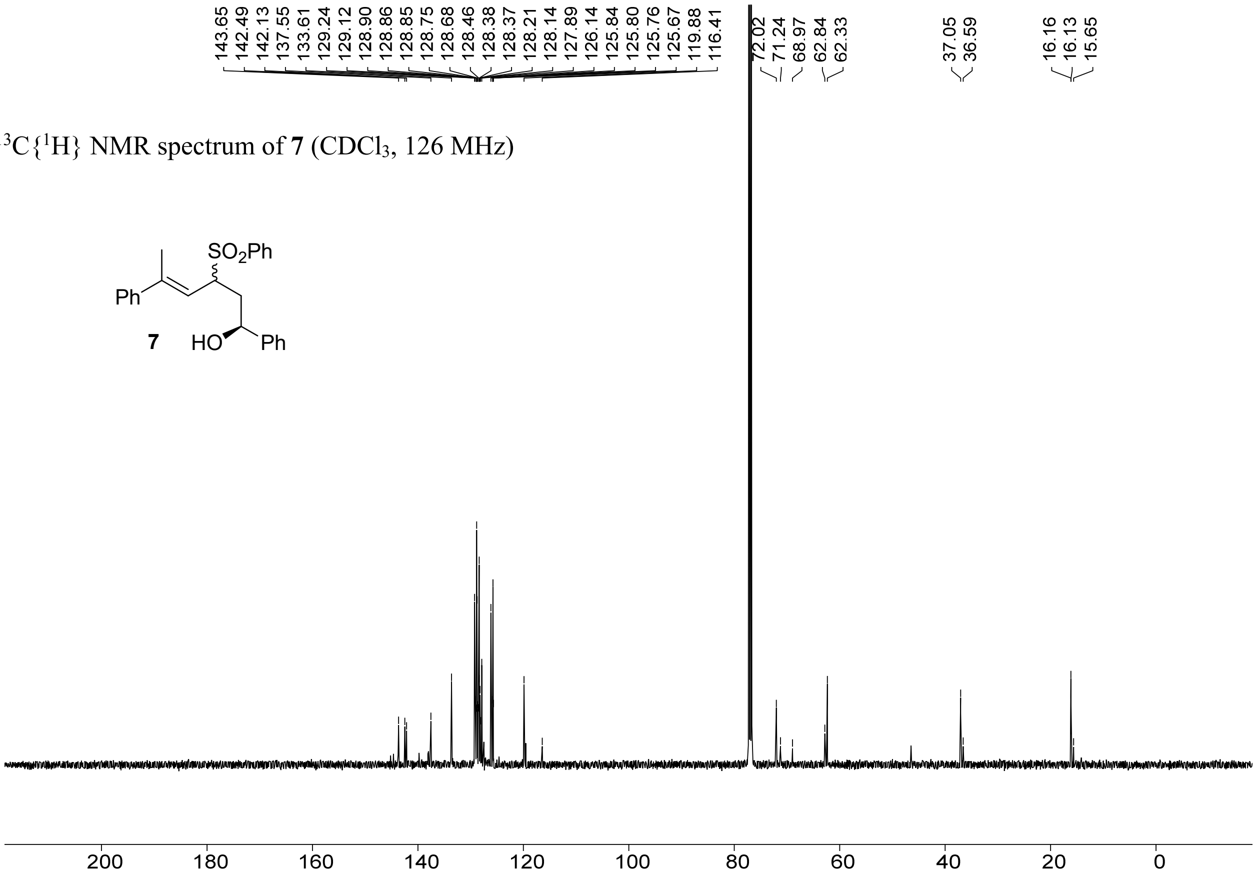

^{13}C{^{1}H} NMR spectrum of **7** ($CDCl_3$, 126 MHz)

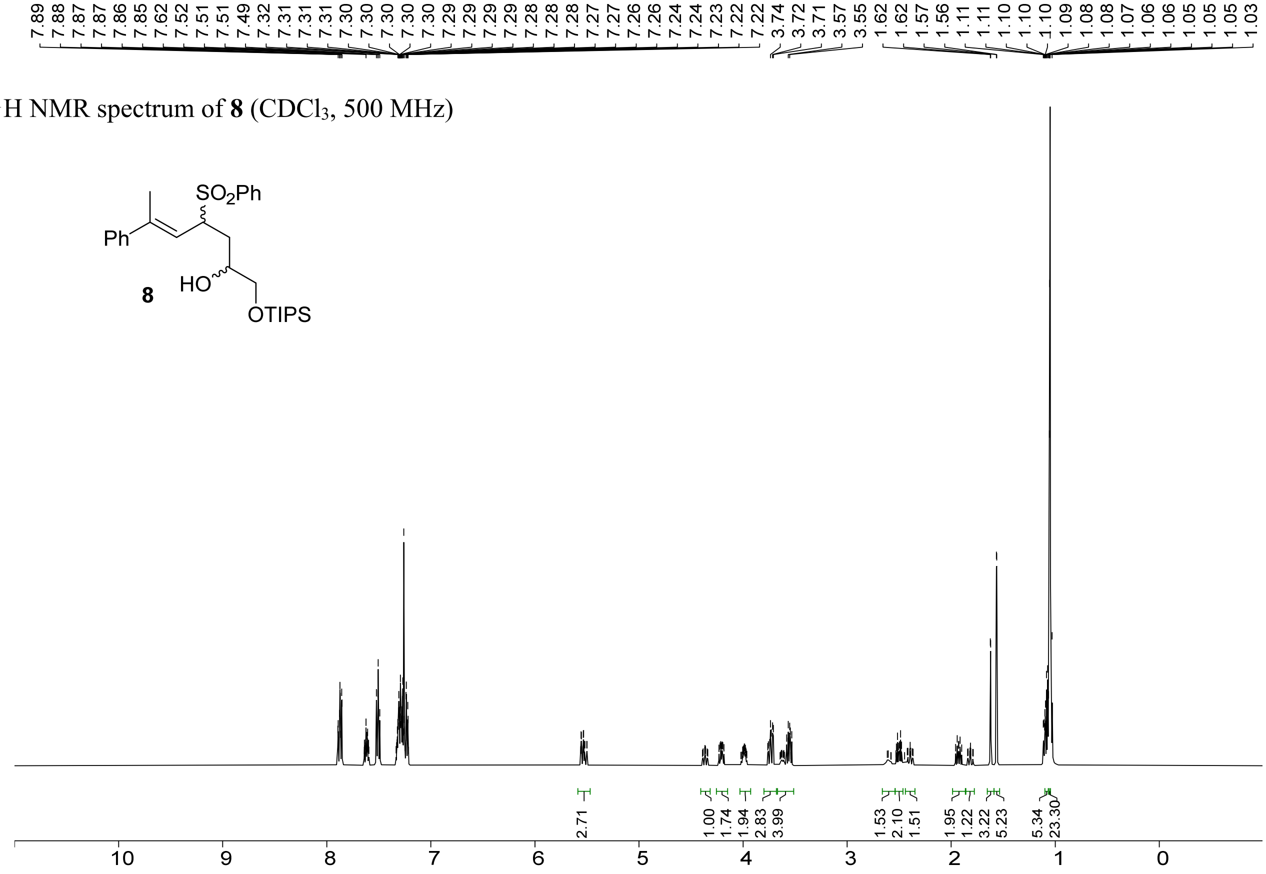

^{1}H NMR spectrum of **8** ($CDCl_3$, 500 MHz)

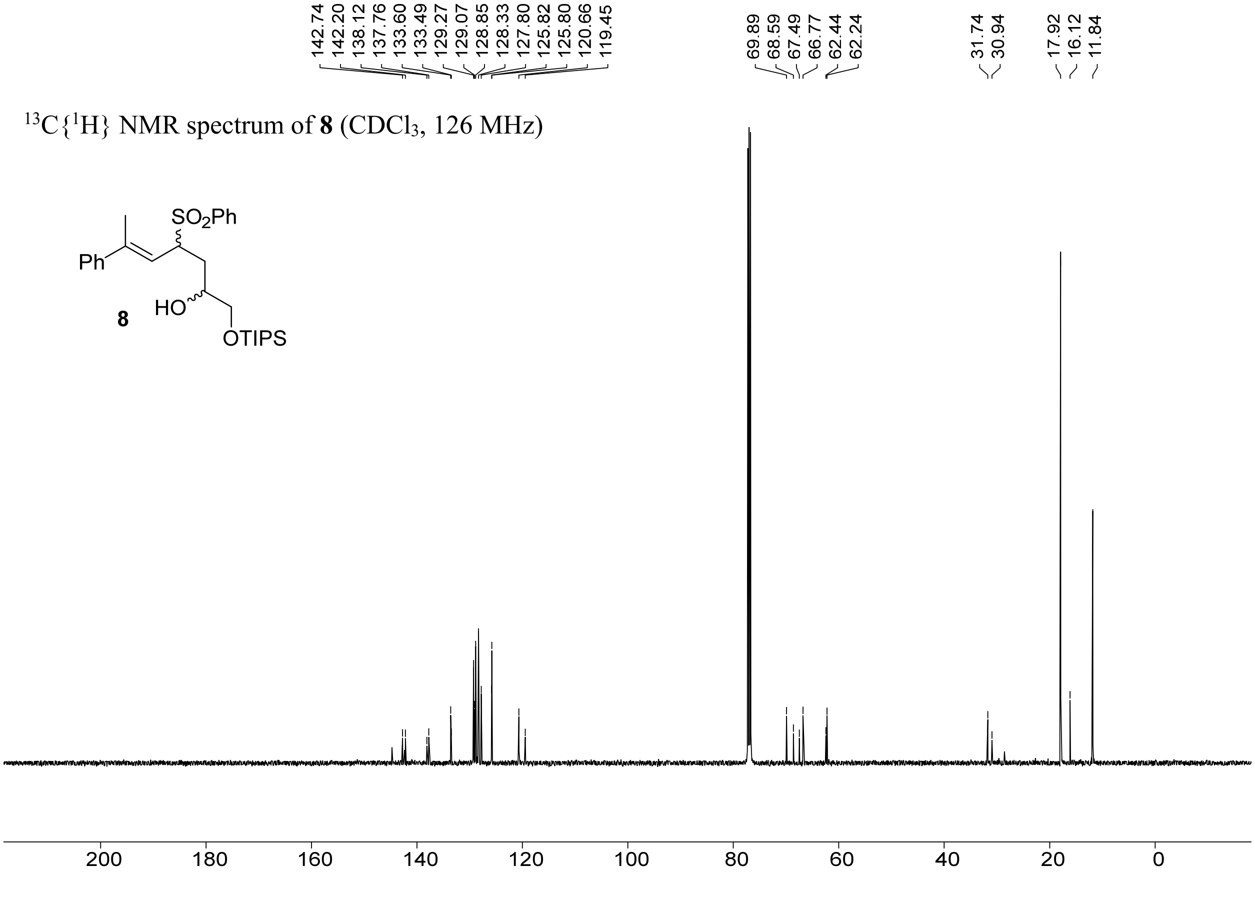

$^{13}C\{^{1}H\}$ NMR spectrum of **8** ($CDCl_3$, 126 MHz)

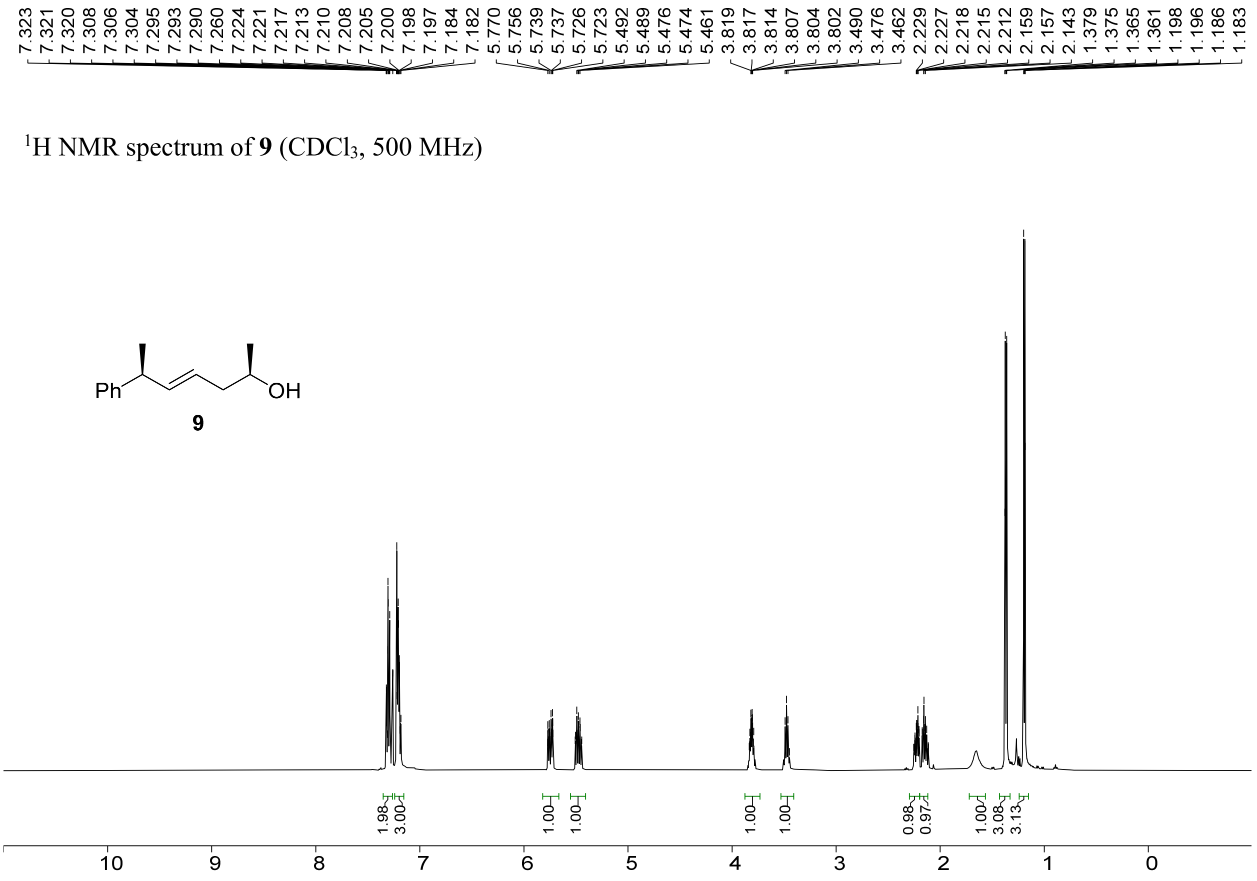

^{1}H NMR spectrum of **9** ($CDCl_3$, 500 MHz)

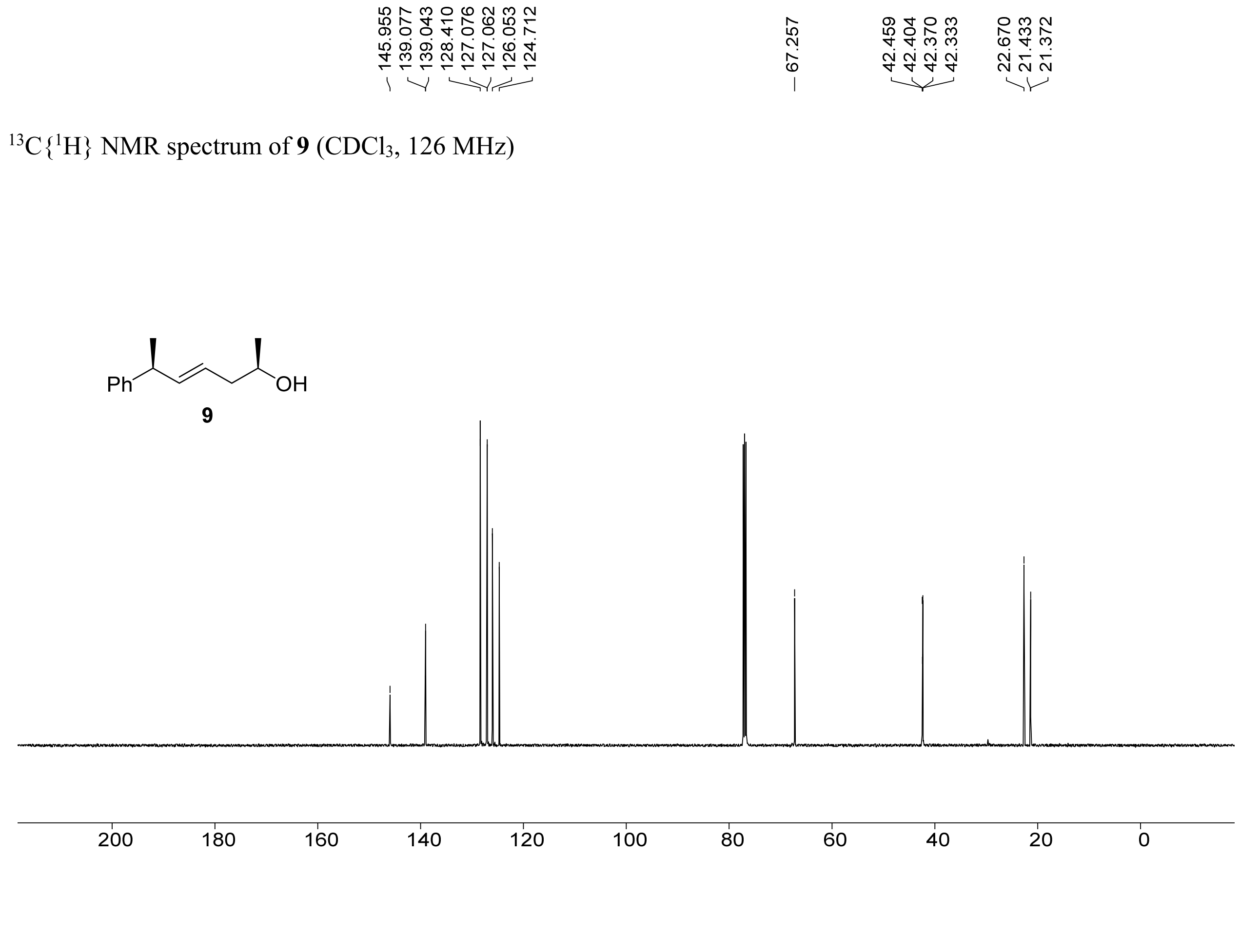

^{13}C{^{1}H} NMR spectrum of **9** ($CDCl_3$, 126 MHz)

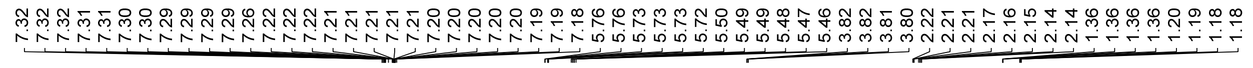

^{1}H NMR spectrum of **9-*d*** ($CDCl_3$, 500 MHz)

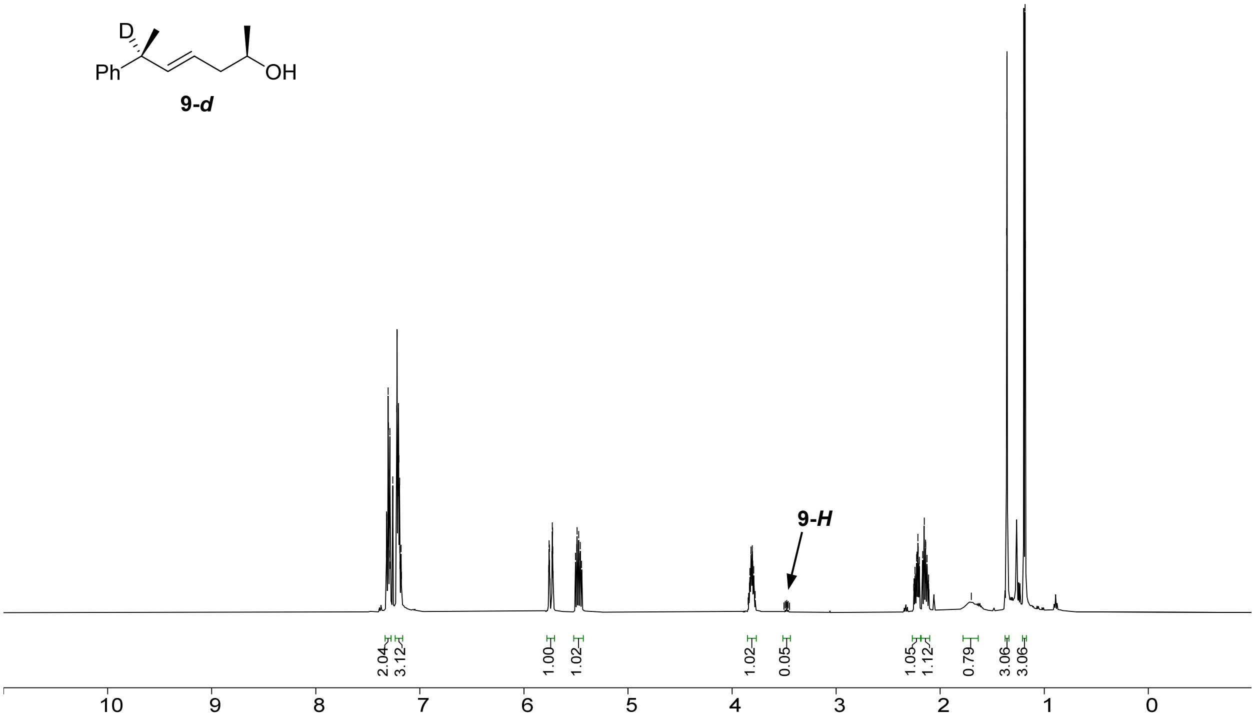

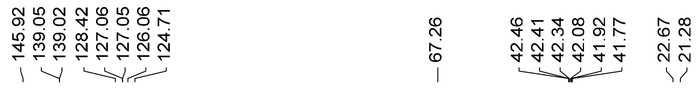

^{13}C{^{1}H} NMR spectrum of **9-*d*** ($CDCl_3$, 126 MHz)

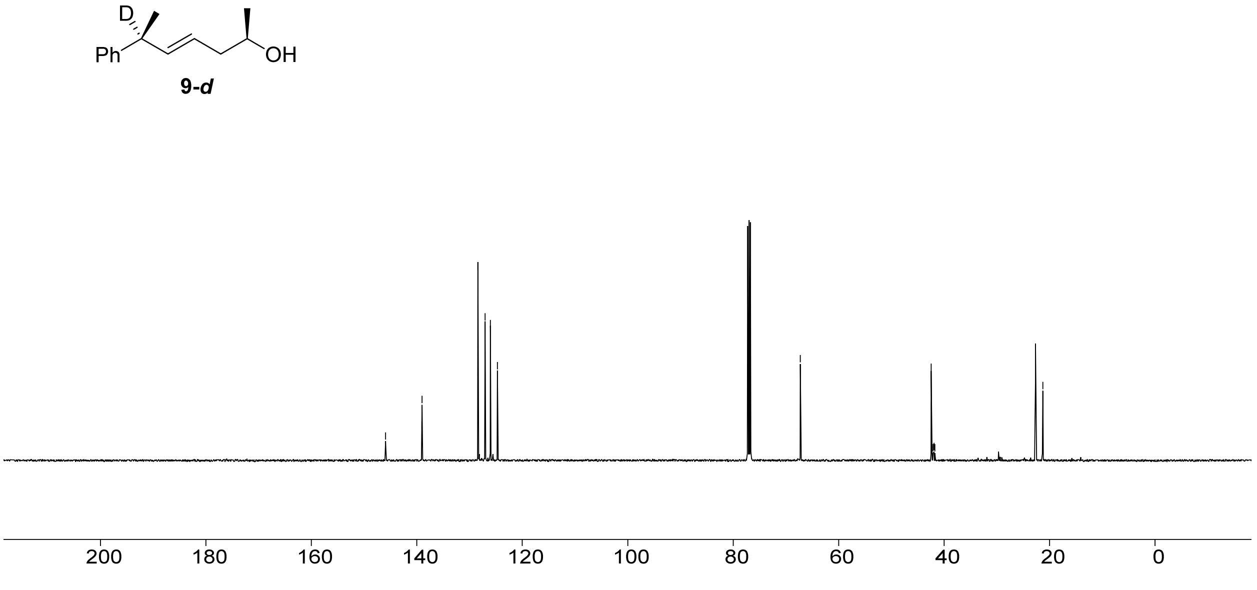

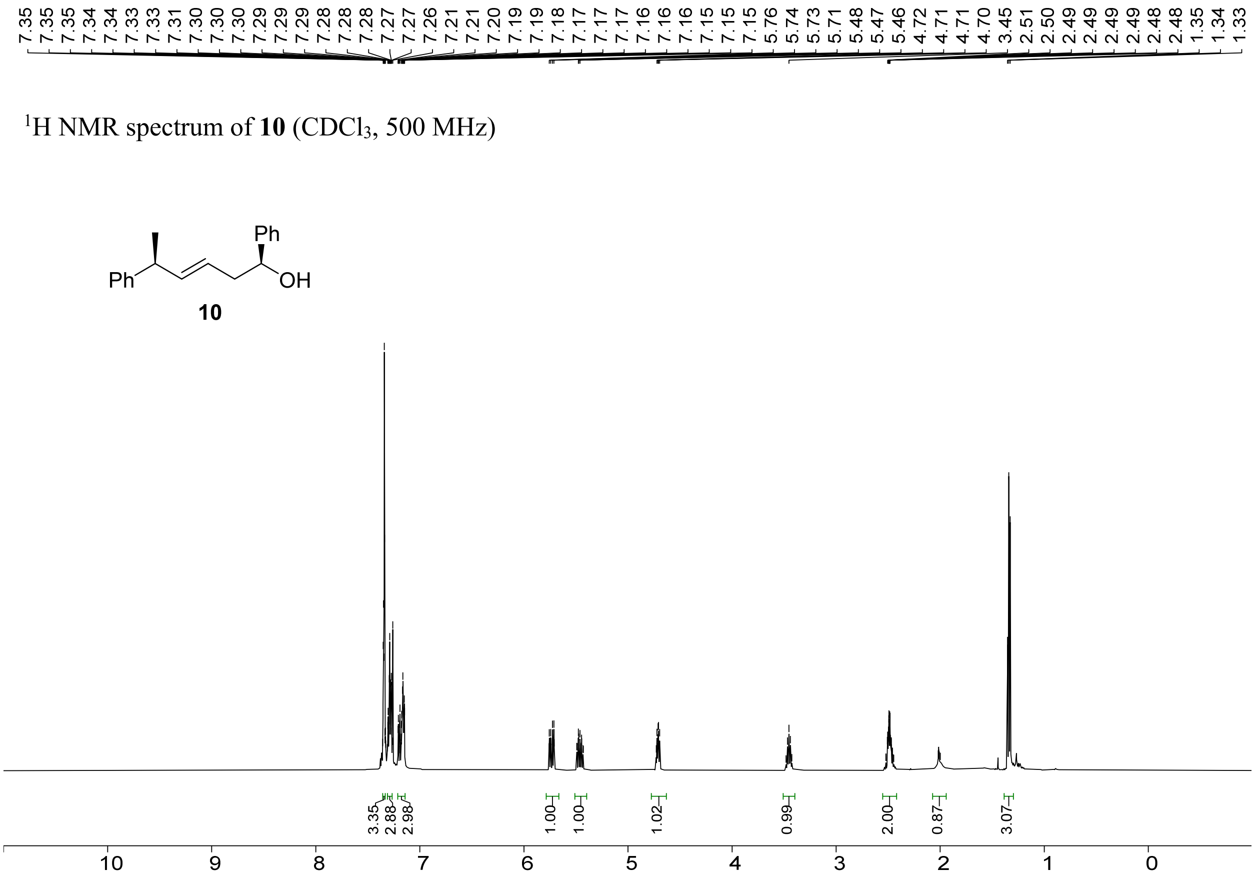

^{1}H NMR spectrum of **10** ($CDCl_3$, 500 MHz)

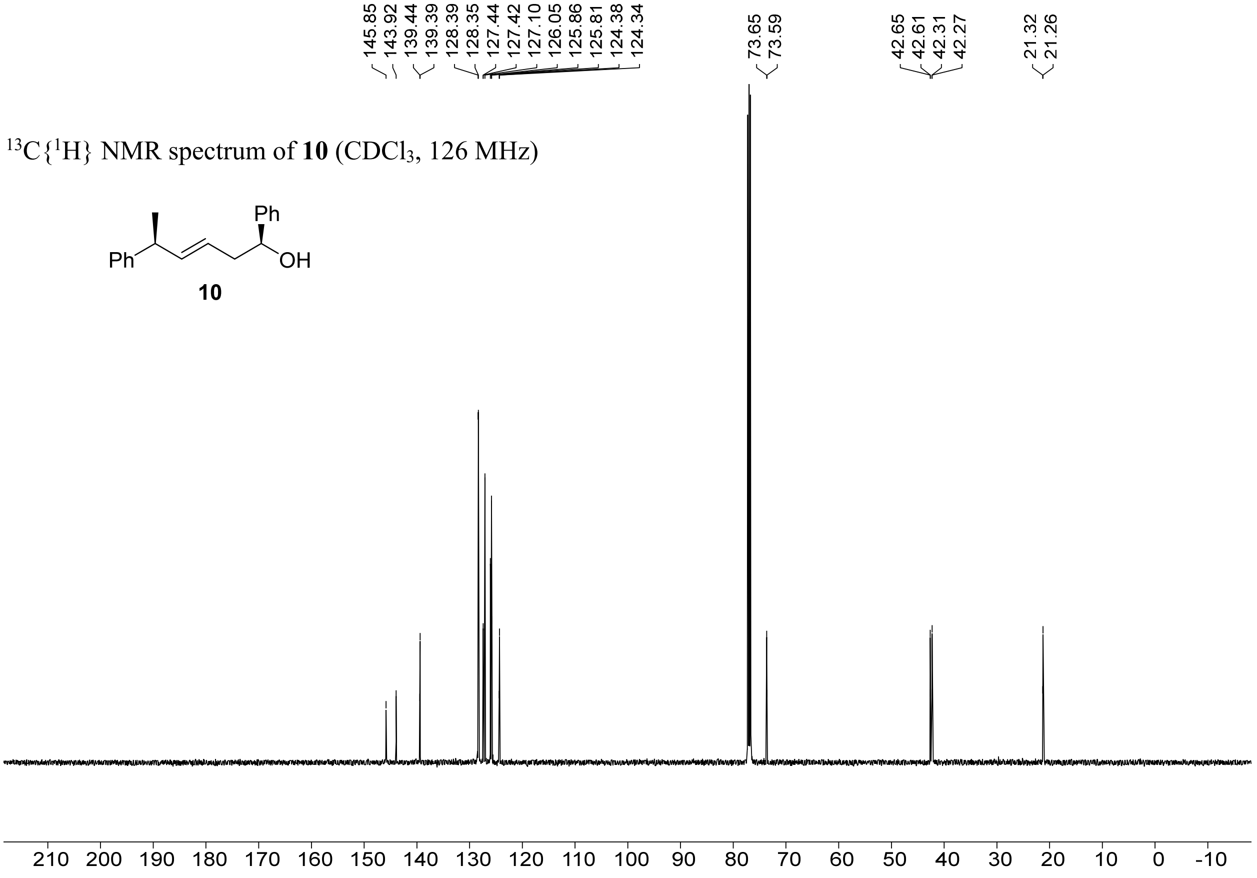

^{13}C{^{1}H} NMR spectrum of **10** ($CDCl_3$, 126 MHz)

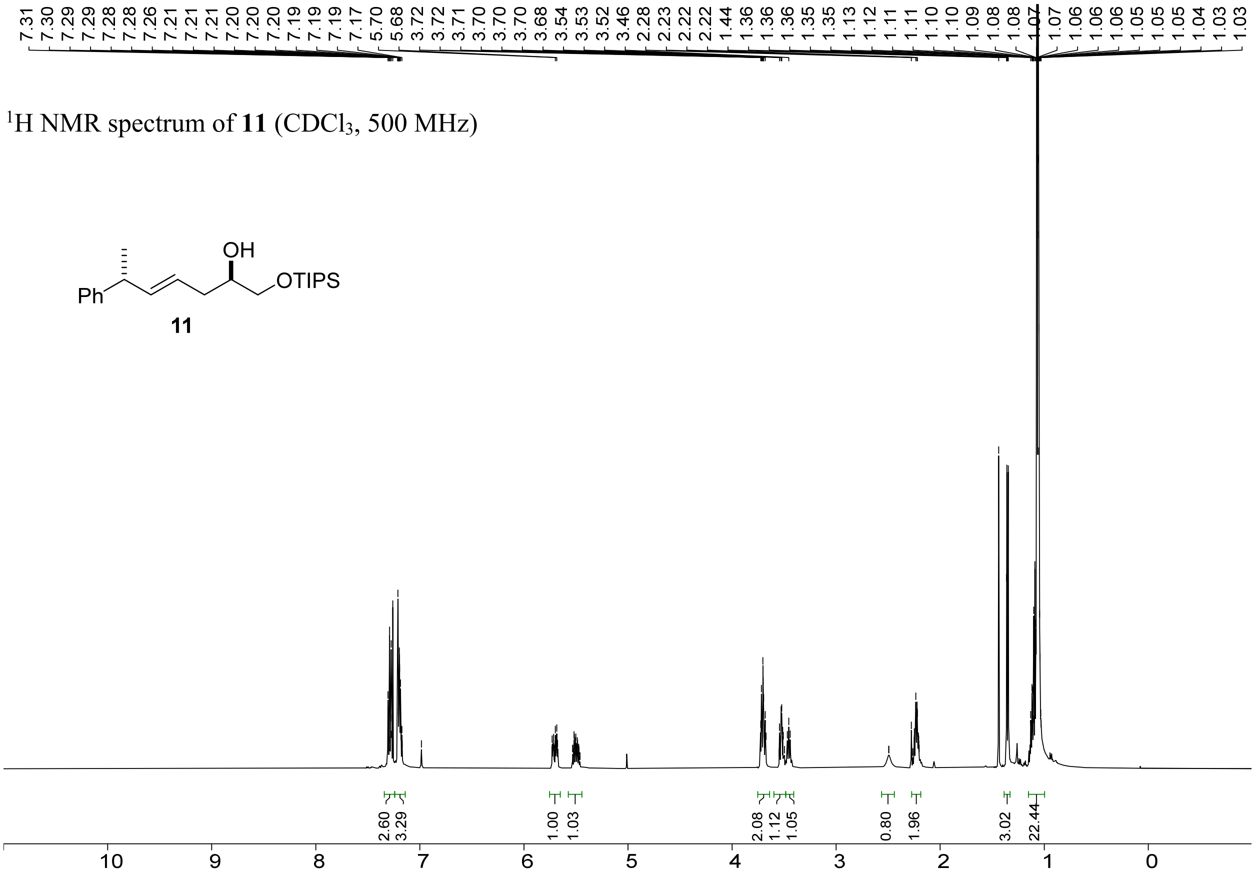

^{1}H NMR spectrum of **11** ($CDCl_3$, 500 MHz)

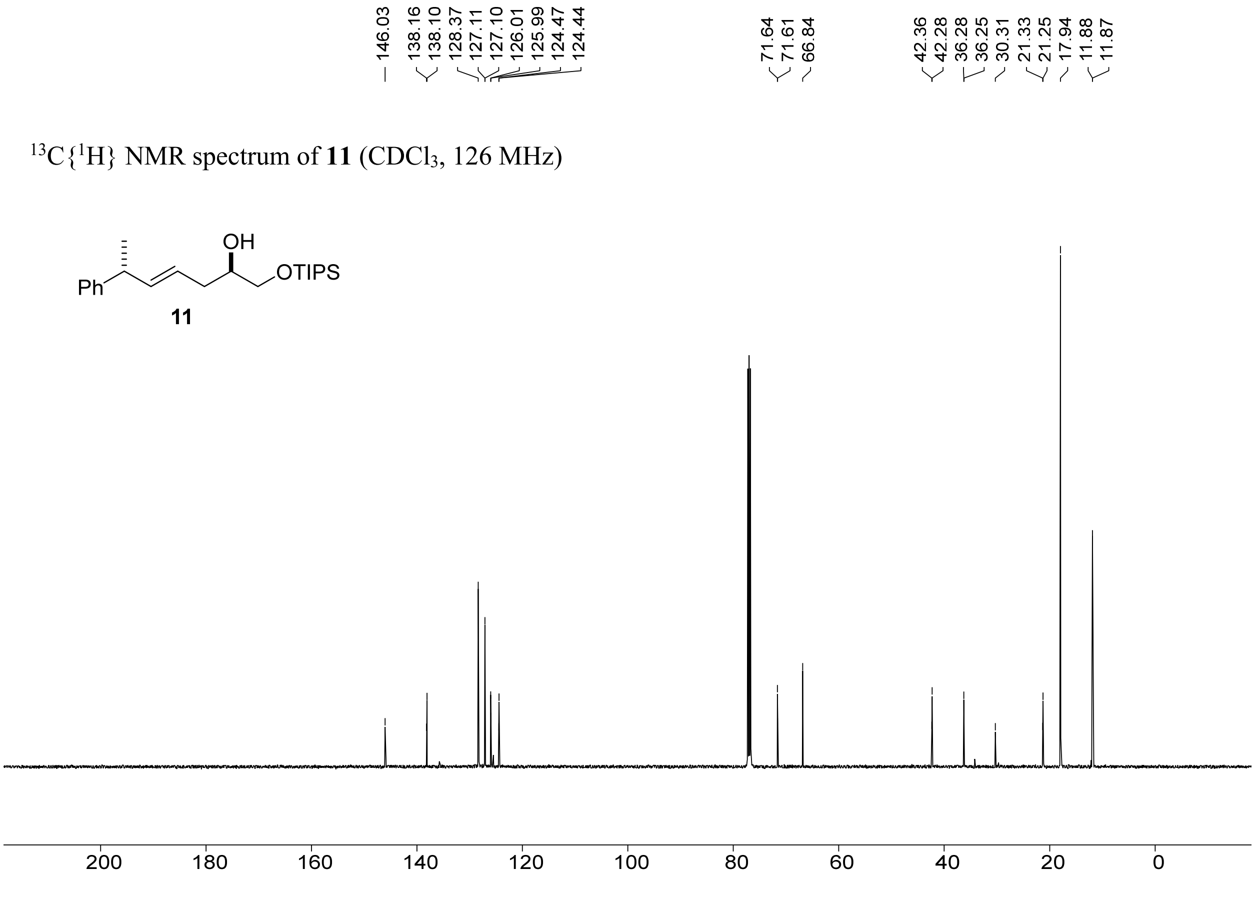

^{13}C{^{1}H} NMR spectrum of **11** ($CDCl_3$, 126 MHz)

^{1}H NMR spectrum of **17** ($CDCl_3$, 500 MHz)

17

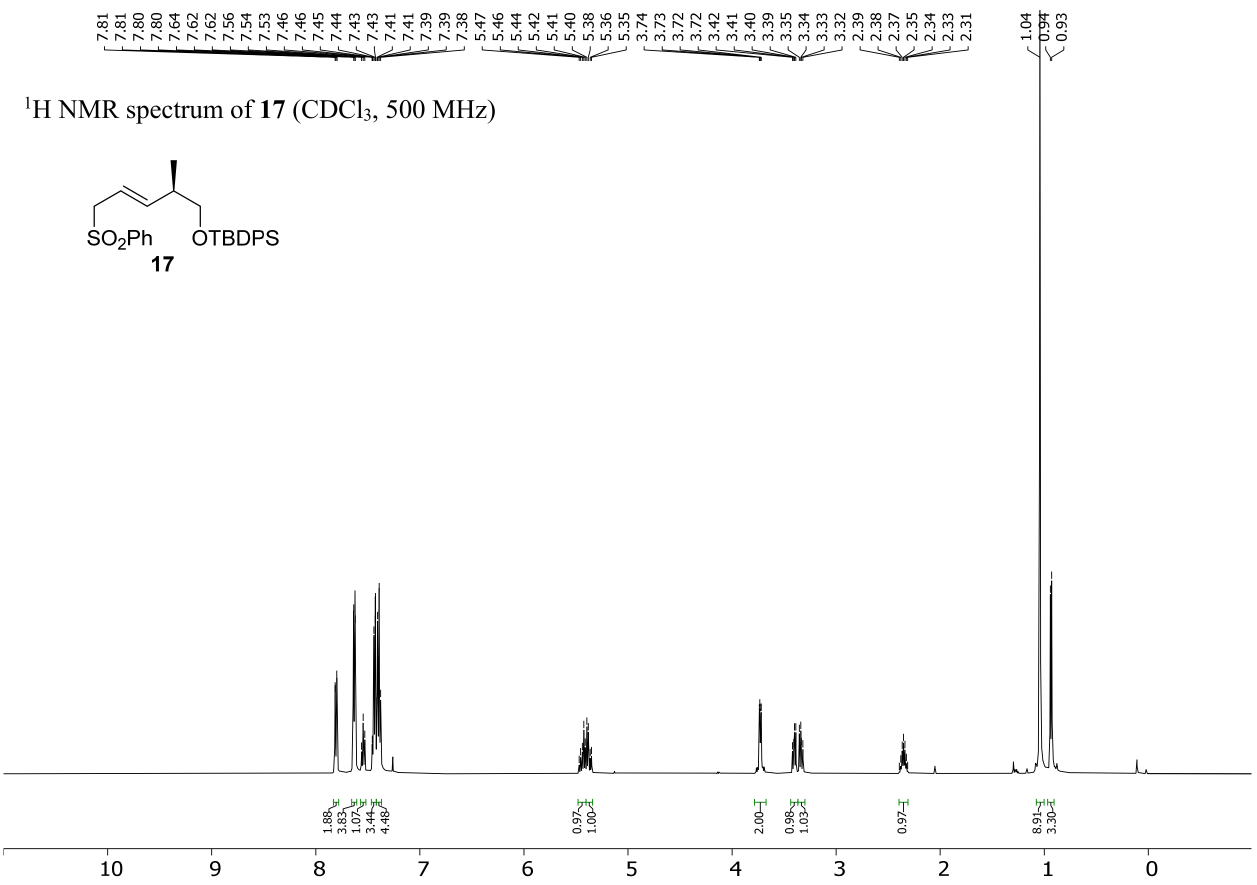

^{13}C{^{1}H} NMR spectrum of **17** ($CDCl_3$, 126 MHz)

17

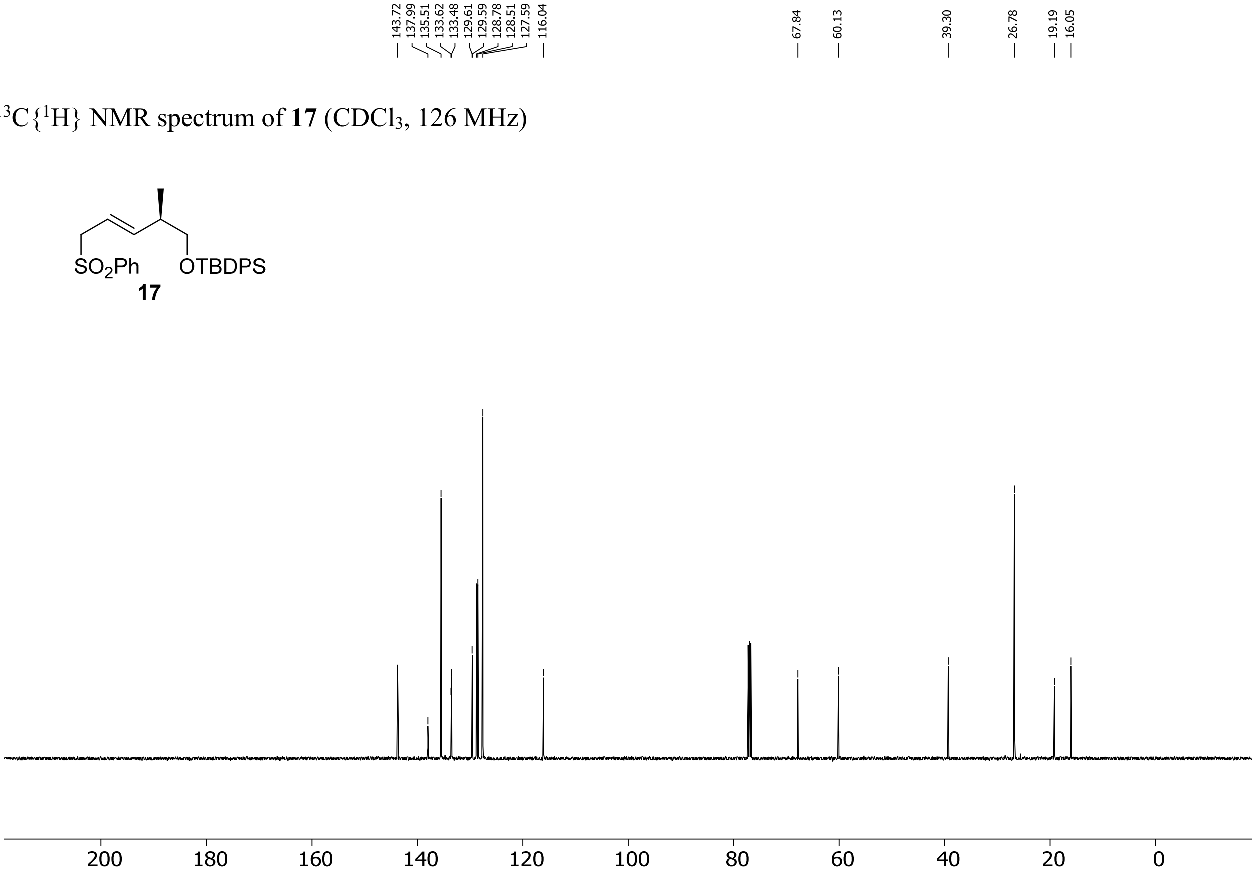

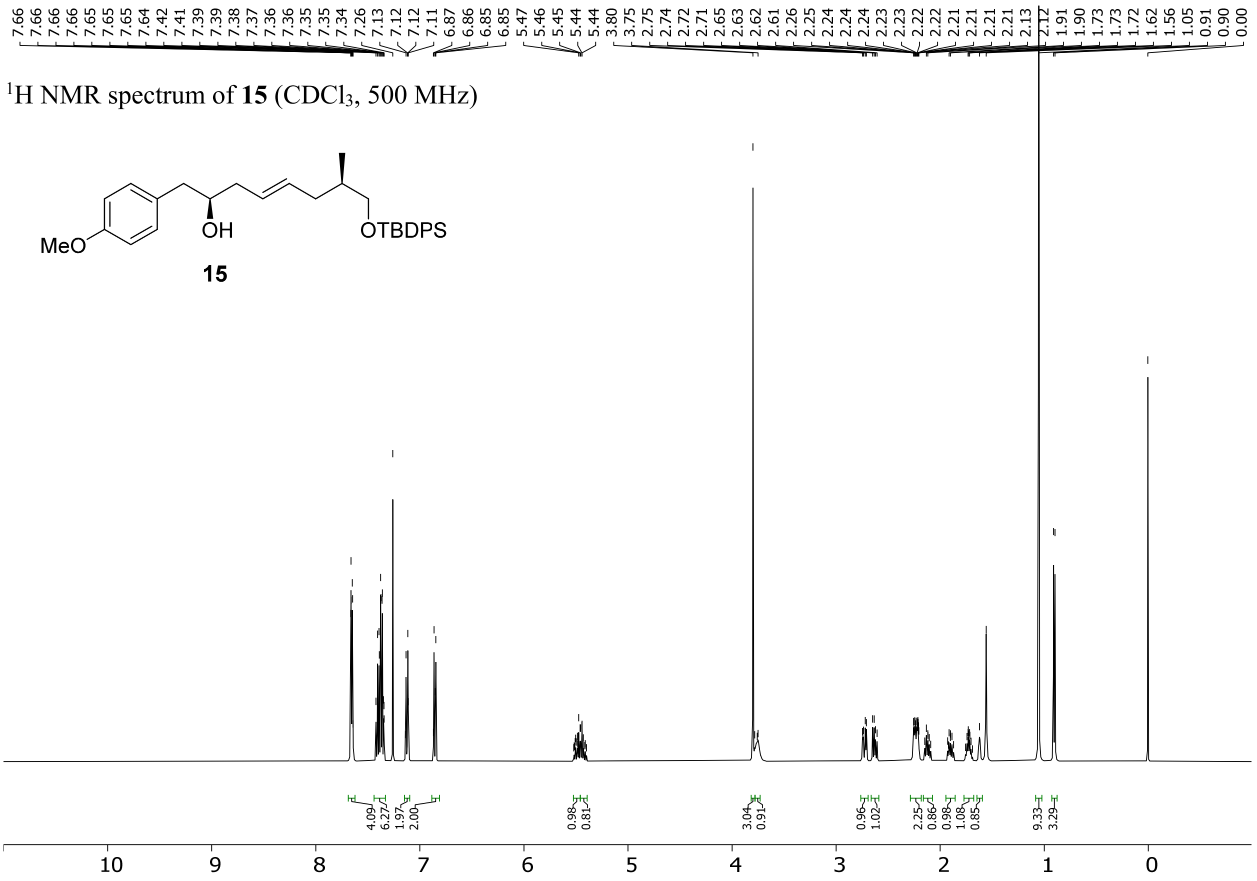

^{1}H NMR spectrum of **15** ($CDCl_3$, 500 MHz)

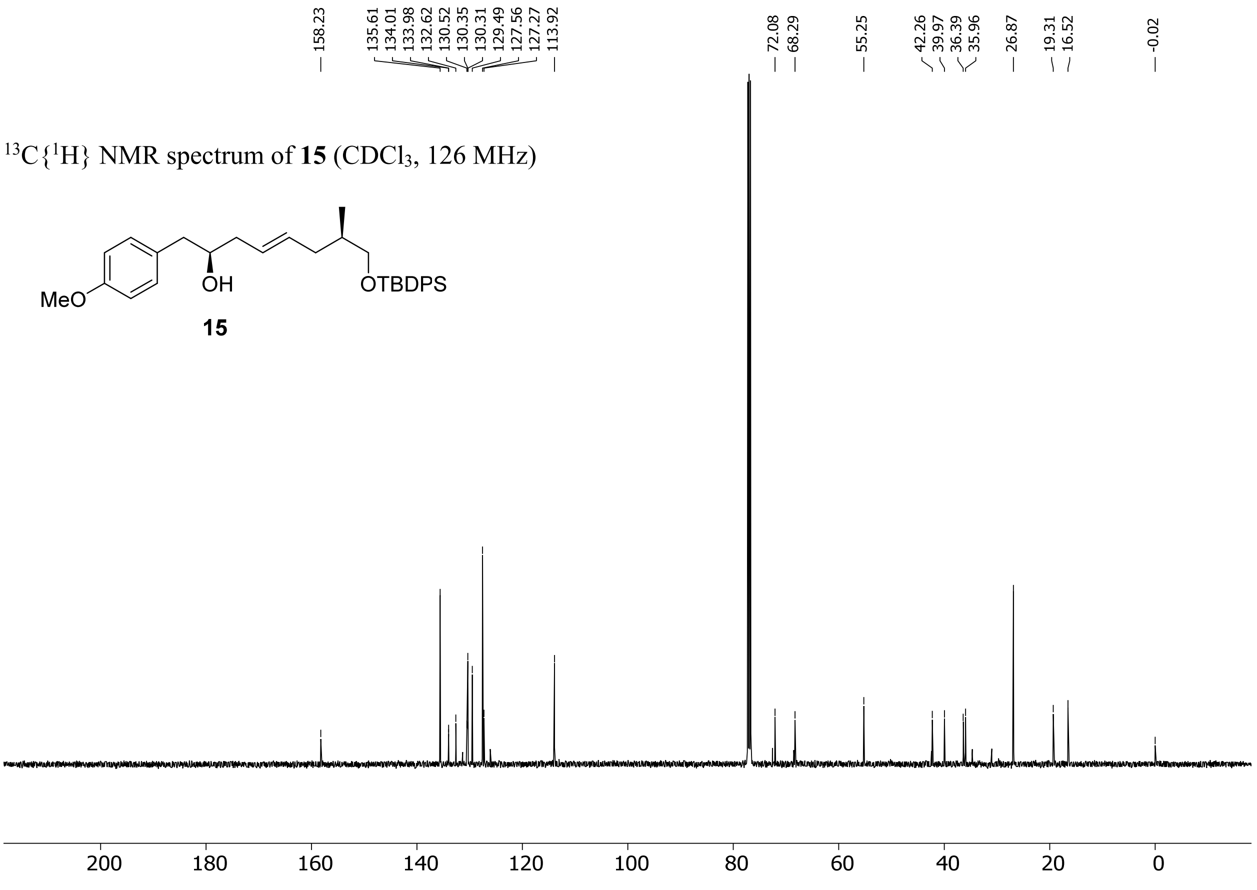

$^{13}C\{^{1}H\}$ NMR spectrum of **15** ($CDCl_3$, 126 MHz)

Table S1. Impact of water equivalents on the $SmI_2(H2O)n$ reduction of **2d** and **2e**.

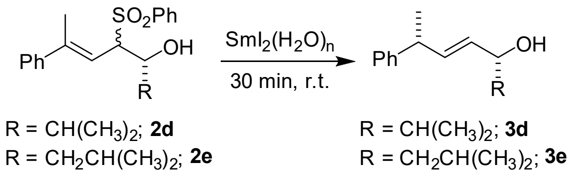

Entry	Compound	Equiv. H_2O[a]	d.r.[b]	Yield (%)[c]
1.	**3d**	5	6.9 : 1	17
2.	**3d**	15	13.5 : 1	58
3.	**3d**	154	11.6 : 1	46
4.	**3e**	15	12.7 : 1	72
5.	**3e**	50	13.3 : 1	52
6.	**3e**	154	13.5 : 1	43

Notes for Table: All reactions were performed using 7 equiv. of SmI_2 in degassed THF at rt under N_2
[a]Relative to SmI_2. [b]Determined by NMR.[c]Isolated yield.

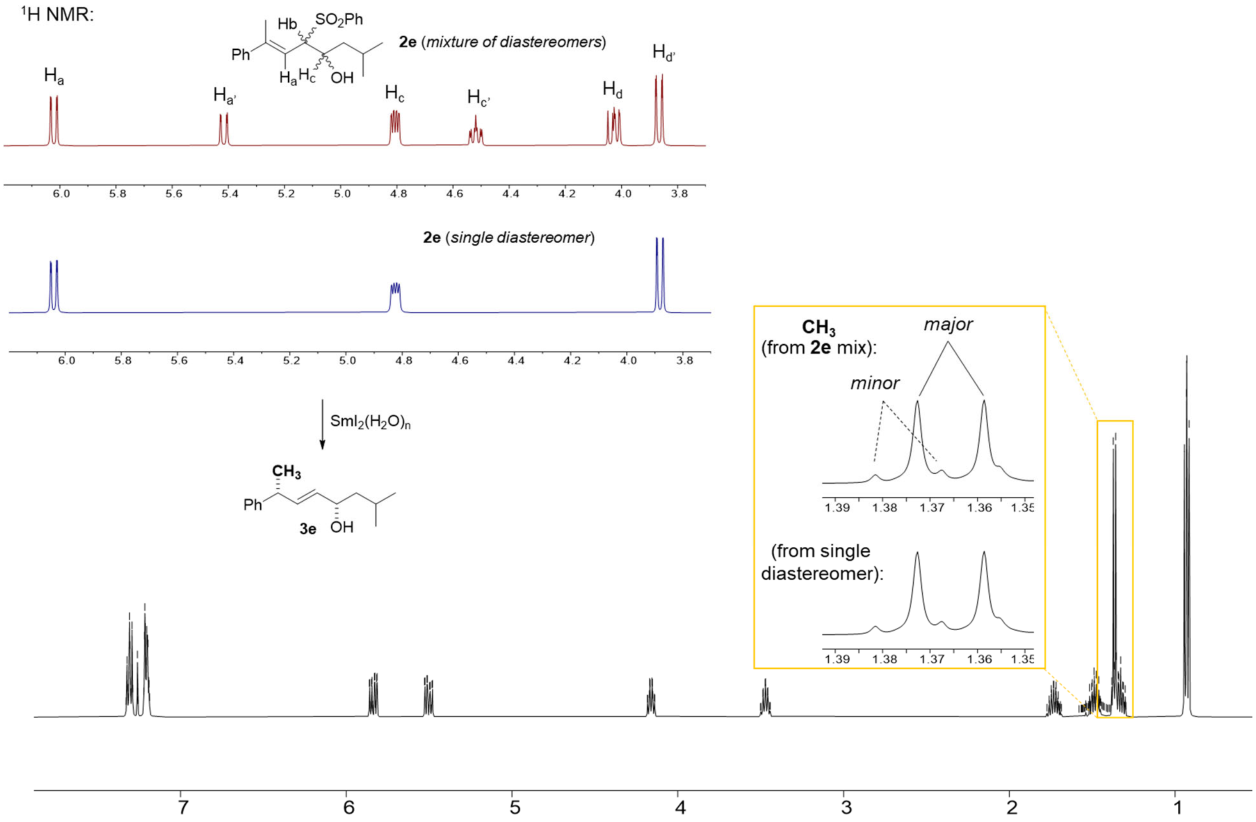

Figure S1. NMR analysis of results from $SmI_2(H_2O)_n$ reduction of **2e** as a mixture of diastereomers (top, red trace) and as a single diastereomer (blue trace). Product **3e** was obtained with the same d.r. indicating no impact of phenyl sulfone stereochemistry on the diastereoselectivity of this reaction.
